# RNA-sequencing suggests extracellular matrix and vasculature dysregulation could impair neurogenesis in schizophrenia cases with elevated inflammation

**DOI:** 10.1038/s41537-024-00466-0

**Published:** 2024-05-04

**Authors:** Hayley F. North, Christin Weissleder, Maina Bitar, Guy Barry, Janice M. Fullerton, Maree J. Webster, Cynthia Shannon Weickert

**Affiliations:** 1 Neuroscience Research Australia, Sydney, NSW Australia; 2 Discipline of Psychiatry and Mental Health, Faculty of Medicine and Health, University of New South Wales, Sydney, NSW Australia; 3 Mechanism and therapy for genetic brain diseases, Institut Imagine, Paris, France; 4 QIMR Berghofer, Herston, QLD Australia; 5 OncoLife Therapeutics, Yeronga, QLD Australia; 6 School of Biomedical Sciences, Faculty of Medicine and Health, University of New South Wales, Sydney, NSW Australia; 7 Laboratory of Brain Research, Stanley Medical Research Institute, 9800 Medical Center Drive, Rockville, MD USA; 8 Department of Neuroscience and Physiology, Upstate Medical University, Syracuse, NY USA

**Keywords:** Molecular neuroscience, Schizophrenia, Schizophrenia

## Abstract

A subgroup of schizophrenia cases with elevated inflammation have reduced neurogenesis markers and increased macrophage density in the human subependymal zone (SEZ; also termed subventricular zone or SVZ) neurogenic niche. Inflammation can impair neurogenesis; however, it is unclear which other pathways are associated with reduced neurogenesis. This research aimed to discover transcriptomic differences between inflammatory subgroups of schizophrenia in the SEZ. Total RNA sequencing was performed on SEZ tissue from schizophrenia cases, designated into low inflammation (*n* = 13) and high inflammation (*n* = 14) subgroups, based on cluster analysis of inflammation marker gene expression. 718 genes were differentially expressed in high compared to low inflammation schizophrenia (FDR *p* < 0.05) and were most significantly over-represented in the pathway ‘Hepatic Fibrosis/Hepatic Stellate-Cell Activation’. Genes in this pathway relate to extracellular matrix stability (including ten collagens) and vascular remodelling suggesting increased angiogenesis. Collagen-IV, a key element of the basement membrane and fractones, had elevated gene expression. Immunohistochemistry revealed novel collagen-IV+ fractone bulbs within the human SEZ hypocellular gap. Considering the extracellular matrix’s regulatory role in SEZ neurogenesis, fibrosis-related alterations in high inflammation schizophrenia may disrupt neurogenesis. Increased angiogenesis could facilitate immune cell transmigration, potentially explaining elevated macrophages in high inflammation schizophrenia. This discovery-driven analysis sheds light on *how* inflammation may contribute to schizophrenia neuropathology in the neurogenic niche.

## Introduction

Schizophrenia is a heterogeneous disorder with unclear aetiology. Recent research implicates a role of inflammation in the neuropathology^1^ and risk for developing schizophrenia^2^. Post-mortem studies consistently find a subgroup of around 40-50% of schizophrenia cases with elevated/“high” inflammation (HI-SCZ) but only around 10% of controls with elevated inflammation across various brain regions such as the prefrontal cortex^3^, the orbital frontal cortex^4^, the midbrain^5^, and the neurogenic subependymal zone (SEZ; also termed subventricular zone)^6,7^. Inflammation and immune cells can alter both the microenvironment^8^ and generation of new neurons from neural stem cells^9^. Bulk RNA sequencing comparing schizophrenia cases and controls revealed increased expression of genes in immune system pathways but decreased expression of neurogenesis transcripts in the human SEZ^10^. The most significantly upregulated gene in schizophrenia was *CD163*^10^, a perivascular macrophage marker. Immunohistochemistry validated an increased density of CD163- positive perivascular macrophages in schizophrenia. Interestingly, increased *CD163* expression was associated with altered mRNA expression of markers for various stages of neurogenesis in schizophrenia^10^. Further investigation revealed that the HI-SCZ subgroup had significantly increased macrophage density and mRNA marker expression of typically peripheral immune cells, increased marker expression of quiescent neural stem cells (*GFAPD*), but decreased expression of markers for neuronal progenitor cells (*DLX6-AS1*) and immature neurons (*DCX*)^6,7^. These findings, along with the HI-SCZ subgroup having reduced expression of phagocytic microglia markers (*P2RY12*, *P2RY13*), were replicated in an independent cohort^7^, thereby corroborating robust changes in neurogenesis and immune cell dysfunction in this schizophrenia subgroup with elevated inflammation. Using a discovery-driven approach, we now asked which molecular mechanisms are associated with neurogenic changes and what other pathways are related to inflammation when comparing HI-SCZ with the low inflammation schizophrenia subgroup (LI-SCZ). While this study focuses on the potential downstream consequences of inflammation in the SEZ, evidence suggests the upstream cause of inflammation in schizophrenia is likely a combination of environmental exposures^11^ and an intrinsic susceptibility to heightened inflammation based on the genetic background^2^. Altered gene expression levels are a molecular phenotype of many common gene polymorphisms^12,13^. Therefore, analysing differences in gene expression may partially capture some upstream causes, in addition to downstream consequences of inflammation.

Hypothesis-driven studies have investigated the relationship between a heightened inflammatory state and markers for various cell types thought to contribute to schizophrenia pathophysiology including astrocytes, microglia, macrophages, endothelial cells and inhibitory interneurons in different brain regions. In both the middle frontal gyrus and midbrain, HI-SCZ subgroups had increased expression of astrocyte makers, which was accompanied by increased astrogliosis in the middle frontal gyrus^5,14^. In the hippocampus, increased inflammation was associated with reduced inhibitory interneuron density^15^ and reduced GABAergic markers^16^. In the prefrontal cortex, reduced inhibitory interneuron transcripts^3^ and increased density of inhibitory interneurons in the underlying white matter^17^ were related to the high inflammatory state in schizophrenia and interneuron markers negatively correlated with cytokine mRNAs^18^. The findings of inflammation-associated alterations in neurogenesis in the SEZ – the birthplace of inhibitory interneurons^6,10^ – suggests that inflammation in the SEZ may impact inhibitory interneuron dysregulation in schizophrenia^19^. While hypothesis-driven studies yielded important insight so far, there is a lack of discovery-driven research identifying the potentially unique and wide-ranging causes or consequences of inflammation in the SEZ in schizophrenia.

Neurogenesis can be regulated by changes in inflammation, cell intrinsic pathways and the extrinsic microenvironment. Inflammation can change the expression of growth factors^20^, such as insulin-like growth factor 1 (IGF1). Reduced *IGF1* expression in the schizophrenia SEZ predicts decreased expression of neuronal progenitor and immature neuron marker mRNAs^21^. Inflammation may have a variety of effects on the unique SEZ microenvironment, which differs from other brain regions due its important role supporting neurogenesis throughout the mammalian lifespan. The SEZ is highly vascularised compared to other brain regions^22^, and neural stem cells have processes extending into blood vessels to receive signals from the periphery including cytokines. Interestingly, proliferating cells accumulate in close proximity to blood vessels and neurogenesis is tightly coupled to the formation and expansion of blood vessels^23^, a process called angiogenesis. The SEZ also has a unique extracellular matrix (ECM) arranged into repetitive units termed ‘fractones’^24^, which have a distinct basal lamina and a network of macrophages and fibroblasts that regulate the ECM and neurogenesis^24,25^. In rodents, fractone ‘speckles/bulbs’ comprising collagen IV and laminins appear between ependymal cells and act as anchors for the apical endfeet of neural stem cells and regulate their proliferation; however, these structures have not been explored in the human SEZ^26–28^. Taken together, the components of the ECM are fundamental to key stages of neurogenesis such as sequestering growth factors, regulating cell division, and providing a scaffold for the migration of newborn neurons^29^.

We hypothesized that a heightened inflammatory state in schizophrenia would lead to a broad range of transcriptional changes in the SEZ niche, which may help to explain the neurogenesis and macrophage changes previously identified in this unique region^6,7,10^. Therefore, the aim of this study was to use deep RNA sequencing as a discovery-driven approach to determine inflammation-associated transcriptional differences within the SEZ in schizophrenia. The advantage of directly comparing two groups of people with schizophrenia is that some potential cofounds of post-mortem studies, like exposure to antipsychotics and impact of a life-time of severe psychiatric illness, occurs in both comparator groups.

## Materials and methods

### Cohort selection

The cohort used for RNA sequencing analysis of inflammatory subgroups was obtained from the Stanley Medical Research Institute (SMRI) and NSW Brain Tissue Resource Centre (BTRC) described in detail previously^6,7,10^. This cohort comprised 27 schizophrenia cases [13 LI-SCZ and 14 HI-SCZ], where inflammation subgroups were previously experimentally-derived using Two-Step Cluster Analysis based on the transcriptional expression of key inflammation markers (*IL6, IL6R, IL1B, IL1R1, IL6ST, CXCL8* and *SERPINA3*) from mRNA extracted from post-mortem tissue dissected from the SEZ, following the same method in the SMRI^6^ and BTRC^7^ cohorts.

Table 1 details all demographics and post-mortem factors of the cohort, including statistical comparisons between demographics of HI-SCZ and LI-SCZ. While the majority of SEZ tissue was from SMRI, tissue from BTRC was included to increase power to detect genes that are differentially expressed between inflammatory subgroups. Cases were selected based primarily on experimentally-derived inflammatory subgroup status, and secondarily on achieving similarity between groups across variables: RNA integrity number (RIN), age, sex, brain pH and post-mortem interval (PMI). The brain banks excluded cases with history of central nervous system disease and/or cases with brain pathology; however having an active peripheral inflammatory disease at time of death was not part of the exclusion criteria, and information regarding use of anti-inflammatory medication around the time of death was not available. This study was carried out in accordance with the Declaration of Helsinki after ethical review at the University of New South Wales (HREC 12435, HC 17826, HC 230253).

**Table 1:** Cohort demographic details and statistical comparisons between inflammatory subgroups.

	LI-SCZ *n* = 13	HI-SCZ *n* = 14	Statistics
Age at death in years	43.23 ± 11.10 (19-61)	50.21 ± 9.55 (35-68)	t(25)= -1.76, *p* = 0.09
pH	6.56 ± 0.26 (6.10-6.91)	6.49 ± 0.22 (6.18-6.93)	t(25) = 0.75, *p* = 0.46
PMI in hours	26.38 ± 11.08 (9-42)	28.82 ± 8.78 (16-47)	t(25) = -0.64, *p* = 0.53
RIN	8.04 ± 0.64 (7.10-9.10)	7.94 ± 0.33 (7.50-8.50)	t(17.75) = 0.52, *p* = 0.61
Sex	8 M/5 F	7 M/7 F	χ2 (1, *N* = 27) = 0.36, *p* = 0.55
Brain hemisphere	5 Left, 8 Right	6 Left, 8 Right	χ2 (1, *N* = 27) = 0.05, *p* = 0.82
Age of onset in years	23.69 ± 7.32	19.43 ± 4.33	t(19.18) = 1.83, *p* = 0.08
Duration of illness in years	19.54 ± 13.24	30.79 ± 8.45	t(25) = -2.65, *p* = 0.01
Lifetime antipsychotic dose^a^ (mg)	59,055 ± 108,506	143,458 ± 146,903	t(25) = -1.69, *p* = 0.10
Antidepressant use	3	3	χ2 (1, *N* = 27) = 0.01, *p* = 0.92
Smoking at time of death	7 Yes, 2 No, 4 Unknown	13 Yes, 0 No, 1 Unknown	
Suicide	4	0	
Cause of death	Cardiac: 7, OD: 1, Pneumonia: 1, Asphyxiation: 2, Trauma: 2	Cardiac: 6, OD:1, Pneumonia: 3, Cirrhosis:1, Pulmonary: 1, Asthma: 1, Pancreatitis:1	

Data are shown as mean ± 1 standard deviation. Ranges are presented in parentheses. ^a^fluphenazine equivalents in mg; *F* female, *HI-SCZ* high inflammation schizophrenia subgroup. *LI-SCZ* low inflammation schizophrenia subgroup, *M* male, *OD* over dose, *PMI* post-mortem interval, *RIN* RNA integrity number.

### Comparison of demographics and post-mortem factors between inflammatory subgroups of schizophrenia

HI-SCZ and LI-SCZ groups did not differ by age, sex, brain pH, PMI, RIN, antidepressant use, lifetime antipsychotic dose, age of disease onset, or brain hemisphere (all *p* ≥ 0.08; Table 1). The HI-SCZ group had a longer duration of illness (30.79 ± 8.45 years) than the LI-SCZ group (19.54 ± 13.24 years; *p* = 0.01), which is discussed in the limitations section. Correlation analyses for age, PMI, RIN, lifetime antipsychotic dose, duration of illness, age of disease onset and the expression of 58 genes of interest identified are detailed in Appendix Table 1. We were unable to statistically test the association of suicide as a mode of death due to voiding the assumption of ‘no zero cells’ in the chi-square test (LI-SCZ: *n* = 4, HI-SCZ: *n* = 0).

### Library preparation and RNA sequencing

The protocols for library preparation and RNA sequencing were previously described^10^. Briefly, all cases selected for sequencing had a RIN ≥ 7.1, indicating high RNA quality. RNA sequencing libraries were constructed using the TruSeq Stranded Total RNA Library Prep Gold kit as per the manufacturer’s protocol (Illumina, Inc., San Diego, CA, USA). Paired-end sequencing of 100 bp read lengths was conducted by the Ramaciotti Centre for Genomics (University of New South Wales, Sydney, Australia) using the NovaSeq 6000 platform (Illumina, Inc.). Half of each pooled sample library was run as replicates (or subsample) on each of two S4 Xp lanes to attain high sequencing depth of paired-end reads while reducing potential batch effects. An average of 109 million unique paired-end reads were attained per sample and all subsamples had at least 40 million sequenced paired-end reads. The average read depth did not differ between LI-SCZ or HI-SCZ groups [t(25) = 0.02, *p* = 0.49).

### Data analysis

Weissleder, et al. ^10^ reports methodological details for demultiplexing, Illumina-specific adapter sequence removal, quality control, and read alignment to the Genome Reference Consortium Human Build 38 transcriptome. To compare differentially expressed (DE) genes between the HI-SCZ and LI-SCZ subgroups, we used the same EdgeR parameters that were previously used in the comparison of controls and schizophrenia cases^10^. Genes with very low expression (i.e. with less than 5 counts per million in 14 cases or more) were excluded from further analysis to ensure sufficient robustness of gene expression for statistical analysis, to minimise false positives from genes with negligible expression in the SEZ, and to reduce multiple testing for poorly expressed genes. Thus, expression levels from 13,048 genes with sufficient expression in the SEZ were retained for analysis. False discovery rate (FDR) adjustment was used to correct for multiple testing, where DE genes with an FDR adjusted *p* value (*q*) <0.05 were considered significant.

### Ingenuity pathway analysis

Ingenuity Pathway Analysis (IPA; Content version: 49932394, Release Date: 2019-11-14, Qiagen, Hilden, Germany) was used for canonical pathway analysis of all DE genes with an FDR *q* < 0.05 (718 genes). Log fold expression change and q values of the DE data were included in the data input. IPA knowledge base was used to determine the activation of pathways, which accounts for the direction of change of a transcript in the data set as well as the directional effect of specific transcripts on others in a pathway. A positive or negative pathway z-score represents the predicted overall activation or deactivation of a pathway respectively. The IPA reference set was the Ingenuity Knowledge Base to include genes only (as opposed to including ‘endogenous chemicals’ for metabolomics data). To test the relationship between DE genes from the most significant pathway and genes known to be markers of different stages of neurogenesis^30^, Spearman’s correlation analysis were run using gene expression in transcripts per million, with a *p* < 0.05 considered statistically significant.

### Collagen IV immunohistochemistry, image acquisition and quantification

Collagen IV immunofluorescence was used to identify collagens associated with the basement membrane and fractones on one section from 18 LI-SCZ cases, 10 HI-SCZ cases and 21 LI control cases from the SMRI cohort. The majority of schizophrenia cases were also used for RNA sequencing. Fresh-frozen brain tissue was sliced into 14 μm thick sections from the anterior-third of the caudate nucleus including the SEZ at the level shown in photographs on pages 121–123 of the *Atlas of the Human Brain*^31^. Tissue was stored at −80 °C on glass slides. Collagen IV immunofluorescence was performed with the protocol described in Weissleder, et al. ^10^ (primary antibody: Abcam ab6586, 1:1000; secondary antibody Invitrogen A-21206 AlexaFluor488 1:500; or AlexaFluor647 1:500). Negative control slides were included by omitting the primary antibody and showed no obvious staining.

For quantification of collagen IV, researchers were blind to the diagnosis and images were acquired on the Nikon Eclipse 80i (Nikon, Tokyo, Japan) with the 10x air objective with numerical aperture 0.45, and mercury lamp with Nikon filter UV-2A for DAPI (blue) and GFP-L for Collagen IV (green). Software used was Stereo Investigator Version 2020 (MBF Bioscience, Williston, VT USA) and camera settings including exposure and gain were kept consistent for each channel. The SEZ was defined as the region of densely packed nuclei between the monolayer of ependymal cells along the lateral wall of the lateral ventricle adjacent to the caudate. Quantification of Collagen IV was performed in Fiji (ImageJ)^32^ using the split channels feature and thresholding in the Collagen IV (green) channel to the minimum grey value of 67 and maximum 255. Collagen IV integrated density was measured as a proportion of total SEZ area (manually outlined in the DAPI channel). Qualitative images and the three dimensional video animation were acquired using z-stacks on LSM800 Zeiss confocal microscope (Zeiss Australia, Lonsdale, SA, AUS).

## Results

### Differential gene expression in HI-SCZ compared to LI-SCZ

**Fig. 1:**
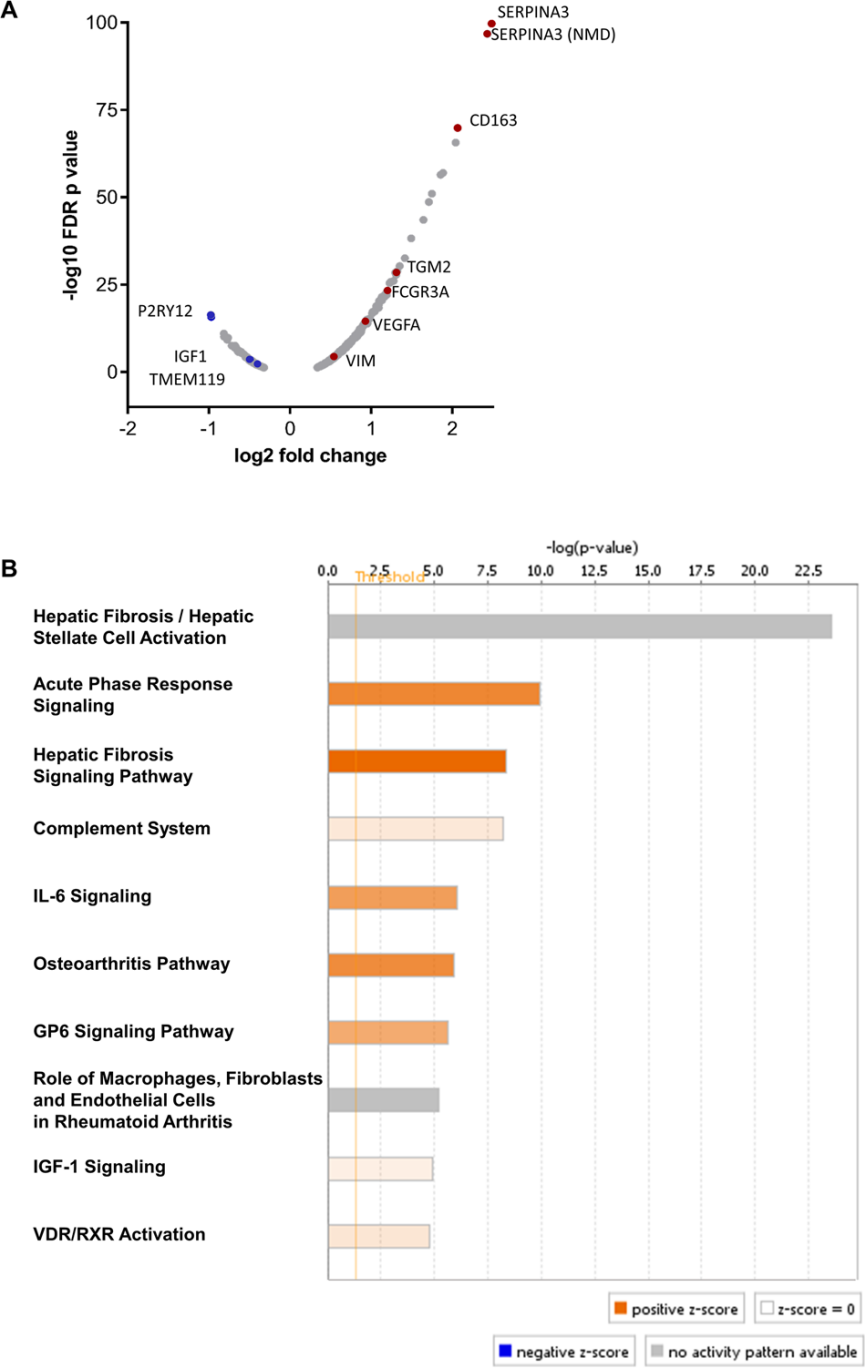
Differentially expressed genes and pathways in HI-SCZ versus LI-SCZ. **A** Volcano plot showing 718 significantly differentially expressed genes in the HI-SCZ compared to LI-SCZ groups based on RNA sequencing data analysis (grey dots). A positive log2 fold change represents increased gene expression in HI-SCZ relative to LI-SCZ (ranging from 25% to 561% increase in expression). Genes of interest in the SEZ, including various cell type markers, are labelled in blue (down-regulation) and red (up-regulation). Note that the second annotated SERPINA3 gene (ENSG00000273259) is reported to undergo nonsense-mediated mRNA decay (NMD), whereas ENSG00000196136 is the primary gene annotation. **B** Top ten most significant canonical pathways identified by IPA based on all differentially expressed genes in HI-SCZ compared to LI-SCZ. Orange colouring indicates overall pathway activation based on positive IPA canonical pathway z-scores, grey colouring (in **B**) represents pathways ineligible for z-score prediction (based on inadequate information in the IPA knowledge base); however, the majority (85%) of genes in the top pathway are increased in HI-SCZ. FDR false-discovery rate adjusted, GP6 Glycoprotein VI, IGF1 insulin-like growth factor 1, VDR/RXR vitamin D receptor/retinoid X receptor.

718 genes were significantly DE between the inflammatory subgroups of schizophrenia with a FDR adjusted *p* value (*q*)<0.05 (Fig. 1A; Appendix Table 2). Of those, 519 (72.3%) were increased and 199 (27.7%) were decreased in HI-SCZ compared to LI-SCZ. Genes with the most significantly increased expression in HI-SCZ compared to LI-SCZ included some previously identified (*SERPINA3, CD163, IL1R1*, and *FCGR3A)*^6^. Novel changes were related to an inflammatory response (*SOCS3*, *CHI3L2, IFITM3, SERPINA1)*, stress signalling (*FKBP5)* and the ECM (*COL4A1, TGM2*) (1.04 < LogFC < 2.50; q < 4.0 × 10^−18^; Fig. 1A). The genes with most significantly decreased expression in HI-SCZ compared to LI-SCZ were related to microglia activation and phagocytosis (*P2RY12, P2RY13)*, the opioid system *(PDYN, PENK)*, neurotransmitter receptors *(DRD3, GLRA2)*, intra- and intercellular transport *(TTR, ABCG2, SLC13A5)*, and stem cell regulation *(SHISA2)* (−0.97 < LogFC < −0.63; *q* < 2.3 × 10^−6^; Fig. 1A). Of note, the expression of the cell type markers for microglia (*TMEM119*, *CX3CR1)* were decreased in HI-SCZ compared to LI-SCZ (LogFC = -0.35, −0.54; *q* = 0.034, 0.04 × 10^−3^, respectively; Fig. 1A).

### Pathway analysis revealed inflammation-related changes to the SEZ niche environment

To determine the functional relevance of DE genes, all 718 genes (q < 0.05) were used as input for Ingenuity Pathway Analysis (Fig. 1B**;** Appendix Table 3). Immune-related pathways (e.g. ‘IL-6 signalling’ and ‘Complement System’) were significantly overrepresented in DE genes and had increased activation (all *p* < 0.0001) including ‘Agranulocyte Adhesion and Diapedesis’ (*p* = 0.005), confirming that previously reported changes in schizophrenia compared to controls^10^ were driven by the HI-SCZ subgroup. Because we expected immune-related pathways to be elevated in the comparison of HI-SCZ vs LI-SCZ, they will not be a focus herein due to the selection of these groups based on expression of key immune genes.

**Fig. 2:**
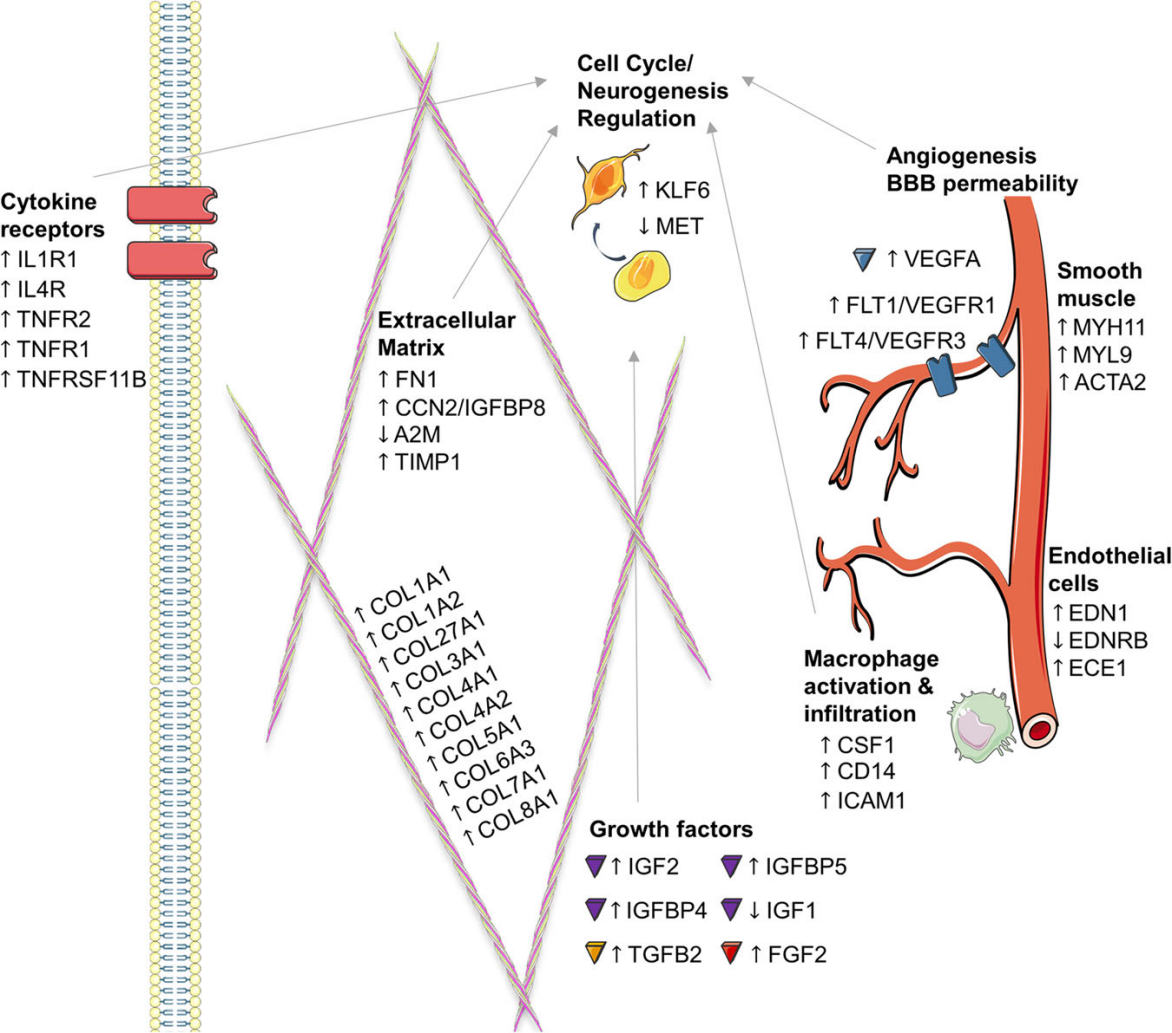
Schematic of genes within the ‘Hepatic Fibrosis / Hepatic Stellate Cell Activation’ pathway that exhibit differential gene expression in HI-SCZ versus LI-SCZ. Genes in this top pathway identified by IPA have broad functional roles including regulation of the extracellular matrix, angiogenesis, growth factors, and immune cell function, all of which can influence the different cellular developmental stages of neurogenesis. Arrows next to gene names represent significantly increased (upward arrow) or decreased (downward arrow) gene expression in HI-SCZ compared to LI-SCZ.

Genes in the most significant pathway ‘Hepatic Fibrosis / Hepatic Stellate Cell Activation’ were related to the ECM, growth factors and angiogenesis, and were mostly upregulated in HI-SCZ compared to LI-SCZ (*p* = 0.02 × 10^−22^; Fig. 2). The third most significant pathway, ‘Hepatic Fibrosis Signalling Pathway’, has similar functional implications with many overlapping transcripts. Therefore, further investigation of ‘Hepatic Fibrosis / Hepatic Stellate Cell Activation’, will be the focus. Of note, integrins and laminins are functionally related to the ‘Hepatic Fibrosis / Hepatic Stellate Cell Activation’ pathway and were also increased in HI-SCZ compared to LI-SCZ (*ITGB4, ITGA5, ITGA8, ITGA9, ITGB8, LAMA5, LAMA2;* 0.34 < LogFC < 0.65; *q* < 0.04). Other significant pathways have relevance to the regulation of neurogenesis and schizophrenia, including ‘IGF-1 Signalling’ and ‘VDR/RXR Activation’ (vitamin D receptor and retinoid X receptor pathways) (both *p* < 0.0001).

### Key genes indicating ECM stiffening were upregulated in HI-SCZ

**Fig. 3:**
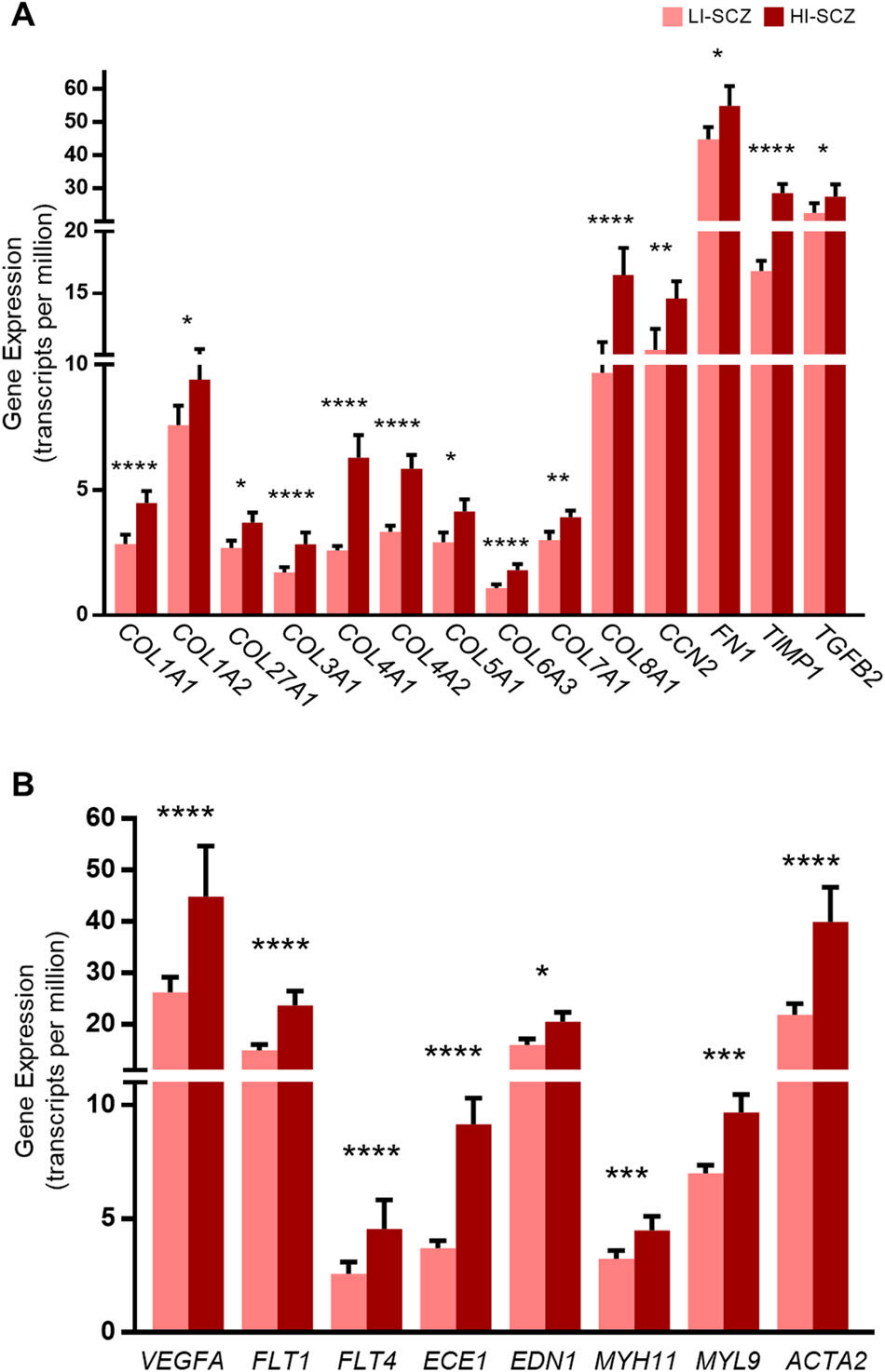
Increased expression of genes contributing to extracellular matrix and angiogenesis in the top differentially expressed pathway ‘Hepatic Fibrosis / Hepatic Stellate Cell Activation’. **A** Ten collagen genes had significantly increased expression in HI-SCZ compared to LI-SCZ, along with four other genes (CCN2, FN1, TIMP1and TGFB2) for which increased expression is typical in fibrosis and extracellular matrix stiffening. **B** *VEGFA* and its receptors *FLT1* and *FLT4*, which initiate angiogenesis, were significantly increased in HI-SCZ compared to LI-SCZ, along with markers for endothelial (*END1*, *ECE1*) and smooth muscle cells (*MYH11*, *MYL9, ACTA2*). Data is represented as the mean with standard error of the mean; *q < 0.05; **q < 0.01; ***q < 0.001; ****q < 0.00001.

The key overexpressed mRNAs in the pathway ‘Hepatic Fibrosis / Hepatic Stellate Cell Activation’ that indicate increased ECM stiffening in the neurogenic niche included 10 collagen genes (*COL1A1, COL1A2, COL27A1, COL3A1, COL4A1, COL4A2, COL5A1, COL6A3, COL7A1, COL8A1;* 0.35 < LogFC < 1.32; *q* < 0.03). Other indications of ECM stiffening in this pathway were an upregulation of a transforming growth factor beta isoform that initiates fibrosis (*TGFB2*; LogFC = 0.38, *q* = 0.01)^33^; an ECM component fibronectin (*FN1*; LogFC = 0.33; *q* = 0.04); cellular communication network factor 2 (*CCN2*; LogFC = 0.49; *q* = 0.03 × 10^-2^); and TIMP metallopeptidase inhibitor 1, which inhibits degradation of the ECM (*TIMP1;* LogFC = 0.77; *q* = 0.04 × 10^-8^; Fig. 3a). Considering that fibroblasts are the primary producers of ECM components^34^ and are integral members of the SEZ fractone network^25^, and that type A pericytes expressing *SLC1A3* produce central nervous system fibrotic scarring^35^, we investigated eight putative markers for those cell types (*HAVCR2*, *COL3A1*, *COL1A2, S100A4*, *THY1*, *DPP4*, *DLK1, SLC1A3*)^36^. Of those, two fibroblast markers were increased in HI-SCZ compared to LI-SCZ (*COL1A2, COL3A1*; 0.35 < LogFC < 0.69; *q* < 0.03).

Correlation analyses between the expression of differentially expressed ECM genes in this pathway and the expression of marker genes for neurogenesis^10,30^ Appendix Table 4 revealed moderate (rho > 0.5) to strong (rho > 0.8) relationships with markers of different stages of neurogenesis. Among the significant correlations (all *p* < 0.05), ECM mRNAs predominately positively correlated with markers for stem cells (*SOX2, PAX6, GLI3, MKI67, ASCL1*) but negatively correlated with markers for neuroblasts (*TMDB10, NNAT, FGFR3, CEND1*) and immature neurons (*DLX6-AS1*).

### Increased angiogenesis-related genes were associated with markers indicative of immune cell infiltration

A number of transcriptional changes indicating angiogenesis (*VEGFA*, *FLT1*, *FLT4*; 0.68 < LogFC < 0.94; *q* < 0.02), including markers for endothelial (*END1*, *ECE1*; 0.37 < LogFC < 1.27; *q* < 2.80 × 10^−8^) and smooth muscle cells (*MYH11*, *MYL9, ACTA2*; 0.46 < LogFC < 0.88; *q* < 0.0009; Fig. 3b) had increased levels in HI-SCZ. These mRNAs were part of the most significant pathway ‘Hepatic Fibrosis/Hepatic Stellate Cell Activation’. Considering the prominent role of *VEGFA* and its receptor *FLT1* in angiogenesis^8^, and increased immune cell infiltration corresponding with angiogenesis^37^, we analysed their gene expression in relation to macrophage marker, *CD163*, and a marker of blood vessel luminal cell adhesion, *ICAM1*. *CD163* and *ICAM1* mRNAs both positively correlated with *VEGFA* and *FLT1* in schizophrenia (all Spearman’s rho ≥0.47, *p* ≤ 0.01).

Correlation analyses between differentially expressed angiogenesis genes and the expression of marker genes for neurogenesis (Appendix Table 5) revealed moderate (rho > 0.5) relationships, including positive correlations with neural stem cell markers (*SOX2*, *PAX6*, *GLI1*, *MKI67*), and negative correlations with neuroblast markers (*NNAT*, *FGFR3*).

### Collagen IV is localised around blood vessels and in bulbs adjacent to the ependymal layer

**Fig. 4:**
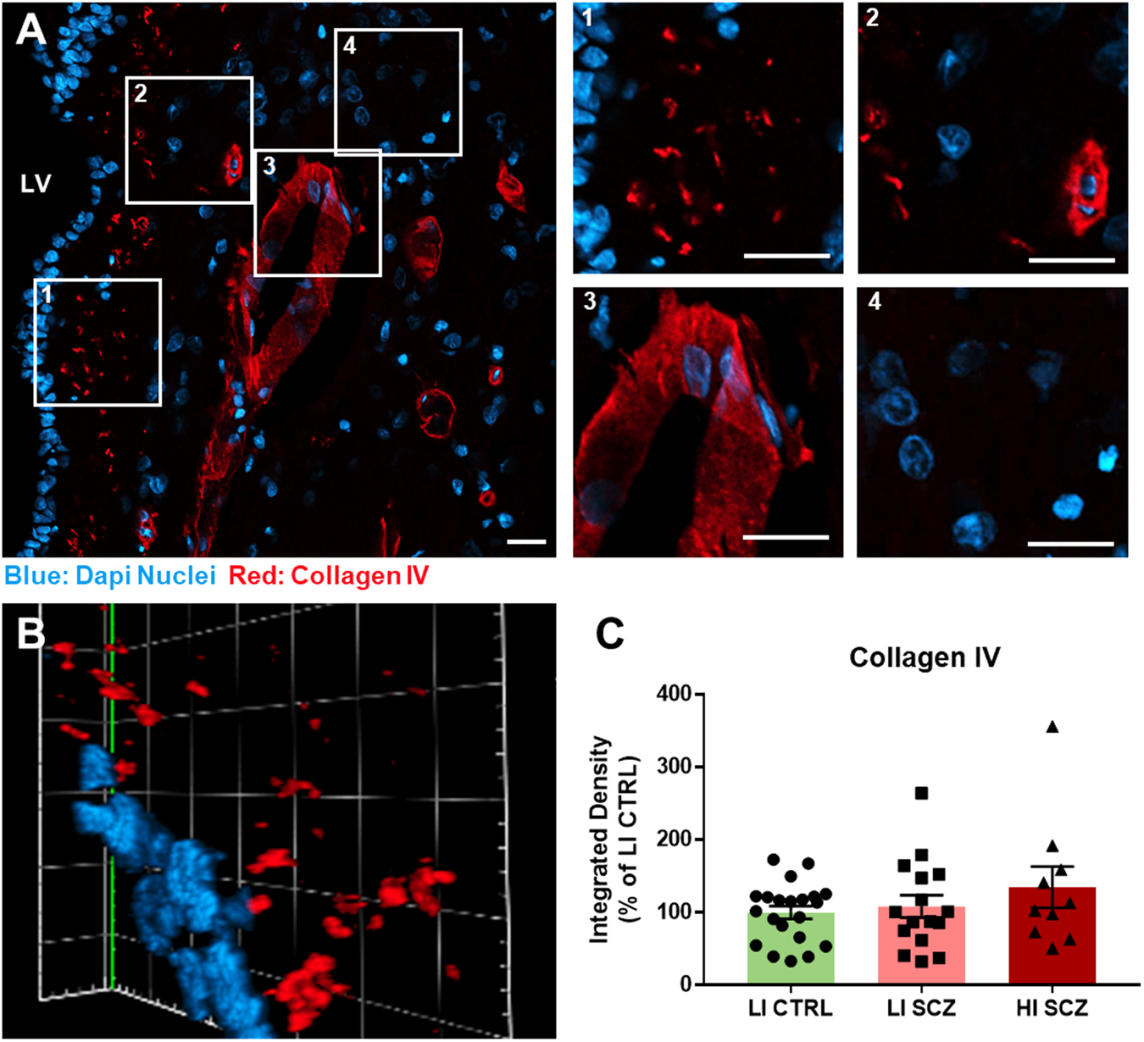
Collagen IV is abundant around blood vessels and in bulbs in the human SEZ hypocellular gap. **A** A representative image of typical collagen IV (red) localisation in the human SEZ (DAPI positive nuclei in blue) from a control. Inset 1 shows fractone bulbs in the hypocellylar gap. Inset 2 and 3 demonstrate collagen IV staining surrounding a small and large blood vessel. Inset 4 shows areas devoid of collagen IV. **B** Collagen IV bulbs from side angle of a z-stack, each mark on the scale is 5 μm. **C** Quantification of collagen IV integrated density in the entire SEZ from one section per case demonstrated a ~ 25% increase in collagen IV in HI-SCZ; however, high within group variability was observed (21 LI control cases, 18 LI-SCZ cases, 10 HI-SCZ cases). All scale bars are 20 μm. Data is displayed as mean ± standard error of the mean. LI CTRL low inflammation control, LI SCZ low inflammation schizophrenia, HI SCZ high inflammation schizophrenia, LV lateral ventricle.

Unique basement membrane structures called fractones, that are comprised of collagen IV^24^, are a key feature of the SEZ microenvironment. Considering we found significant elevations in *COL4A1* and *COL4A2* mRNA expression, we used immunohistochemistry to identify and characterise collagen IV protein distribution in the human SEZ. Collagen IV was abundantly expressed around the vasculature, with a novel pattern of collagen IV speckles/bulbs throughout the hypocellular gap adjacent to the ependymal layer in controls and schizophrenia subgroups (Fig. 4a and insets). The collagen IV bulbs had a three-dimensional structure (Fig. 4b, and video in **Appendix 1**) and were not present elsewhere in the SEZ and adjacent caudate nucleus. Collagen IV density in the SEZ (Fig. 4c) was increased by around 25% in HI- compared to LI-SCZ; however, there was large within group variability limiting statistical interpretation [achieved power: 0.18; ANCOVA (age) F(2,46) = 1.09, *p* = 0.345]. While the pathway analysis indicated ‘fibrosis’, there were no obvious regions of the SEZ devoid of DAPI+ nuclei and with collagen IV coverage that would indicate permanent fibrotic scarring.

### Relationships between DE genes and clinical factors

Correlation analyses between demographic and clinical factors (age, RIN, PMI, lifetime antipsychotic dose, age of onset, and duration of illness) with the expression (in TPM) of 58 genes that are highlighted throughout the results section are presented in Appendix Table 1. Expression of the vast majority of genes relating to the ECM and angiogenesis did not correlate with lifetime antipsychotic dose. Microglia markers all negatively correlated with lifetime antipsychotic dose (*P2RY12*, *P2RY13*, *TMEM119* and *CX3CR1;* rho ≤ −0.49, *p* ≤ 0.01), whereas some immune genes positively correlated with lifetime antipsychotic dose (*ICAM1, IFITM3, IL1R1, SERPINA1, SERPINA3, SOCS3*; rho≥0.39, *p* ≤ 0.03).

## Discussion

Many of the most significantly increased genes in HI-SCZ compared to LI-SCZ were inflammation-related, which confirms the validity of the cluster analysis previously employed to designate cases into inflammatory subgroups. It shows that the inflammatory subgroups are reflective of a wider network of inflammatory-related expression differences, thereby providing a tool to demonstrate *how* the inflammatory state differs in schizophrenia. Microglia markers were among the most significantly downregulated genes in HI-SCZ, which aligns with previous findings of reduced microglial markers in the SEZ in HI-SCZ^6,7^. This discovery-driven work demonstrated a number of novel findings. First, we found very significant overrepresentation of increased gene expression related to the ECM in HI-SCZ. The increase in 10 collagen transcripts is expected to result in collagen deposition around stem and progenitor cells^38^, which likely alters the niche environment by creating a stiffer, potentially more fibrotic state. Upregulation of gene expression related to angiogenesis in HI-SCZ suggests the formation of new blood vessels. During angiogenesis, blood vessels are expected to be more permeable^8^. Deposition of collagen IV was typically surrounding blood vessels, but it also formed unique bulb like structures in the hypocellular gap between the ependymal later and astrocytic ribbon where neural stem cells reside. They are reminiscent of the collagen IV-positive fractone bulbs described in the rodent SEZ as being located between the ependymal cells and with functions relating to stem cell maintenance. Considering the hypocellular gap is a unique feature of the human SEZ^39^, these fractone bulbs in the hypocellular gap in humans and may regulate stem cells in the adjacent astrocytic ribbon. Since there is an intimate anatomical and functional relationship between immature cells and the microenvironment, these SEZ architectural changes may contribute to the previously reported inflammation-related changes in neurogenesis in schizophrenia^6,7^.

### Inflammation may alter the ECM of the SEZ neurogenic niche in schizophrenia

Our discovery-driven approach highlighted that SEZ inflammation in schizophrenia seems to have its greatest influence on components of the niche microenvironment such as the ECM. In our previous study comparing the SEZ transcriptome between controls and schizophrenia, five ECM related genes were also increased in schizophrenia compared to controls (*COL4A1, COL4A2, COL3A1, TGFBI, TIMP1)*^10^, and this study demonstrates those changes were mainly occurring in the HI-SCZ subgroup. The ECM has important functional roles in neurogenesis, including facilitating cell-to-cell communication, growth factor signalling, and neuronal cell differentiation and migration^24,40^, thus ECM changes may influence a range of neurogenic functions. The most significant pathway enriched in DE genes in HI-SCZ compared to LI-SCZ had upregulated genes indicative of fibrosis, whereby an accumulation of ECM proteins, such as collagens, may create scar tissue potentially in response to chronic inflammation^34^. While immunohistochemistry did not indicate typical fibrotic scarring that would be expected in the place of SEZ cells^35^, the important new perspective is that increased inflammation may lead to stiffening of the brain extracellular environment. Fibrotic changes in other tissues are not a result of acute inflammation^34^. If the putative fibrotic-like changes in the brain are related to chronic inflammation, then we may expect to find evidence of fibrotic change in other tissues in schizophrenia. Indeed, in schizophrenia patients, structural and functional cardiac deficits reflect myocardial fibrosis^41^, which is known to be mediated by pro-inflammatory cytokines such as IL-6^42^. While this study is the first to demonstrate a link between transcriptional changes in fibrosis and cytokines in brain tissue in schizophrenia, evidence shows that fibrosis can be both a cause and consequence of inflammation.

Increased ECM fibrosis interacts with macrophages to regulate their accumulation and retention in lung tissue^43^, which may explain why we previously found increased macrophage density in the brain in the SEZ in HI-SCZ^6,10^. This is also supported by the finding of increased infiltration of immune cells into the human brain in multiple sclerosis lesions with traits of fibrosis and collagen accumulation^44^. Conversely, inflammation also causes increased ECM accumulation leading to fibrosis^34^. This process begins with TGF-β inducing myofibroblasts to produce excessive ECM proteins, particularly fibrillar collagens, and inhibit ECM degradation^33^. However, future research is needed to determine if this process occurs in the brain and to identify which cell type is upregulating collagens in the human SEZ. Accompanying neuroinflammation in rodent experimental autoimmune encephalomyelitis, an accumulation of ECM has been shown to form a fibrotic scar due to increased infiltration of fibroblasts to the spinal cord parenchyma^45^. While fibroblasts are already present and in contact with macrophages in the perivascular layer of SEZ fractones^25^, it remains to be determined whether fibroblasts increase in number or infiltrate the human SEZ parenchyma in HI-SCZ. It is speculated that fibroblasts, in conjunction with macrophages, release growth factors and cytokines to initiate proliferation, differentiation and migration of newborn neurons in the SEZ^25^. These important functional implications, and the finding that two of seven markers for fibroblasts were increased in HI-SCZ, highlight the need for investigating the contribution of this cell type. Regardless of the status of fibroblasts, increased expression of ten collagen mRNAs, including those encoding classical fibrillar collagens (I, II, III and V)^46^, likely alter the stiffness of the ECM in HI-SCZ. The SEZ ECM is already stiffer than other brain regions^40^ and this stiffness is thought to influence neurogenesis through mechanosensitive ion channels on neural stem cells affecting their self-renewal and differentiation^47^, and by potentially influencing neurite outgrowth^48^. The negative correlations between ECM mRNA and markers for neuroblasts and immature neurons may indicate reduced neuronal differentiation in a potentially stiffer SEZ. Experimental findings by Kjell, et al.^40^ show that quiescent stem cells in the SEZ are a source of many ECM proteins. Therefore, our previous finding of increased stem cell quiescence marker (*GFAPD*) in HI-SCZ^6,7^ may underlie the concordance between stem cell markers and increased ECM transcripts supported here by the positive correlations. Overall, the increased expression of transcripts indicating an altered SEZ ECM could influence various stages of neurogenesis and inflammatory processes in HI-SCZ and warrants further investigation.

### Inflammation may increase angiogenesis and blood-brain-barrier (BBB) permeability in the SEZ neurogenic niche in schizophrenia

The pathway analysis suggested increased inflammation in the SEZ niche also leads to increased angiogenesis. Angiogenesis-promoting growth factor *VEGFA* and two of the three VEGF receptors (*FLT1*, *FLT4*) as well as expression of markers for endothelial (*END1*, *ECE1*) and smooth muscle cells (*MYH11*, *MYL9, ACTA2*) were increased in HI-SCZ^49^. In the SEZ in schizophrenia compared to controls, *VEGFA, FLT1, MYH11, ACTA2* expression was also increased^10^, which we now understand is primarily due to the HI-SCZ subgroup. In the SEZ in HI-SCZ, *VEGFA* could directly regulate gliogenesis and neurogenesis because it binds to the VEGFR2 on BrdU^+^ proliferating cells and subsequently promotes differentiation into astrocytes and immature neurons^50^. However, in our study, the positive correlations between markers for angiogenesis and neural stem cells but negative correlations between markers for angiogenesis and neuroblasts, does not align with proposal that VEGFA is directly facilitating differentiation of stem cells into immature neurons in the adult human SEZ. Through its promotion of angiogenesis, *VEGFA* could also be indirectly regulating neurogenesis in HI-SCZ as newborn endothelial cells release growth factors, such as brain-derived neurotrophic factor, that regulate neurogenic cells in proximity to sites of angiogenesis^51^. One of the only other studies of the relationship between angiogenesis and neurogenesis in human post-mortem brain tissue showed a very strong positive correlation between neural progenitor cells and capillary area in the dentate gyrus neurogenic niche in aging^52^. Interestingly, the interaction between blood vessels and progenitor cells is mediated by integrins and laminins, and when integrin/laminin-mediated binding is blocked, progenitor proliferation is increased^53^. Considering we found increased integrins, laminins and vascular components in HI-SCZ, this could possibly explain reduced markers of neuronal progenitor cells (*ASCL1, DLX6-AS1*) in this subgroup^6,7^.

An increase in markers of angiogenesis may indicate higher vascular permeability. Increased leakiness in the SEZ vasculature^22^ is proposed to induce dynamic changes in neural stem cells, which typically reside proximal to blood vessels, in response to changes in the periphery^54^. A recent study exemplified this relationship between the SEZ and periphery by finding increased deposition of liver-produced fibrinogen from the blood into SEZ tissue post-injury, which induced astrocytic differentiation in rodents^55^. Further, peripheral administration of fibroblast growth factor 2 increases neurogenesis in the rodent SEZ to a far greater extent than in the hippocampus, demonstrating the particularly important role of peripheral signals in SEZ neurogenesis^56^. Therefore, vascular changes during angiogenesis in HI-SCZ may decrease gate keeping of peripheral molecules and highlights the need to investigate these neurogenesis-regulating factors.

In the SEZ in HI-SCZ, a cyclical causal relationship could exist between inflammation and angiogenesis because immune cells secrete angiogenic factors and activated endothelial cells facilitate the tethering and transmigration of immune cells into the parenchyma [for review see Costa, et al. ^37^]. This relationship between immune cell transmigration and angiogenesis is particularly facilitated by a number of adhesion molecules, such as e-selectin, ICAM-1 and vascular cell adhesion molecule 1, which are more highly expressed by angiogenic endothelial cells^57^. Further, VEGFA increases BBB permeability that facilitates transmigration of immune cells, particularly monocytes, in the cortex before and during increased angiogenesis^8^. Considering that the SEZ vasculature is already more permeable than the cortex^22^, the increased immune cell markers and macrophage density in HI-SCZ^6,7^ may arise from increased *VEGF* and angiogenesis. These interpretations are supported by the relatively strong correlations between *VEGFA*, *FLT1* and both *CD163* and *ICAM1* mRNAs as well as the positive activation of the ‘agranulocyte adhesion and diapedesis’ pathway in HI-SCZ. Therefore, future studies quantifying angiogenesis in the SEZ with respect to inflammation, peripheral immune cell types and density will be important.

### Limitations and future directions

The depth of understanding obtained from sequencing the entire transcriptome in human brain tissue from people with schizophrenia based on their immune status has several limitations. We sequenced homogenised tissue comprising numerous individual cell types, each with unique expression profiles, so future use of single-cell or single nuclei RNA sequencing would enable the characterisation of specific immune cells, blood vessels, neurogenic cell types and immature neurons in the SEZ and differentiated neurons the adjacent caudate in schizophrenia with respect to the inflammatory state. While studies in human tissue are particularly valuable when studying complex psychiatric disorders, they are limited to being descriptive and cannot ascertain cause and effect. In the SEZ, only 10% of controls (*n* = 3) were identified as high inflammation^6^ limiting statistical analyses. We are therefore unable to determine whether findings in this study are related to inflammation in general or specific to inflammation in schizophrenia. Nonetheless, considering a significantly larger proportion of schizophrenia cases have elevated inflammation in the SEZ, the findings still give insight into neuropathological differences between subgroups within schizophrenia. Further, in a larger cohort study in the prefrontal cortex, where 17% of controls (*n* = 12) were designated as high inflammation, we were able to directly compare high inflammation controls and schizophrenia cases. We found significantly reduced microglia (*CD11C*) but increased macrophage (*CD163*, *CD64*) mRNAs in high inflammation schizophrenia compared to high inflammation controls^58^. These results align with *CD163* being the most differentially expressed mRNA detected by RNAseq in the SEZ of people with schizophrenia compared to controls^10^ and suggests that the high inflammation state may be more related to macrophages in schizophrenia.

Although we cannot discount the potentially significant effect of antipsychotic use on transcriptomic profiles, the vast majority of genes relating to angiogenesis and the ECM were not significantly related to lifetime antipsychotic dose, suggesting medication exposure is unlikely to be the driver of the main findings. Additionally, both comparison groups received antipsychotics throughout their lifetime. There was no information available regarding usage of other medication such as anti-inflammatory medication around time of death. While suicide can contribute to molecular changes in post-mortem research^59^, this study had insufficient data to explore the potential relationship between suicide and changes in the SEZ microenvironment.

While HI-SCZ and LI-SCZ groups were matched on almost all key demographic and tissue quality factors, duration of illness was significantly longer for the HI-SCZ group. This could be a confounding factor, in that a longer duration of illness indicates a longer exposure to the stressful disease state. Duration of illness is inextricably linked with age and inflammation is known to increase with age^60^. Thus, age could potentially influence inflammation levels in these cases despite age not being significantly different between inflammatory subgroups. Longer duration of illness in the HI-SCZ group could also suggest a potentially progressive nature of inflammation in schizophrenia, which has been proposed by others^61^. On the other hand, there is evidence for inflammation being most pronounced at disease onset^62^, predicting conversion from clinical high risk to psychosis^63^, and reducing over time^64^ and with antipsychotic treatments^65^. Future longitudinal studies of inflammation in living patients across the duration of illness will be important to determine the timing and nature of inflammation in schizophrenia.

## Conclusion

This study disentangled inflammation-associated changes in the SEZ neurogenic niche in schizophrenia using a transcriptomic approach and uncovered important questions for future research. Our findings suggest that increased ECM stiffness and angiogenesis are concomitant with inflammation in schizophrenia in the neurogenic microenvironment. This background may set the stage for, or be a consequence of, increased stem cell quiescence, immature neuron deficits and immune cell infiltration. These findings begin to uncover broader consequences of neuroinflammation in the SEZ in schizophrenia and may be a step towards developing more personalised treatment options.

### Supplementary information

Appendix Table 1

Appendix Table 2

Appendix Table 3

Appendix Table 4

Appendix Table 5

Supplementary Video

## Data Availability

Raw sequencing data from this study are available on request and stored in the UNSW data archive at www.dataarchive.unsw.edu.au under Research Data Management Plan reference number D0425180. The data will also be available via the database of Genotypes and Phenotypes (dbGaP) under the study name: Neuroinflammation and Neurogenesis in Schizophrenia.
