# Supplementary material for: RNA-sequencing suggests extracellular matrix and vasculature dysregulation could impair neurogenesis in schizophrenia cases with elevated inflammation
Source: Schizophrenia (Heidelb). 2024 May 4;10(1):50. doi: 10.1038/s41537-024-00466-0 (PMC11069512; doi:10.1038/s41537-024-00466-0)
Supplement: Supplementary file 1 — Appendix Table 1 [file 41537_2024_466_MOESM1_ESM.docx]

**Appendix Table A1: Spearman's Rho Correlations between genes of interest and clinical or demographic features.**

|  | **Correlation Coefficient** | | | | | | **Sig. (2-tailed)** | | | | | |
| --- | --- | --- | --- | --- | --- | --- | --- | --- | --- | --- | --- | --- |
| **Gene** | **Age** | **RIN** | **PMI** | **Lifetime antipsychotic dose (mg)** | **Age of onset** | **Duration of illness** | **Age** | **RIN** | **PMI** | **Lifetime antipsychotic dose (mg)** | **Age of onset** | **Duration of illness** |
| ABCG2 | -0.194 | -0.132 | -.472* | -.513** | 0.356 | -0.368 | 0.332 | 0.513 | **0.013** | **0.006** | 0.068 | 0.059 |
| ACTA2 | -0.069 | -0.211 | -0.095 | 0.252 | -0.279 | 0.092 | 0.732 | 0.290 | 0.638 | 0.204 | 0.158 | 0.647 |
| CD163 | -0.016 | -0.035 | 0.162 | 0.352 | -0.367 | 0.158 | 0.937 | 0.862 | 0.421 | 0.072 | 0.060 | 0.430 |
| CHI3L2 | -0.120 | -0.016 | -0.006 | .385* | -0.296 | 0.040 | 0.552 | 0.936 | 0.976 | **0.047** | 0.134 | 0.845 |
| COL1A1 | 0.209 | -.483* | 0.169 | 0.349 | -0.134 | 0.248 | 0.295 | **0.011** | 0.399 | 0.074 | 0.505 | 0.212 |
| COL1A2 | -0.207 | -0.151 | -0.094 | -0.013 | -0.242 | -0.070 | 0.301 | 0.453 | 0.642 | 0.947 | 0.224 | 0.728 |
| COL27A1 | 0.187 | -.461* | -0.029 | 0.194 | -0.144 | 0.214 | 0.350 | **0.015** | 0.886 | 0.333 | 0.472 | 0.285 |
| COL3A1 | -0.223 | -0.198 | -0.060 | -0.025 | -0.343 | -0.036 | 0.264 | 0.322 | 0.765 | 0.903 | 0.080 | 0.859 |
| COL4A1 | 0.070 | -0.059 | 0.152 | .524** | -.496** | 0.277 | 0.728 | 0.769 | 0.449 | **0.005** | **0.009** | 0.163 |
| COL4A2 | -0.108 | -0.078 | 0.163 | 0.364 | -0.326 | 0.077 | 0.592 | 0.698 | 0.416 | 0.062 | 0.098 | 0.703 |
| COL5A1 | 0.261 | -.430* | -0.035 | 0.262 | -0.211 | 0.266 | 0.189 | **0.025** | 0.862 | 0.186 | 0.291 | 0.180 |
| COL6A3 | 0.025 | -0.260 | 0.138 | 0.216 | -.397* | 0.237 | 0.901 | 0.191 | 0.494 | 0.280 | **0.040** | 0.234 |
| COL7A1 | 0.203 | -.460* | .455* | 0.338 | -0.234 | 0.282 | 0.310 | **0.016** | **0.017** | 0.085 | 0.240 | 0.154 |
| COL8A1 | 0.044 | -0.183 | -0.085 | 0.039 | -0.234 | 0.160 | 0.827 | 0.360 | 0.675 | 0.846 | 0.240 | 0.425 |
| CTGF | -0.015 | -0.125 | -0.161 | 0.034 | -0.124 | 0.015 | 0.942 | 0.536 | 0.422 | 0.868 | 0.537 | 0.942 |
| CX3CR1 | -0.355 | 0.142 | -0.239 | -.544** | 0.121 | -0.366 | 0.069 | 0.479 | 0.230 | **0.003** | 0.548 | 0.060 |
| DLK1 | 0.297 | -.458* | 0.110 | 0.041 | -0.201 | 0.370 | 0.132 | **0.016** | 0.585 | 0.838 | 0.315 | 0.057 |
| DPP4 | -.536** | 0.291 | -0.060 | -0.210 | -0.073 | -.425* | **0.004** | 0.140 | 0.765 | 0.294 | 0.719 | **0.027** |
| DRD3 | -0.198 | 0.163 | 0.035 | -0.253 | 0.243 | -0.288 | 0.323 | 0.417 | 0.863 | 0.203 | 0.222 | 0.145 |
| ECE1 | -0.066 | -0.189 | 0.107 | 0.288 | -0.156 | 0.054 | 0.742 | 0.346 | 0.596 | 0.145 | 0.437 | 0.788 |
| END1 | -0.213 | 0.309 | 0.020 | .414* | -0.253 | -0.066 | 0.286 | 0.116 | 0.920 | **0.032** | 0.203 | 0.744 |
| FCGR3A | -0.198 | 0.124 | 0.156 | 0.266 | -.429* | -0.012 | 0.323 | 0.537 | 0.439 | 0.180 | **0.026** | 0.953 |
| FKBP5 | 0.010 | 0.075 | 0.000 | 0.257 | -0.178 | 0.100 | 0.959 | 0.708 | 1.000 | 0.195 | 0.374 | 0.620 |
| FLT1 | -0.063 | -0.090 | 0.099 | 0.364 | -.470* | 0.160 | 0.756 | 0.657 | 0.623 | 0.062 | **0.013** | 0.425 |
| FLT4 | .405* | -0.302 | 0.262 | 0.268 | -0.087 | .457* | **0.036** | 0.125 | 0.186 | 0.176 | 0.666 | **0.016** |
| FN1 | -0.068 | 0.016 | -0.076 | 0.305 | -0.184 | 0.019 | 0.737 | 0.936 | 0.706 | 0.121 | 0.358 | 0.926 |
| GLRA2 | -.542** | .390* | -0.037 | -0.380 | 0.268 | -.646** | **0.003** | **0.044** | 0.855 | 0.051 | 0.177 | **0.000** |
| HAVCR2 | -0.276 | -0.079 | -0.205 | -0.055 | -0.143 | -0.181 | 0.164 | 0.696 | 0.306 | 0.787 | 0.478 | 0.365 |
| ICAM1 | 0.222 | -0.181 | 0.107 | .621** | -.421* | 0.355 | 0.265 | 0.365 | 0.594 | **0.001** | **0.029** | 0.070 |
| IFITM3 | 0.005 | -0.068 | 0.198 | .451* | -0.321 | 0.152 | 0.979 | 0.738 | 0.322 | **0.018** | 0.103 | 0.449 |
| IL1R1 | -0.010 | -0.034 | 0.208 | .396* | -.483* | 0.245 | 0.960 | 0.867 | 0.298 | **0.041** | **0.011** | 0.218 |
| ITGA5 | -0.080 | -0.174 | -0.038 | 0.366 | -.391* | 0.091 | 0.692 | 0.387 | 0.852 | 0.061 | **0.044** | 0.652 |
| ITGA8 | 0.379 | -0.322 | -0.019 | 0.187 | 0.025 | 0.374 | 0.051 | 0.102 | 0.924 | 0.350 | 0.901 | 0.055 |
| ITGA9 | .465* | -0.146 | 0.290 | 0.231 | -0.176 | .541** | **0.015** | 0.466 | 0.142 | 0.246 | 0.380 | **0.004** |
| ITGB4 | 0.006 | -.404* | 0.011 | 0.173 | -0.144 | 0.056 | 0.975 | **0.036** | 0.958 | 0.388 | 0.475 | 0.782 |
| ITGB8 | -0.201 | -0.249 | -0.225 | -0.003 | -0.116 | -0.121 | 0.315 | 0.211 | 0.259 | 0.987 | 0.564 | 0.549 |
| LAMA2 | -0.155 | -0.222 | -0.345 | -0.055 | 0.060 | -0.158 | 0.440 | 0.266 | 0.078 | 0.784 | 0.766 | 0.431 |
| LAMA5 | .419* | -.688** | .433* | 0.258 | -0.127 | .454* | **0.030** | **0.000** | **0.024** | 0.194 | 0.527 | **0.017** |
| MYH11 | 0.261 | -.431* | -0.008 | 0.188 | -0.044 | 0.259 | 0.188 | **0.025** | 0.967 | 0.348 | 0.828 | 0.192 |
| MYL9 | 0.355 | -0.327 | -0.050 | 0.264 | -0.160 | .425* | 0.069 | 0.096 | 0.804 | 0.183 | 0.424 | **0.027** |
| P2RY12 | -.387* | 0.186 | -0.356 | -.564** | 0.220 | -.459* | **0.046** | 0.353 | 0.069 | **0.002** | 0.270 | **0.016** |
| P2RY13 | -.424* | 0.147 | -.386* | -.573** | 0.188 | -.473* | **0.027** | 0.465 | **0.047** | **0.002** | 0.346 | **0.013** |
| PDYN | -0.255 | 0.308 | -0.103 | -.581** | 0.308 | -0.376 | 0.198 | 0.118 | 0.608 | **0.001** | 0.118 | 0.053 |
| PENK | -.474* | 0.234 | 0.052 | -.411* | 0.200 | -.534** | **0.013** | 0.240 | 0.797 | **0.033** | 0.317 | **0.004** |
| S100A4 | -0.244 | -0.243 | -0.189 | -0.246 | -0.289 | -0.107 | 0.220 | 0.222 | 0.344 | 0.216 | 0.144 | 0.596 |
| SERPINA1 | -0.105 | 0.099 | 0.096 | .417* | -.436* | 0.066 | 0.603 | 0.622 | 0.635 | **0.031** | **0.023** | 0.745 |
| SERPINA3 | -0.098 | 0.005 | 0.072 | .491** | -.408* | 0.090 | 0.628 | 0.981 | 0.723 | **0.009** | **0.035** | 0.654 |
| SHISA2 | -.612** | 0.175 | -0.248 | -.463* | 0.144 | -.699** | **0.001** | 0.383 | 0.213 | **0.015** | 0.474 | **0.000** |
| SLC13A5 | -0.016 | -0.180 | 0.002 | -0.039 | .466* | -0.260 | 0.935 | 0.368 | 0.992 | 0.846 | **0.014** | 0.189 |
| SOCS3 | 0.159 | 0.032 | 0.176 | .572** | -.497** | 0.317 | 0.427 | 0.873 | 0.379 | **0.002** | **0.008** | 0.107 |
| TGFB2 | -0.162 | -0.175 | -0.228 | -0.001 | -0.148 | -0.130 | 0.419 | 0.384 | 0.252 | 0.995 | 0.460 | 0.519 |
| TGM2 | 0.169 | -0.189 | 0.179 | .527** | -.383* | 0.335 | 0.400 | 0.344 | 0.371 | **0.005** | **0.049** | 0.088 |
| THY1 | -0.027 | 0.176 | 0.238 | -0.041 | 0.262 | -0.074 | 0.894 | 0.379 | 0.231 | 0.841 | 0.187 | 0.712 |
| TIMP1 | 0.060 | -0.205 | 0.105 | .437* | -.471* | 0.237 | 0.766 | 0.306 | 0.601 | **0.023** | **0.013** | 0.235 |
| TMEM119 | -0.286 | 0.104 | -0.292 | -.488** | 0.133 | -0.330 | 0.148 | 0.607 | 0.139 | **0.010** | 0.510 | 0.093 |
| TTR | -0.291 | 0.159 | -0.055 | -.398* | 0.030 | -0.352 | 0.141 | 0.428 | 0.785 | **0.040** | 0.881 | 0.071 |
| VEGFA | -0.042 | -0.237 | 0.218 | 0.181 | -0.372 | 0.121 | 0.835 | 0.234 | 0.274 | 0.366 | 0.056 | 0.547 |
| VIM | 0.041 | -0.290 | -0.110 | 0.206 | -0.228 | 0.135 | 0.839 | 0.142 | 0.584 | 0.304 | 0.252 | 0.502 |

*Note:* Sample size for all comparisons is n=27. Genes are listed aphabetically.
