# Supplementary material for: RNA-sequencing suggests extracellular matrix and vasculature dysregulation could impair neurogenesis in schizophrenia cases with elevated inflammation
Source: Schizophrenia (Heidelb). 2024 May 4;10(1):50. doi: 10.1038/s41537-024-00466-0 (PMC11069512; doi:10.1038/s41537-024-00466-0)
Supplement: Supplementary file 2 — Appendix Table 2 [file 41537_2024_466_MOESM2_ESM.docx]

**Appendix Table A2: Differentially expressed genes between the inflammatory subgroups of schizophrenia after multiple testing correction, with a False Discovery Rate (FDR) adjusted p value (q) < 0.05. Genes are listed in order of log fold change (FC).**

| **GeneID** | **logFC** | **PValue** | **FDR q** | **GeneName** |
| --- | --- | --- | --- | --- |
| ENSG00000196136 | 2.48594747 | 1.53E-104 | 2.00E-100 | SERPINA3 |
| ENSG00000273259 | 2.43382116 | 2.47E-101 | 1.61E-97 | SERPINA3 |
| ENSG00000177575 | 2.0654904 | 2.82E-74 | 1.23E-70 | CD163 |
| ENSG00000184557 | 2.04287912 | 6.12E-70 | 2.00E-66 | SOCS3 |
| ENSG00000064886 | 1.88492786 | 3.52E-61 | 9.20E-58 | CHI3L2 |
| ENSG00000135245 | 1.85548617 | 1.45E-60 | 3.15E-57 | HILPDA |
| ENSG00000133048 | 1.74902534 | 4.63E-55 | 8.63E-52 | CHI3L1 |
| ENSG00000165507 | 1.71033737 | 1.19E-52 | 1.94E-49 | C10orf10 |
| ENSG00000148926 | 1.643097 | 1.80E-47 | 2.62E-44 | ADM |
| ENSG00000244734 | 1.49453423 | 4.32E-42 | 5.64E-39 | HBB |
| ENSG00000155659 | 1.41645693 | 2.22E-36 | 2.64E-33 | VSIG4 |
| ENSG00000145623 | 1.3495054 | 4.17E-34 | 4.54E-31 | OSMR |
| ENSG00000187498 | 1.32220166 | 1.27E-32 | 1.27E-29 | COL4A1 |
| ENSG00000167772 | 1.30777426 | 1.66E-31 | 1.45E-28 | ANGPTL4 |
| ENSG00000096060 | 1.29570909 | 3.65E-32 | 3.40E-29 | FKBP5 |
| ENSG00000078401 | 1.27670565 | 9.79E-30 | 7.98E-27 | EDN1 |
| ENSG00000198959 | 1.24014914 | 1.91E-29 | 1.47E-26 | TGM2 |
| ENSG00000012223 | 1.22733044 | 4.41E-29 | 3.20E-26 | LTF |
| ENSG00000197249 | 1.19319139 | 1.27E-25 | 7.89E-23 | SERPINA1 |
| ENSG00000125810 | 1.19121914 | 1.49E-26 | 1.03E-23 | CD93 |
| ENSG00000188536 | 1.16787635 | 1.22E-25 | 7.89E-23 | HBA2 |
| ENSG00000065911 | 1.16380004 | 4.98E-25 | 2.95E-22 | MTHFD2 |
| ENSG00000185201 | 1.13180152 | 5.12E-24 | 2.78E-21 | IFITM2 |
| ENSG00000104635 | 1.12950608 | 5.21E-25 | 2.96E-22 | SLC39A14 |
| ENSG00000168209 | 1.09913045 | 8.50E-24 | 4.43E-21 | DDIT4 |
| ENSG00000142089 | 1.09728602 | 1.18E-23 | 5.90E-21 | IFITM3 |
| ENSG00000203747 | 1.09643747 | 2.30E-23 | 1.11E-20 | FCGR3A |
| ENSG00000225630 | 1.09269392 | 1.43E-22 | 6.65E-20 | MTND2P28 |
| ENSG00000111341 | 1.09263914 | 9.73E-22 | 4.09E-19 | MGP |
| ENSG00000077238 | 1.06350889 | 1.26E-21 | 5.12E-19 | IL4R |
| ENSG00000187193 | 1.06304042 | 3.05E-22 | 1.32E-19 | MT1X |
| ENSG00000141469 | 1.05907523 | 1.96E-22 | 8.83E-20 | SLC14A1 |
| ENSG00000162645 | 1.04379107 | 1.12E-20 | 4.30E-18 | GBP2 |
| ENSG00000115594 | 1.04238904 | 9.15E-21 | 3.62E-18 | IL1R1 |
| ENSG00000149591 | 1.01778104 | 1.48E-20 | 5.52E-18 | TAGLN |
| ENSG00000137801 | 1.01243367 | 3.95E-20 | 1.43E-17 | THBS1 |
| ENSG00000112936 | 0.98412665 | 1.26E-18 | 4.21E-16 | C7 |
| ENSG00000189223 | 0.97062379 | 1.67E-18 | 5.46E-16 | PAX8-AS1 |
| ENSG00000124762 | 0.96276 | 2.39E-18 | 7.59E-16 | CDKN1A |
| ENSG00000137507 | 0.95593511 | 3.11E-17 | 8.64E-15 | LRRC32 |
| ENSG00000213088 | 0.94937924 | 4.96E-17 | 1.35E-14 | ACKR1 |
| ENSG00000047457 | 0.94372654 | 3.14E-18 | 9.75E-16 | CP |
| ENSG00000112715 | 0.9379342 | 6.63E-18 | 2.01E-15 | VEGFA |
| ENSG00000159403 | 0.93646188 | 9.79E-18 | 2.90E-15 | C1R |
| ENSG00000277918 | 0.93558009 | 1.34E-17 | 3.89E-15 | U1 |
| ENSG00000185885 | 0.93323821 | 2.41E-17 | 6.84E-15 | IFITM1 |
| ENSG00000128016 | 0.92927076 | 5.22E-17 | 1.36E-14 | ZFP36 |
| ENSG00000197142 | 0.92763708 | 7.10E-17 | 1.78E-14 | ACSL5 |
| ENSG00000134871 | 0.9243988 | 6.12E-17 | 1.56E-14 | COL4A2 |
| ENSG00000060138 | 0.9128269 | 8.54E-17 | 2.06E-14 | YBX3 |
| ENSG00000110799 | 0.90880209 | 5.16E-17 | 1.36E-14 | VWF |
| ENSG00000134531 | 0.90768904 | 7.83E-17 | 1.93E-14 | EMP1 |
| ENSG00000143226 | 0.90443926 | 2.43E-16 | 5.66E-14 | FCGR2A |
| ENSG00000135426 | 0.89772839 | 2.22E-16 | 5.27E-14 | TESPA1 |
| ENSG00000107796 | 0.88029793 | 8.57E-16 | 1.96E-13 | ACTA2 |
| ENSG00000247081 | 0.88004885 | 3.11E-15 | 6.77E-13 | BAALC-AS1 |
| ENSG00000163359 | 0.87637031 | 1.04E-14 | 2.16E-12 | COL6A3 |
| ENSG00000149257 | 0.87615551 | 1.92E-15 | 4.32E-13 | SERPINH1 |
| ENSG00000105639 | 0.87162604 | 1.41E-14 | 2.88E-12 | JAK3 |
| ENSG00000099998 | 0.86872859 | 2.27E-14 | 4.48E-12 | GGT5 |
| ENSG00000026508 | 0.85799362 | 2.17E-15 | 4.81E-13 | CD44 |
| ENSG00000122862 | 0.85714356 | 3.98E-15 | 8.51E-13 | SRGN |
| ENSG00000124491 | 0.85275377 | 7.16E-15 | 1.51E-12 | F13A1 |
| ENSG00000037280 | 0.84934255 | 6.58E-14 | 1.25E-11 | FLT4 |
| ENSG00000137193 | 0.83756855 | 5.28E-14 | 1.01E-11 | PIM1 |
| ENSG00000151929 | 0.83490407 | 2.23E-14 | 4.48E-12 | BAG3 |
| ENSG00000103196 | 0.83216803 | 9.09E-14 | 1.69E-11 | CRISPLD2 |
| ENSG00000180914 | 0.82907434 | 3.08E-13 | 5.22E-11 | OXTR |
| ENSG00000235750 | 0.82543805 | 2.21E-13 | 3.87E-11 | KIAA0040 |
| ENSG00000205364 | 0.81845833 | 1.64E-13 | 2.93E-11 | MT1M |
| ENSG00000173369 | 0.81073511 | 1.01E-13 | 1.86E-11 | C1QB |
| ENSG00000141753 | 0.8082215 | 9.00E-13 | 1.45E-10 | IGFBP4 |
| ENSG00000243244 | 0.8056034 | 2.75E-13 | 4.72E-11 | STON1 |
| ENSG00000000971 | 0.803748 | 1.62E-13 | 2.93E-11 | CFH |
| ENSG00000169908 | 0.8011598 | 3.54E-13 | 5.92E-11 | TM4SF1 |
| ENSG00000133800 | 0.80080881 | 4.43E-13 | 7.31E-11 | LYVE1 |
| ENSG00000182326 | 0.79722599 | 2.23E-13 | 3.87E-11 | C1S |
| ENSG00000119681 | 0.77857985 | 2.81E-12 | 4.17E-10 | LTBP2 |
| ENSG00000196169 | 0.77514855 | 1.98E-12 | 3.04E-10 | KIF19 |
| ENSG00000102265 | 0.77388395 | 3.04E-12 | 4.46E-10 | TIMP1 |
| ENSG00000099860 | 0.77316993 | 1.40E-12 | 2.21E-10 | GADD45B |
| ENSG00000162772 | 0.77113859 | 2.14E-12 | 3.25E-10 | ATF3 |
| ENSG00000144810 | 0.76775545 | 1.61E-12 | 2.50E-10 | COL8A1 |
| ENSG00000149131 | 0.76588013 | 2.36E-12 | 3.54E-10 | SERPING1 |
| ENSG00000109906 | 0.75958788 | 3.27E-12 | 4.75E-10 | ZBTB16 |
| ENSG00000239704 | 0.75827106 | 9.10E-12 | 1.29E-09 | CDRT4 |
| ENSG00000243649 | 0.7557932 | 1.75E-11 | 2.43E-09 | CFB |
| ENSG00000167553 | 0.74089946 | 4.36E-11 | 5.74E-09 | TUBA1C |
| ENSG00000261371 | 0.74040044 | 9.60E-12 | 1.35E-09 | PECAM1 |
| ENSG00000139178 | 0.73590293 | 2.66E-11 | 3.61E-09 | C1RL |
| ENSG00000224389 | 0.73264612 | 2.03E-11 | 2.79E-09 | C4B |
| ENSG00000163431 | 0.7319434 | 4.27E-11 | 5.68E-09 | LMOD1 |
| ENSG00000115107 | 0.72613723 | 1.17E-10 | 1.47E-08 | STEAP3 |
| ENSG00000168874 | 0.72541948 | 9.27E-11 | 1.19E-08 | ATOH8 |
| ENSG00000090339 | 0.72336506 | 1.21E-10 | 1.50E-08 | ICAM1 |
| ENSG00000067182 | 0.7197251 | 5.59E-11 | 7.30E-09 | TNFRSF1A |
| ENSG00000090104 | 0.71628942 | 3.42E-11 | 4.59E-09 | RGS1 |
| ENSG00000144908 | 0.70669593 | 6.29E-11 | 8.12E-09 | ALDH1L1 |
| ENSG00000197620 | 0.70033465 | 2.93E-10 | 3.39E-08 | CXorf40A |
| ENSG00000275395 | 0.69995341 | 2.32E-10 | 2.80E-08 | FCGBP |
| ENSG00000205403 | 0.6989345 | 4.50E-10 | 5.08E-08 | CFI |
| ENSG00000116717 | 0.69706562 | 3.42E-10 | 3.91E-08 | GADD45A |
| ENSG00000245532 | 0.696075 | 1.01E-10 | 1.28E-08 | NEAT1 |
| ENSG00000168542 | 0.69491178 | 6.34E-10 | 6.95E-08 | COL3A1 |
| ENSG00000152049 | 0.69238538 | 9.63E-10 | 1.03E-07 | KCNE4 |
| ENSG00000135842 | 0.692237 | 4.72E-10 | 5.26E-08 | FAM129A |
| ENSG00000100504 | 0.68711597 | 4.52E-10 | 5.08E-08 | PYGL |
| ENSG00000102755 | 0.68445292 | 2.34E-10 | 2.80E-08 | FLT1 |
| ENSG00000113916 | 0.68307999 | 2.80E-10 | 3.30E-08 | BCL6 |
| ENSG00000117525 | 0.68276998 | 2.54E-10 | 3.02E-08 | F3 |
| ENSG00000108932 | 0.68058846 | 9.39E-10 | 1.01E-07 | SLC16A6 |
| ENSG00000120708 | 0.68005948 | 9.27E-10 | 1.01E-07 | TGFBI |
| ENSG00000147234 | 0.66952868 | 1.79E-09 | 1.80E-07 | FRMPD3 |
| ENSG00000131378 | 0.66569263 | 1.25E-09 | 1.31E-07 | RFTN1 |
| ENSG00000170458 | 0.66515588 | 1.47E-09 | 1.52E-07 | CD14 |
| ENSG00000188313 | 0.66470115 | 1.37E-09 | 1.43E-07 | PLSCR1 |
| ENSG00000138061 | 0.65773661 | 1.51E-09 | 1.55E-07 | CYP1B1 |
| ENSG00000056998 | 0.65625798 | 1.54E-09 | 1.57E-07 | GYG2 |
| ENSG00000137462 | 0.65574728 | 3.42E-09 | 3.35E-07 | TLR2 |
| ENSG00000108551 | 0.65523238 | 1.65E-09 | 1.67E-07 | RASD1 |
| ENSG00000206538 | 0.65467839 | 4.17E-09 | 4.06E-07 | VGLL3 |
| ENSG00000064932 | 0.65114847 | 1.04E-08 | 9.33E-07 | SBNO2 |
| ENSG00000139567 | 0.64805569 | 5.93E-09 | 5.69E-07 | ACVRL1 |
| ENSG00000132470 | 0.64594648 | 2.26E-09 | 2.25E-07 | ITGB4 |
| ENSG00000113369 | 0.64051181 | 3.00E-09 | 2.96E-07 | ARRDC3 |
| ENSG00000187905 | 0.6403134 | 9.41E-09 | 8.58E-07 | LRRC74B |
| ENSG00000102007 | 0.63700151 | 2.23E-08 | 1.89E-06 | PLP2 |
| ENSG00000105339 | 0.6361791 | 4.65E-09 | 4.50E-07 | DENND3 |
| ENSG00000106211 | 0.62972867 | 7.78E-09 | 7.35E-07 | HSPB1 |
| ENSG00000115457 | 0.62821399 | 2.50E-08 | 2.08E-06 | IGFBP2 |
| ENSG00000102362 | 0.62816989 | 7.83E-09 | 7.35E-07 | SYTL4 |
| ENSG00000168386 | 0.62788957 | 2.97E-08 | 2.41E-06 | FILIP1L |
| ENSG00000124440 | 0.62155109 | 1.68E-08 | 1.45E-06 | HIF3A |
| ENSG00000265972 | 0.6188818 | 8.79E-09 | 8.14E-07 | TXNIP |
| ENSG00000152256 | 0.61837364 | 1.37E-08 | 1.20E-06 | PDK1 |
| ENSG00000135926 | 0.61776738 | 1.05E-08 | 9.39E-07 | TMBIM1 |
| ENSG00000180353 | 0.61425751 | 2.39E-08 | 2.00E-06 | HCLS1 |
| ENSG00000118785 | 0.61007059 | 1.44E-08 | 1.25E-06 | SPP1 |
| ENSG00000159399 | 0.60926087 | 2.62E-08 | 2.17E-06 | HK2 |
| ENSG00000072682 | 0.6043316 | 4.50E-08 | 3.51E-06 | P4HA2 |
| ENSG00000131386 | 0.60409041 | 2.02E-08 | 1.72E-06 | GALNT15 |
| ENSG00000257337 | 0.60387659 | 5.23E-08 | 4.04E-06 | RP11-983P16.4 |
| ENSG00000159189 | 0.60141329 | 3.70E-08 | 2.96E-06 | C1QC |
| ENSG00000158710 | 0.59999781 | 3.09E-08 | 2.49E-06 | TAGLN2 |
| ENSG00000179776 | 0.59789064 | 5.91E-08 | 4.51E-06 | CDH5 |
| ENSG00000169507 | 0.59389708 | 1.50E-07 | 1.11E-05 | SLC38A11 |
| ENSG00000161638 | 0.58910815 | 1.38E-07 | 1.03E-05 | ITGA5 |
| ENSG00000178878 | 0.58657259 | 5.68E-08 | 4.36E-06 | APOLD1 |
| ENSG00000182541 | 0.58214204 | 7.62E-08 | 5.78E-06 | LIMK2 |
| ENSG00000170074 | 0.57101702 | 3.51E-07 | 2.49E-05 | FAM153A |
| ENSG00000214425 | 0.56911119 | 2.33E-07 | 1.69E-05 | LRRC37A4P |
| ENSG00000182492 | 0.56818446 | 2.31E-07 | 1.68E-05 | BGN |
| ENSG00000221963 | 0.56582037 | 1.76E-07 | 1.29E-05 | APOL6 |
| ENSG00000164761 | 0.56413745 | 4.61E-07 | 3.16E-05 | TNFRSF11B |
| ENSG00000162598 | 0.56363738 | 2.07E-07 | 1.52E-05 | C1orf87 |
| ENSG00000241684 | 0.56042133 | 7.45E-07 | 4.89E-05 | ADAMTS9-AS2 |
| ENSG00000160691 | 0.55917183 | 3.16E-07 | 2.25E-05 | SHC1 |
| ENSG00000164104 | 0.55668386 | 2.83E-07 | 2.03E-05 | HMGB2 |
| ENSG00000134817 | 0.55570125 | 2.48E-07 | 1.79E-05 | APLNR |
| ENSG00000117228 | 0.55444816 | 3.75E-07 | 2.65E-05 | GBP1 |
| ENSG00000112312 | 0.55373 | 6.24E-07 | 4.13E-05 | GMNN |
| ENSG00000173281 | 0.55347921 | 5.95E-07 | 3.96E-05 | PPP1R3B |
| ENSG00000142798 | 0.55172743 | 7.95E-07 | 5.13E-05 | HSPG2 |
| ENSG00000154262 | 0.55068456 | 4.22E-07 | 2.96E-05 | ABCA6 |
| ENSG00000183943 | 0.5504056 | 4.31E-07 | 3.00E-05 | PRKX |
| ENSG00000106565 | 0.54919717 | 5.47E-07 | 3.68E-05 | TMEM176B |
| ENSG00000111859 | 0.54845874 | 4.73E-07 | 3.23E-05 | NEDD9 |
| ENSG00000138119 | 0.54841496 | 4.32E-07 | 3.00E-05 | MYOF |
| ENSG00000125089 | 0.54635453 | 1.26E-06 | 7.89E-05 | SH3TC1 |
| ENSG00000168077 | 0.54566841 | 4.44E-07 | 3.07E-05 | SCARA3 |
| ENSG00000214688 | 0.54235883 | 5.61E-07 | 3.76E-05 | C10orf105 |
| ENSG00000108821 | 0.54085583 | 1.63E-06 | 9.96E-05 | COL1A1 |
| ENSG00000162618 | 0.54064008 | 1.79E-06 | 0.00010823 | ELTD1 |
| ENSG00000165030 | 0.54026763 | 1.02E-06 | 6.41E-05 | NFIL3 |
| ENSG00000112096 | 0.53913643 | 5.22E-07 | 3.55E-05 | SOD2 |
| ENSG00000130052 | 0.53804222 | 1.89E-06 | 0.00011341 | STARD8 |
| ENSG00000125148 | 0.53757545 | 6.82E-07 | 4.50E-05 | MT2A |
| ENSG00000122679 | 0.53749453 | 1.77E-06 | 0.00010743 | RAMP3 |
| ENSG00000046604 | 0.53655735 | 2.13E-06 | 0.00012491 | DSG2 |
| ENSG00000231768 | 0.53526853 | 9.52E-07 | 6.03E-05 | LINC01354 |
| ENSG00000137767 | 0.53103388 | 2.93E-06 | 0.0001685 | SQRDL |
| ENSG00000072952 | 0.52885992 | 8.95E-07 | 5.70E-05 | MRVI1 |
| ENSG00000028137 | 0.52629271 | 2.01E-06 | 0.00011858 | TNFRSF1B |
| ENSG00000118898 | 0.5239535 | 1.89E-06 | 0.00011341 | PPL |
| ENSG00000086544 | 0.52226205 | 2.52E-06 | 0.00014706 | ITPKC |
| ENSG00000106624 | 0.51553131 | 1.71E-06 | 0.00010425 | AEBP1 |
| ENSG00000004799 | 0.5130659 | 1.91E-06 | 0.000114 | PDK4 |
| ENSG00000230062 | 0.51249882 | 3.17E-06 | 0.00018082 | ANKRD66 |
| ENSG00000008394 | 0.51115743 | 2.30E-06 | 0.00013484 | MGST1 |
| ENSG00000181019 | 0.50768915 | 2.78E-06 | 0.00016125 | NQO1 |
| ENSG00000171903 | 0.50650446 | 3.93E-06 | 0.00022121 | CYP4F11 |
| ENSG00000025708 | 0.50352461 | 6.77E-06 | 0.00035471 | TYMP |
| ENSG00000186529 | 0.50229171 | 4.18E-06 | 0.0002333 | CYP4F3 |
| ENSG00000117519 | 0.50072488 | 3.16E-06 | 0.00018071 | CNN3 |
| ENSG00000090382 | 0.49847275 | 1.13E-05 | 0.00056114 | LYZ |
| ENSG00000173926 | 0.49840415 | 7.95E-06 | 0.00041373 | MARCHF3 |
| ENSG00000187720 | 0.49810188 | 5.16E-06 | 0.00028284 | THSD4 |
| ENSG00000157554 | 0.497522 | 1.14E-05 | 0.00056409 | ERG |
| ENSG00000166682 | 0.49657267 | 6.43E-06 | 0.00034261 | TMPRSS5 |
| ENSG00000101335 | 0.49606741 | 1.02E-05 | 0.00051738 | MYL9 |
| ENSG00000204388 | 0.49540675 | 4.18E-06 | 0.0002333 | HSPA1B |
| ENSG00000118523 | 0.4947587 | 5.61E-06 | 0.00030497 | CTGF |
| ENSG00000070404 | 0.49253808 | 1.23E-05 | 0.00060079 | FSTL3 |
| ENSG00000204396 | 0.49186974 | 1.32E-05 | 0.00063839 | VWA7 |
| ENSG00000153789 | 0.491148 | 8.41E-06 | 0.00043563 | FAM92B |
| ENSG00000213719 | 0.49107229 | 6.26E-06 | 0.00033467 | CLIC1 |
| ENSG00000134285 | 0.49027837 | 6.69E-06 | 0.00035344 | FKBP11 |
| ENSG00000206199 | 0.490141 | 1.07E-05 | 0.00053454 | ANKUB1 |
| ENSG00000140853 | 0.48984208 | 1.15E-05 | 0.00056409 | NLRC5 |
| ENSG00000105835 | 0.48898583 | 5.65E-06 | 0.00030608 | NAMPT |
| ENSG00000198734 | 0.48873993 | 1.11E-05 | 0.00055362 | F5 |
| ENSG00000150961 | 0.48557413 | 1.40E-05 | 0.00066965 | SEC24D |
| ENSG00000024422 | 0.48483915 | 2.07E-05 | 0.00093521 | EHD2 |
| ENSG00000088280 | 0.48422126 | 8.53E-06 | 0.00043978 | ASAP3 |
| ENSG00000132793 | 0.48402332 | 2.20E-05 | 0.00098964 | LPIN3 |
| ENSG00000240583 | 0.48355991 | 6.73E-06 | 0.0003539 | AQP1 |
| ENSG00000136010 | 0.48235198 | 1.36E-05 | 0.00065259 | ALDH1L2 |
| ENSG00000261617 | 0.48212545 | 1.66E-05 | 0.00078215 | RP11-243A14.1 |
| ENSG00000159212 | 0.48114307 | 1.42E-05 | 0.00067894 | CLIC6 |
| ENSG00000147872 | 0.48020779 | 1.25E-05 | 0.00060873 | PLIN2 |
| ENSG00000162493 | 0.4791658 | 1.16E-05 | 0.00056661 | PDPN |
| ENSG00000077943 | 0.47875894 | 1.96E-05 | 0.0008959 | ITGA8 |
| ENSG00000117643 | 0.47794743 | 1.04E-05 | 0.00052727 | MAN1C1 |
| ENSG00000120262 | 0.47617851 | 1.06E-05 | 0.00053454 | CCDC170 |
| ENSG00000203814 | 0.47216785 | 1.50E-05 | 0.00070951 | HIST2H2BF |
| ENSG00000122884 | 0.47212457 | 1.23E-05 | 0.00060079 | P4HA1 |
| ENSG00000281508 | 0.4712064 | 1.14E-05 | 0.00056409 | CDR1-AS |
| ENSG00000203734 | 0.46811262 | 2.05E-05 | 0.00093409 | ECT2L |
| ENSG00000133392 | 0.46753341 | 2.00E-05 | 0.00091377 | MYH11 |
| ENSG00000243910 | 0.46625051 | 3.49E-05 | 0.00149779 | TUBA4B |
| ENSG00000130702 | 0.46624211 | 2.80E-05 | 0.0012336 | LAMA5 |
| ENSG00000133107 | 0.46604733 | 3.78E-05 | 0.00160725 | TRPC4 |
| ENSG00000104812 | 0.46556967 | 2.07E-05 | 0.00093521 | GYS1 |
| ENSG00000173221 | 0.4649318 | 2.91E-05 | 0.00127247 | GLRX |
| ENSG00000107249 | 0.46416249 | 1.76E-05 | 0.00081942 | GLIS3 |
| ENSG00000140795 | 0.46361973 | 1.65E-05 | 0.000778 | MYLK3 |
| ENSG00000200795 | 0.46265526 | 1.79E-05 | 0.0008262 | RNU4-1 |
| ENSG00000189058 | 0.46218973 | 1.74E-05 | 0.00081333 | APOD |
| ENSG00000188404 | 0.45956672 | 4.17E-05 | 0.00172747 | SELL |
| ENSG00000138772 | 0.45851148 | 4.60E-05 | 0.00188329 | ANXA3 |
| ENSG00000030304 | 0.45836006 | 3.19E-05 | 0.0013868 | MUSK |
| ENSG00000164181 | 0.45629001 | 2.56E-05 | 0.00114514 | ELOVL7 |
| ENSG00000026297 | 0.45580129 | 2.62E-05 | 0.00116778 | RNASET2 |
| ENSG00000002933 | 0.45293312 | 5.58E-05 | 0.00222523 | TMEM176A |
| ENSG00000105479 | 0.45136309 | 3.63E-05 | 0.00154702 | CCDC114 |
| ENSG00000215187 | 0.45115982 | 4.59E-05 | 0.00188329 | FAM166B |
| ENSG00000133115 | 0.45113452 | 3.49E-05 | 0.00149779 | STOML3 |
| ENSG00000091879 | 0.45043638 | 6.94E-05 | 0.00263981 | ANGPT2 |
| ENSG00000157578 | 0.45031364 | 5.25E-05 | 0.00210603 | LCA5L |
| ENSG00000026025 | 0.45010039 | 2.75E-05 | 0.00121524 | VIM |
| ENSG00000182718 | 0.44922676 | 3.03E-05 | 0.00132333 | ANXA2 |
| ENSG00000183688 | 0.44866227 | 5.64E-05 | 0.00224314 | FAM101B |
| ENSG00000172216 | 0.44852472 | 6.03E-05 | 0.00234729 | CEBPB |
| ENSG00000181291 | 0.44847017 | 6.63E-05 | 0.00254485 | TMEM132E |
| ENSG00000249307 | 0.44741736 | 4.83E-05 | 0.00196849 | LINC01088 |
| ENSG00000107968 | 0.4463094 | 7.61E-05 | 0.00286328 | MAP3K8 |
| ENSG00000125733 | 0.44413426 | 8.69E-05 | 0.00315205 | TRIP10 |
| ENSG00000136918 | 0.44365994 | 7.65E-05 | 0.00286739 | WDR38 |
| ENSG00000169902 | 0.44360163 | 4.13E-05 | 0.00172209 | TPST1 |
| ENSG00000129667 | 0.44263116 | 6.92E-05 | 0.00263981 | RHBDF2 |
| ENSG00000182240 | 0.44253347 | 8.47E-05 | 0.00310744 | BACE2 |
| ENSG00000021355 | 0.44153842 | 5.30E-05 | 0.00212263 | SERPINB1 |
| ENSG00000013364 | 0.44148441 | 4.94E-05 | 0.00200836 | MVP |
| ENSG00000198682 | 0.44112659 | 7.86E-05 | 0.00292941 | PAPSS2 |
| ENSG00000168309 | 0.44014051 | 4.05E-05 | 0.00170077 | FAM107A |
| ENSG00000135046 | 0.44013331 | 4.21E-05 | 0.00173986 | ANXA1 |
| ENSG00000089159 | 0.43993649 | 6.02E-05 | 0.00234729 | PXN |
| ENSG00000126709 | 0.43951075 | 5.16E-05 | 0.00207942 | IFI6 |
| ENSG00000122986 | 0.43866043 | 7.59E-05 | 0.00286328 | HVCN1 |
| ENSG00000165806 | 0.43794242 | 7.24E-05 | 0.00274649 | CASP7 |
| ENSG00000111783 | 0.43728752 | 5.04E-05 | 0.00204201 | RFX4 |
| ENSG00000279133 | 0.43700854 | 0.00012211 | 0.00421496 | RP11-342K2.1 |
| ENSG00000150687 | 0.43602977 | 6.58E-05 | 0.00253168 | PRSS23 |
| ENSG00000143545 | 0.43557449 | 8.02E-05 | 0.00297988 | RAB13 |
| ENSG00000122863 | 0.43344122 | 6.13E-05 | 0.00237993 | CHST3 |
| ENSG00000162643 | 0.43329861 | 6.68E-05 | 0.00255532 | WDR63 |
| ENSG00000232224 | 0.43260648 | 0.00010121 | 0.00360598 | LINC00202-1 |
| ENSG00000126003 | 0.42814029 | 0.00011099 | 0.0039034 | PLAGL2 |
| ENSG00000103710 | 0.42769244 | 8.17E-05 | 0.00302803 | RASL12 |
| ENSG00000197956 | 0.42726556 | 7.71E-05 | 0.00288152 | S100A6 |
| ENSG00000177337 | 0.42661447 | 0.00014561 | 0.00485897 | DLGAP1-AS1 |
| ENSG00000133321 | 0.4263446 | 0.0001027 | 0.00364135 | RARRES3 |
| ENSG00000102878 | 0.42552344 | 0.00018222 | 0.00594414 | HSF4 |
| ENSG00000122574 | 0.42473383 | 8.20E-05 | 0.0030317 | WIPF3 |
| ENSG00000168394 | 0.42459344 | 0.00010664 | 0.00377073 | TAP1 |
| ENSG00000138193 | 0.42405313 | 8.31E-05 | 0.00306125 | PLCE1 |
| ENSG00000107736 | 0.4236034 | 0.0001177 | 0.00409517 | CDH23 |
| ENSG00000162733 | 0.42290368 | 8.48E-05 | 0.00310744 | DDR2 |
| ENSG00000162496 | 0.42200622 | 0.00010142 | 0.00360598 | DHRS3 |
| ENSG00000222489 | 0.42182795 | 0.00014148 | 0.00475779 | SNORA79 |
| ENSG00000148175 | 0.419541 | 9.33E-05 | 0.00335538 | STOM |
| ENSG00000090006 | 0.41924051 | 0.00013907 | 0.00468873 | LTBP4 |
| ENSG00000187091 | 0.41787179 | 0.00012108 | 0.00419045 | PLCD1 |
| ENSG00000061273 | 0.4177592 | 0.00014379 | 0.00481418 | HDAC7 |
| ENSG00000152936 | 0.41718331 | 0.00018039 | 0.00591394 | LMNTD1 |
| ENSG00000075275 | 0.41714761 | 0.00015972 | 0.00530293 | CELSR1 |
| ENSG00000213853 | 0.4166389 | 0.00012341 | 0.00423897 | EMP2 |
| ENSG00000185681 | 0.41650771 | 0.00016878 | 0.00556129 | MORN5 |
| ENSG00000130775 | 0.41626637 | 0.00019065 | 0.00618796 | THEMIS2 |
| ENSG00000165389 | 0.41602267 | 0.00013429 | 0.00456292 | SPTSSA |
| ENSG00000114270 | 0.41557851 | 0.00018117 | 0.00592469 | COL7A1 |
| ENSG00000163638 | 0.41534726 | 0.00013046 | 0.0044445 | ADAMTS9 |
| ENSG00000233452 | 0.4146121 | 0.00020757 | 0.00667098 | STXBP5-AS1 |
| ENSG00000162551 | 0.41423425 | 0.0002698 | 0.00828321 | ALPL |
| ENSG00000143772 | 0.41310143 | 0.00011825 | 0.00410361 | ITPKB |
| ENSG00000100292 | 0.41297361 | 0.00023493 | 0.00740428 | HMOX1 |
| ENSG00000185650 | 0.41259681 | 0.00012502 | 0.0042814 | ZFP36L1 |
| ENSG00000087074 | 0.4124181 | 0.00014389 | 0.00481418 | PPP1R15A |
| ENSG00000213145 | 0.41199125 | 0.00020871 | 0.00669101 | CRIP1 |
| ENSG00000197747 | 0.41116526 | 0.00015289 | 0.00508891 | S100A10 |
| ENSG00000198088 | 0.41043588 | 0.00032908 | 0.00983421 | NUP62CL |
| ENSG00000188783 | 0.40923861 | 0.00017718 | 0.00582324 | PRELP |
| ENSG00000026036 | 0.40889621 | 0.00030306 | 0.00921757 | RTEL1-TNFRSF6B |
| ENSG00000142149 | 0.40662832 | 0.00032292 | 0.0097278 | HUNK |
| ENSG00000162148 | 0.4055072 | 0.00019701 | 0.0063786 | PPP1R32 |
| ENSG00000176046 | 0.40352105 | 0.00022803 | 0.0072218 | NUPR1 |
| ENSG00000212464 | 0.40329725 | 0.00024778 | 0.00775313 | SNORA12 |
| ENSG00000205929 | 0.4026588 | 0.000256 | 0.00795322 | C21orf62 |
| ENSG00000185666 | 0.40227444 | 0.00023171 | 0.0073206 | SYN3 |
| ENSG00000143036 | 0.40099099 | 0.00038124 | 0.01102964 | SLC44A3 |
| ENSG00000020577 | 0.40008456 | 0.00019759 | 0.00638161 | SAMD4A |
| ENSG00000141510 | 0.3990866 | 0.00041172 | 0.01173157 | TP53 |
| ENSG00000207445 | 0.39899411 | 0.00043281 | 0.01214462 | SNORD15B |
| ENSG00000173372 | 0.39852918 | 0.00025356 | 0.00789596 | C1QA |
| ENSG00000113083 | 0.39824111 | 0.0003628 | 0.01054302 | LOX |
| ENSG00000139200 | 0.39811597 | 0.00028145 | 0.00862056 | PIANP |
| ENSG00000130635 | 0.39761108 | 0.00031693 | 0.00957257 | COL5A1 |
| ENSG00000151773 | 0.3970908 | 0.00049501 | 0.01345615 | CCDC122 |
| ENSG00000136449 | 0.39625691 | 0.00039279 | 0.0112888 | MYCBPAP |
| ENSG00000144909 | 0.39605829 | 0.00023778 | 0.00747599 | OSBPL11 |
| ENSG00000125534 | 0.39506371 | 0.00043795 | 0.01221012 | PPDPF |
| ENSG00000170542 | 0.39501876 | 0.00032419 | 0.0097278 | SERPINB9 |
| ENSG00000132002 | 0.39423488 | 0.00025019 | 0.00780963 | DNAJB1 |
| ENSG00000159840 | 0.39300512 | 0.00042835 | 0.01207152 | ZYX |
| ENSG00000163453 | 0.3922327 | 0.00026888 | 0.00827437 | IGFBP7 |
| ENSG00000168610 | 0.39217448 | 0.0002623 | 0.00811019 | STAT3 |
| ENSG00000139083 | 0.39212495 | 0.00035827 | 0.01050483 | ETV6 |
| ENSG00000168658 | 0.39187641 | 0.00029575 | 0.00901633 | VWA3B |
| ENSG00000143365 | 0.3916678 | 0.00055892 | 0.01488334 | RORC |
| ENSG00000173846 | 0.39046623 | 0.00048297 | 0.01323918 | PLK3 |
| ENSG00000132256 | 0.38997397 | 0.00055237 | 0.01475183 | TRIM5 |
| ENSG00000152611 | 0.38895694 | 0.00036008 | 0.01051084 | CAPSL |
| ENSG00000179902 | 0.38881922 | 0.00044019 | 0.01224649 | C1orf194 |
| ENSG00000187955 | 0.38881736 | 0.00048836 | 0.01333349 | COL14A1 |
| ENSG00000136167 | 0.38829197 | 0.00035294 | 0.0103955 | LCP1 |
| ENSG00000039139 | 0.38709386 | 0.00031667 | 0.00957257 | DNAH5 |
| ENSG00000175556 | 0.38637406 | 0.00060236 | 0.01584584 | LONRF3 |
| ENSG00000079308 | 0.38634779 | 0.00032431 | 0.0097278 | TNS1 |
| ENSG00000135838 | 0.38579725 | 0.00038641 | 0.0111299 | NPL |
| ENSG00000197321 | 0.38538849 | 0.00045102 | 0.01241541 | SVIL |
| ENSG00000144476 | 0.38524935 | 0.0004189 | 0.01187184 | ACKR3 |
| ENSG00000115461 | 0.38506984 | 0.00033077 | 0.00983421 | IGFBP5 |
| ENSG00000182230 | 0.38501444 | 0.00044702 | 0.01233145 | FAM153B |
| ENSG00000092969 | 0.38470179 | 0.00035909 | 0.01050539 | TGFB2 |
| ENSG00000189221 | 0.38455577 | 0.00036119 | 0.01051969 | MAOA |
| ENSG00000167306 | 0.38285697 | 0.00065437 | 0.01677445 | MYO5B |
| ENSG00000147036 | 0.38166634 | 0.00067845 | 0.01722251 | LANCL3 |
| ENSG00000115155 | 0.38137096 | 0.00040218 | 0.01153326 | OTOF |
| ENSG00000116791 | 0.38117767 | 0.00052847 | 0.01424685 | CRYZ |
| ENSG00000117298 | 0.38015884 | 0.00042768 | 0.01207152 | ECE1 |
| ENSG00000164023 | 0.3799324 | 0.00044115 | 0.01224719 | SGMS2 |
| ENSG00000183908 | 0.37965106 | 0.00063048 | 0.0163548 | LRRC55 |
| ENSG00000235437 | 0.37928259 | 0.00061322 | 0.01606678 | LINC01278 |
| ENSG00000134769 | 0.37866587 | 0.00041086 | 0.01173157 | DTNA |
| ENSG00000171595 | 0.37865685 | 0.00064095 | 0.01654775 | DNAI2 |
| ENSG00000134222 | 0.37860808 | 0.00050342 | 0.01365608 | PSRC1 |
| ENSG00000104722 | 0.37859771 | 0.00043612 | 0.01218524 | NEFM |
| ENSG00000242086 | 0.37853029 | 0.00044685 | 0.01233145 | LINC00969 |
| ENSG00000167244 | 0.37827902 | 0.00059637 | 0.01572017 | IGF2 |
| ENSG00000105355 | 0.37821068 | 0.00050914 | 0.01375681 | PLIN3 |
| ENSG00000247157 | 0.3781766 | 0.00070589 | 0.01774645 | LINC01252 |
| ENSG00000276027 | 0.37719239 | 0.00089288 | 0.02125965 | RNU12 |
| ENSG00000243696 | 0.37718943 | 0.00102584 | 0.02390217 | RP5-966M1.6 |
| ENSG00000127418 | 0.37697694 | 0.00053172 | 0.01430491 | FGFRL1 |
| ENSG00000011465 | 0.37667627 | 0.00046969 | 0.0129022 | DCN |
| ENSG00000168026 | 0.37523681 | 0.00062688 | 0.01629376 | TTC21A |
| ENSG00000138074 | 0.37520982 | 0.00056343 | 0.01494222 | SLC5A6 |
| ENSG00000185033 | 0.37489982 | 0.00056732 | 0.01501511 | SEMA4B |
| ENSG00000187486 | 0.37480309 | 0.00067983 | 0.01722418 | KCNJ11 |
| ENSG00000144115 | 0.37475682 | 0.00062206 | 0.01623336 | THNSL2 |
| ENSG00000184371 | 0.37471491 | 0.00057952 | 0.01530673 | CSF1 |
| ENSG00000101347 | 0.37455091 | 0.00050924 | 0.01375681 | SAMHD1 |
| ENSG00000137393 | 0.37388357 | 0.00065832 | 0.01677687 | RNF144B |
| ENSG00000107738 | 0.37330648 | 0.00055285 | 0.01475183 | C10orf54 |
| ENSG00000196739 | 0.3719129 | 0.00078942 | 0.01943474 | COL27A1 |
| ENSG00000163884 | 0.37172238 | 0.00064947 | 0.01668157 | KLF15 |
| ENSG00000147799 | 0.37169321 | 0.00083295 | 0.02008923 | ARHGAP39 |
| ENSG00000157856 | 0.36988673 | 0.00065827 | 0.01677687 | DRC1 |
| ENSG00000054179 | 0.36962215 | 0.00097462 | 0.02295465 | ENTPD2 |
| ENSG00000187984 | 0.3692258 | 0.00094057 | 0.022233 | ANKRD19P |
| ENSG00000065308 | 0.36914196 | 0.00104165 | 0.02418407 | TRAM2 |
| ENSG00000148737 | 0.36908578 | 0.00085754 | 0.02056103 | TCF7L2 |
| ENSG00000197816 | 0.36860251 | 0.00064203 | 0.01654775 | CCDC180 |
| ENSG00000198075 | 0.36825548 | 0.00076439 | 0.01898033 | SULT1C4 |
| ENSG00000169242 | 0.36814158 | 0.00081462 | 0.01975673 | EFNA1 |
| ENSG00000141232 | 0.36811212 | 0.00062554 | 0.01629159 | TOB1 |
| ENSG00000130164 | 0.36744298 | 0.00080052 | 0.01959705 | LDLR |
| ENSG00000106809 | 0.36681813 | 0.00099846 | 0.02343149 | OGN |
| ENSG00000108622 | 0.36579384 | 0.00112796 | 0.02573001 | ICAM2 |
| ENSG00000143384 | 0.36522884 | 0.00067231 | 0.01710013 | MCL1 |
| ENSG00000152377 | 0.36411988 | 0.00069123 | 0.01744518 | SPOCK1 |
| ENSG00000162804 | 0.36391921 | 0.00080772 | 0.01966262 | SNED1 |
| ENSG00000170160 | 0.36383828 | 0.0007328 | 0.01828207 | CCDC144A |
| ENSG00000200320 | 0.36339947 | 0.00071327 | 0.01789754 | SNORA63 |
| ENSG00000128536 | 0.3633004 | 0.00080765 | 0.01966262 | CDHR3 |
| ENSG00000100906 | 0.36320109 | 0.00078067 | 0.01925565 | NFKBIA |
| ENSG00000138792 | 0.3624829 | 0.00109222 | 0.02509031 | ENPEP |
| ENSG00000238622 | 0.36133447 | 0.00094041 | 0.022233 | SNORD97 |
| ENSG00000213694 | 0.3611658 | 0.00095992 | 0.02264915 | S1PR3 |
| ENSG00000034677 | 0.36108656 | 0.00076436 | 0.01898033 | RNF19A |
| ENSG00000183196 | 0.36104386 | 0.00081088 | 0.01970274 | CHST6 |
| ENSG00000161958 | 0.36102972 | 0.00133707 | 0.02951969 | FGF11 |
| ENSG00000196569 | 0.36095499 | 0.00080588 | 0.01966262 | LAMA2 |
| ENSG00000142583 | 0.36049895 | 0.00097972 | 0.02303321 | SLC2A5 |
| ENSG00000186352 | 0.36025631 | 0.00120702 | 0.02701399 | ANKRD37 |
| ENSG00000010327 | 0.35972297 | 0.00090435 | 0.0214544 | STAB1 |
| ENSG00000188933 | 0.35844546 | 0.00090348 | 0.0214544 | USP32P1 |
| ENSG00000138685 | 0.35794842 | 0.00085881 | 0.02056103 | FGF2 |
| ENSG00000275143 | 0.35758968 | 0.0012768 | 0.02833277 | SCARNA16 |
| ENSG00000090376 | 0.35753036 | 0.00119682 | 0.0268317 | IRAK3 |
| ENSG00000141905 | 0.35684308 | 0.00101329 | 0.02365192 | NFIC |
| ENSG00000127311 | 0.35655092 | 0.00116489 | 0.02628501 | HELB |
| ENSG00000171517 | 0.35625779 | 0.00116571 | 0.02628501 | LPAR3 |
| ENSG00000158769 | 0.35462751 | 0.00113246 | 0.02578776 | F11R |
| ENSG00000173546 | 0.35453471 | 0.00117442 | 0.02637488 | CSPG4 |
| ENSG00000109771 | 0.35391028 | 0.00105032 | 0.0242989 | LRP2BP |
| ENSG00000158486 | 0.35363582 | 0.00105875 | 0.02445062 | DNAH3 |
| ENSG00000163840 | 0.35355401 | 0.00114073 | 0.02588566 | DTX3L |
| ENSG00000080854 | 0.3533095 | 0.00122605 | 0.02739306 | IGSF9B |
| ENSG00000167470 | 0.35318102 | 0.00152287 | 0.03268158 | MIDN |
| ENSG00000253741 | 0.3528726 | 0.001587 | 0.03350683 | CTD-2292P10.4 |
| ENSG00000133401 | 0.35263489 | 0.00100276 | 0.02349009 | PDZD2 |
| ENSG00000142609 | 0.35235209 | 0.00140739 | 0.03070841 | CFAP74 |
| ENSG00000169302 | 0.35217039 | 0.00116639 | 0.02628501 | STK32A |
| ENSG00000153246 | 0.3520991 | 0.0017367 | 0.03574208 | PLA2R1 |
| ENSG00000258752 | 0.35179454 | 0.00137949 | 0.03030224 | RP11-356K23.1 |
| ENSG00000067082 | 0.35057971 | 0.00123 | 0.02743415 | KLF6 |
| ENSG00000105971 | 0.35054586 | 0.0015332 | 0.0327083 | CAV2 |
| ENSG00000106991 | 0.35006837 | 0.00167153 | 0.03472944 | ENG |
| ENSG00000067955 | 0.34975486 | 0.00125369 | 0.02791488 | CBFB |
| ENSG00000137198 | 0.34893931 | 0.00134452 | 0.02958402 | GMPR |
| ENSG00000140092 | 0.34890904 | 0.00127012 | 0.02823259 | FBLN5 |
| ENSG00000131981 | 0.34876146 | 0.00153156 | 0.0327083 | LGALS3 |
| ENSG00000253284 | 0.34862193 | 0.00139976 | 0.03062075 | RP11-282K24.3 |
| ENSG00000207405 | 0.34855933 | 0.00176803 | 0.03621546 | SNORA64 |
| ENSG00000103257 | 0.3475823 | 0.00134389 | 0.02958402 | SLC7A5 |
| ENSG00000143554 | 0.34722162 | 0.00222334 | 0.04266197 | SLC27A3 |
| ENSG00000253537 | 0.34656736 | 0.00186769 | 0.03760741 | PCDHGA7 |
| ENSG00000181826 | 0.34645097 | 0.00169508 | 0.03516279 | RELL1 |
| ENSG00000140090 | 0.34623799 | 0.00140103 | 0.03062075 | SLC24A4 |
| ENSG00000188803 | 0.34612561 | 0.00209949 | 0.04100918 | SHISA6 |
| ENSG00000164692 | 0.34574749 | 0.00146699 | 0.03171443 | COL1A2 |
| ENSG00000135636 | 0.34562629 | 0.0014317 | 0.03118677 | DYSF |
| ENSG00000177119 | 0.34466617 | 0.0013886 | 0.03045113 | ANO6 |
| ENSG00000058866 | 0.34340514 | 0.00150077 | 0.03226027 | DGKG |
| ENSG00000136436 | 0.34277365 | 0.00144486 | 0.0313686 | CALCOCO2 |
| ENSG00000121898 | 0.34258833 | 0.00243438 | 0.0453571 | CPXM2 |
| ENSG00000253159 | 0.34235539 | 0.00166603 | 0.03467033 | PCDHGA12 |
| ENSG00000177994 | 0.3422011 | 0.00244027 | 0.0453571 | C2orf73 |
| ENSG00000149499 | 0.34194915 | 0.0018229 | 0.03705781 | EML3 |
| ENSG00000237172 | 0.34187855 | 0.00203781 | 0.04010452 | B3GNT9 |
| ENSG00000163430 | 0.34143796 | 0.00148093 | 0.03188638 | FSTL1 |
| ENSG00000258818 | 0.34140479 | 0.00206551 | 0.04052755 | RNASE4 |
| ENSG00000118515 | 0.34129365 | 0.00147998 | 0.03188638 | SGK1 |
| ENSG00000153347 | 0.34123663 | 0.00178134 | 0.03643091 | FAM81B |
| ENSG00000156206 | 0.34078832 | 0.00210475 | 0.041049 | C15orf26 |
| ENSG00000111837 | 0.34077927 | 0.00224559 | 0.04300392 | MAK |
| ENSG00000184640 | 0.34009689 | 0.00160213 | 0.03366289 | SEPTIN9 |
| ENSG00000159423 | 0.33995136 | 0.00164104 | 0.03429501 | ALDH4A1 |
| ENSG00000144668 | 0.33972676 | 0.00194775 | 0.03880047 | ITGA9 |
| ENSG00000069535 | 0.33966442 | 0.00154516 | 0.03288952 | MAOB |
| ENSG00000128591 | 0.33939415 | 0.00164273 | 0.03429501 | FLNC |
| ENSG00000163659 | 0.33937736 | 0.00163594 | 0.03426279 | TIPARP |
| ENSG00000200087 | 0.33921061 | 0.00155571 | 0.03301131 | SNORA73B |
| ENSG00000173838 | 0.33896645 | 0.00221367 | 0.04253889 | MARCHF10 |
| ENSG00000197826 | 0.33868526 | 0.00209398 | 0.04100918 | C4orf22 |
| ENSG00000114251 | 0.33839169 | 0.00259314 | 0.04765528 | WNT5A |
| ENSG00000196924 | 0.33836403 | 0.00161368 | 0.03385105 | FLNA |
| ENSG00000141736 | 0.33784366 | 0.00176699 | 0.03621546 | ERBB2 |
| ENSG00000184828 | 0.337312 | 0.00237879 | 0.04472407 | ZBTB7C |
| ENSG00000131941 | 0.33678522 | 0.00206103 | 0.04050045 | RHPN2 |
| ENSG00000119138 | 0.33648922 | 0.00172987 | 0.03565766 | KLF9 |
| ENSG00000175183 | 0.33641681 | 0.00189629 | 0.03806575 | CSRP2 |
| ENSG00000018408 | 0.33609341 | 0.00176537 | 0.03621546 | WWTR1 |
| ENSG00000154734 | 0.33595493 | 0.00194165 | 0.03879737 | ADAMTS1 |
| ENSG00000105855 | 0.33586711 | 0.00172013 | 0.03556937 | ITGB8 |
| ENSG00000056558 | 0.33556283 | 0.00216669 | 0.04194502 | TRAF1 |
| ENSG00000144649 | 0.33485112 | 0.00211697 | 0.0411658 | FAM198A |
| ENSG00000157514 | 0.33425505 | 0.00189927 | 0.03806705 | TSC22D3 |
| ENSG00000175106 | 0.33411484 | 0.00254358 | 0.0469429 | TVP23C |
| ENSG00000060656 | 0.33392692 | 0.00236006 | 0.04456448 | PTPRU |
| ENSG00000168994 | 0.33388924 | 0.00251718 | 0.04665362 | PXDC1 |
| ENSG00000014914 | 0.33384246 | 0.00243876 | 0.0453571 | MTMR11 |
| ENSG00000178966 | 0.33365367 | 0.00263507 | 0.04828996 | RMI1 |
| ENSG00000003989 | 0.33359691 | 0.00196598 | 0.03904436 | SLC7A2 |
| ENSG00000100003 | 0.33341475 | 0.00202958 | 0.04000301 | SEC14L2 |
| ENSG00000078295 | 0.33326689 | 0.00190657 | 0.03815479 | ADCY2 |
| ENSG00000163565 | 0.33288628 | 0.00224775 | 0.04300392 | IFI16 |
| ENSG00000133665 | 0.33239565 | 0.00227362 | 0.04318224 | DYDC2 |
| ENSG00000105519 | 0.33218076 | 0.00196355 | 0.03904436 | CAPS |
| ENSG00000152137 | 0.331677 | 0.00198574 | 0.03934819 | HSPB8 |
| ENSG00000163071 | 0.33147205 | 0.00210782 | 0.041049 | SPATA18 |
| ENSG00000280237 | 0.33139498 | 0.00218998 | 0.04227045 | MIR4697HG |
| ENSG00000142871 | 0.33098323 | 0.00238926 | 0.04485612 | CYR61 |
| ENSG00000179630 | 0.3307322 | 0.00226383 | 0.04312186 | LACC1 |
| ENSG00000158270 | 0.33005748 | 0.00220614 | 0.04245675 | COLEC12 |
| ENSG00000155850 | 0.32993854 | 0.00241367 | 0.04524835 | SLC26A2 |
| ENSG00000107551 | 0.32834955 | 0.00226366 | 0.04312186 | RASSF4 |
| ENSG00000168710 | 0.32807625 | 0.0021941 | 0.04228751 | AHCYL1 |
| ENSG00000153902 | 0.32757576 | 0.00253188 | 0.04682707 | LGI4 |
| ENSG00000115414 | 0.32745981 | 0.00226011 | 0.04312186 | FN1 |
| ENSG00000166833 | 0.32744915 | 0.00231457 | 0.04383237 | NAV2 |
| ENSG00000125398 | 0.32634911 | 0.00242055 | 0.04524835 | SOX9 |
| ENSG00000008118 | 0.32586244 | 0.00253371 | 0.04682707 | CAMK1G |
| ENSG00000169871 | 0.32528373 | 0.00267141 | 0.04886543 | TRIM56 |
| ENSG00000167995 | 0.3244098 | 0.00267397 | 0.04886543 | BEST1 |
| ENSG00000105662 | 0.32339942 | 0.00272023 | 0.04957201 | CRTC1 |
| ENSG00000136870 | 0.32332656 | 0.00262463 | 0.04816621 | ZNF189 |
| ENSG00000101438 | 0.32328767 | 0.00273022 | 0.0496847 | SLC32A1 |
| ENSG00000105894 | -0.3212586 | 0.00270356 | 0.04933717 | PTN |
| ENSG00000164951 | -0.3229776 | 0.00250247 | 0.04644698 | PDP1 |
| ENSG00000198300 | -0.3248998 | 0.00233167 | 0.0440922 | PEG3 |
| ENSG00000274956 | -0.3251159 | 0.00237194 | 0.04472405 | UG0898H09 |
| ENSG00000130558 | -0.32562 | 0.00242794 | 0.04532153 | OLFM1 |
| ENSG00000164588 | -0.3256998 | 0.00274221 | 0.04983341 | HCN1 |
| ENSG00000049089 | -0.3260889 | 0.00258599 | 0.0475909 | COL9A2 |
| ENSG00000170091 | -0.3274783 | 0.00215924 | 0.04186286 | HMP19 |
| ENSG00000133116 | -0.3284975 | 0.00237685 | 0.04472407 | KL |
| ENSG00000126733 | -0.329592 | 0.00226716 | 0.04312234 | DACH2 |
| ENSG00000164794 | -0.3301909 | 0.00230482 | 0.04371127 | KCNV1 |
| ENSG00000102003 | -0.3306503 | 0.00198731 | 0.03934819 | SYP |
| ENSG00000104332 | -0.3321419 | 0.00209648 | 0.04100918 | SFRP1 |
| ENSG00000181409 | -0.3322786 | 0.00201407 | 0.03975733 | AATK |
| ENSG00000135824 | -0.3331259 | 0.00182687 | 0.0370716 | RGS8 |
| ENSG00000109738 | -0.3334629 | 0.00188732 | 0.03794423 | GLRB |
| ENSG00000165246 | -0.3340334 | 0.00194489 | 0.03880047 | NLGN4Y |
| ENSG00000165023 | -0.3356244 | 0.00172411 | 0.03559516 | DIRAS2 |
| ENSG00000166073 | -0.3357856 | 0.00179343 | 0.03662076 | GPR176 |
| ENSG00000079931 | -0.3368693 | 0.00185223 | 0.03746968 | MOXD1 |
| ENSG00000203772 | -0.337323 | 0.00255275 | 0.04704567 | SPRN |
| ENSG00000173391 | -0.3378063 | 0.00215049 | 0.04175541 | OLR1 |
| ENSG00000185518 | -0.3379426 | 0.00158687 | 0.03350683 | SV2B |
| ENSG00000175906 | -0.3381459 | 0.00241748 | 0.04524835 | ARL4D |
| ENSG00000107105 | -0.3400992 | 0.00171554 | 0.03553083 | ELAVL2 |
| ENSG00000103089 | -0.3401574 | 0.00185897 | 0.03754644 | FA2H |
| ENSG00000118432 | -0.3403891 | 0.00145224 | 0.03147639 | CNR1 |
| ENSG00000013293 | -0.3408903 | 0.00144186 | 0.03135561 | SLC7A14 |
| ENSG00000249992 | -0.3415962 | 0.00155594 | 0.03301131 | TMEM158 |
| ENSG00000205704 | -0.3424279 | 0.00200878 | 0.03971299 | LINC00634 |
| ENSG00000162670 | -0.3430491 | 0.00159456 | 0.03355774 | BRINP3 |
| ENSG00000092758 | -0.3441581 | 0.00180809 | 0.0368624 | COL9A3 |
| ENSG00000279207 | -0.3456585 | 0.0015316 | 0.0327083 | RP11-159D12.8 |
| ENSG00000140406 | -0.34567 | 0.00146808 | 0.03171443 | MESDC1 |
| ENSG00000163630 | -0.3471562 | 0.00115445 | 0.02615148 | SYNPR |
| ENSG00000187135 | -0.3476453 | 0.00159256 | 0.03355774 | VSTM2B |
| ENSG00000198223 | -0.349463 | 0.00217945 | 0.04212958 | CSF2RA |
| ENSG00000027075 | -0.3497062 | 0.00113694 | 0.02584457 | PRKCH |
| ENSG00000119698 | -0.3502284 | 0.00109709 | 0.02515785 | PPP4R4 |
| ENSG00000187676 | -0.3504231 | 0.0010901 | 0.02508573 | B3GALTL |
| ENSG00000175899 | -0.351357 | 0.00100853 | 0.02358297 | A2M |
| ENSG00000118298 | -0.3513611 | 0.00182335 | 0.03705781 | CA14 |
| ENSG00000173621 | -0.3518166 | 0.00186178 | 0.03754644 | LRFN4 |
| ENSG00000082497 | -0.3522055 | 0.0011147 | 0.0255168 | SERTAD4 |
| ENSG00000169562 | -0.3522971 | 0.00128541 | 0.02847538 | GJB1 |
| ENSG00000182508 | -0.3529417 | 0.00156199 | 0.03308575 | LHFPL1 |
| ENSG00000269929 | -0.3530108 | 0.00184067 | 0.03729357 | RP11-2B6.2 |
| ENSG00000183160 | -0.3532663 | 0.00166538 | 0.03467033 | TMEM119 |
| ENSG00000240342 | -0.3536467 | 0.00129052 | 0.02854018 | RPS2P5 |
| ENSG00000198797 | -0.354941 | 0.00106264 | 0.02449715 | BRINP2 |
| ENSG00000134986 | -0.3556306 | 0.00102852 | 0.0239218 | NREP |
| ENSG00000174607 | -0.3570081 | 0.00084599 | 0.02032872 | UGT8 |
| ENSG00000133216 | -0.3572418 | 0.00112601 | 0.02573001 | EPHB2 |
| ENSG00000182050 | -0.3596051 | 0.00081718 | 0.01978207 | MGAT4C |
| ENSG00000144834 | -0.3599392 | 0.00077208 | 0.01911605 | TAGLN3 |
| ENSG00000148734 | -0.3601758 | 0.00117123 | 0.02634855 | NPFFR1 |
| ENSG00000105963 | -0.3605899 | 0.00084168 | 0.02026234 | ADAP1 |
| ENSG00000148826 | -0.3618449 | 0.00104988 | 0.0242989 | NKX6-2 |
| ENSG00000095585 | -0.361849 | 0.00153414 | 0.0327083 | BLNK |
| ENSG00000143171 | -0.3622112 | 0.00079507 | 0.01950005 | RXRG |
| ENSG00000156076 | -0.3659906 | 0.00065568 | 0.01677522 | WIF1 |
| ENSG00000188730 | -0.3669863 | 0.00082091 | 0.01983567 | VWC2 |
| ENSG00000133636 | -0.3675602 | 0.0007326 | 0.01828207 | NTS |
| ENSG00000025423 | -0.3687996 | 0.00070363 | 0.01772375 | HSD17B6 |
| ENSG00000188674 | -0.3689219 | 0.00076515 | 0.01898033 | C2orf80 |
| ENSG00000197261 | -0.3689855 | 0.00064299 | 0.01654775 | C6orf141 |
| ENSG00000161682 | -0.3693446 | 0.00088099 | 0.02105349 | FAM171A2 |
| ENSG00000234377 | -0.3698262 | 0.00061624 | 0.01611368 | RNF219-AS1 |
| ENSG00000214941 | -0.370064 | 0.0007916 | 0.01945147 | ZSWIM7 |
| ENSG00000164841 | -0.3703475 | 0.00088516 | 0.02111439 | TMEM74 |
| ENSG00000182636 | -0.3707314 | 0.00056206 | 0.01493639 | NDN |
| ENSG00000060140 | -0.3713461 | 0.00071816 | 0.01798572 | STYK1 |
| ENSG00000133135 | -0.3723655 | 0.00077674 | 0.01919491 | RNF128 |
| ENSG00000196368 | -0.3746299 | 0.00053481 | 0.01432887 | NUDT11 |
| ENSG00000095203 | -0.3759578 | 0.00048846 | 0.01333349 | EPB41L4B |
| ENSG00000145569 | -0.3763451 | 0.00068244 | 0.01725682 | FAM105A |
| ENSG00000162636 | -0.3789277 | 0.00044691 | 0.01233145 | FAM102B |
| ENSG00000166394 | -0.3804364 | 0.00064117 | 0.01654775 | CYB5R2 |
| ENSG00000198822 | -0.3819875 | 0.0003551 | 0.01043544 | GRM3 |
| ENSG00000186297 | -0.3834064 | 0.00034067 | 0.01005671 | GABRA5 |
| ENSG00000136160 | -0.3839201 | 0.00032998 | 0.00983421 | EDNRB |
| ENSG00000213626 | -0.3843623 | 0.00053328 | 0.01431735 | LBH |
| ENSG00000164591 | -0.384953 | 0.00060859 | 0.01597768 | MYOZ3 |
| ENSG00000041353 | -0.3859829 | 0.00038353 | 0.01107159 | RAB27B |
| ENSG00000261236 | -0.3866352 | 0.00041767 | 0.01187184 | BOP1 |
| ENSG00000011677 | -0.3884579 | 0.00041179 | 0.01173157 | GABRA3 |
| ENSG00000040731 | -0.3905039 | 0.00033242 | 0.00985772 | CDH10 |
| ENSG00000154162 | -0.3914528 | 0.0003133 | 0.00950682 | CDH12 |
| ENSG00000233705 | -0.391644 | 0.00049087 | 0.01337132 | SLC26A4-AS1 |
| ENSG00000162374 | -0.3938808 | 0.00043186 | 0.0121442 | ELAVL4 |
| ENSG00000152413 | -0.395587 | 0.00021877 | 0.00697923 | HOMER1 |
| ENSG00000111181 | -0.3963697 | 0.00028853 | 0.00881658 | SLC6A12 |
| ENSG00000134207 | -0.3966221 | 0.00026161 | 0.0081081 | SYT6 |
| ENSG00000146070 | -0.3974519 | 0.00041944 | 0.01187184 | PLA2G7 |
| ENSG00000115665 | -0.3981323 | 0.0002645 | 0.00815883 | SLC5A7 |
| ENSG00000198785 | -0.3982673 | 0.00037866 | 0.01097938 | GRIN3A |
| ENSG00000171551 | -0.3994732 | 0.00043397 | 0.01215111 | ECEL1 |
| ENSG00000179841 | -0.3994747 | 0.00018646 | 0.00606718 | AKAP5 |
| ENSG00000153820 | -0.3997543 | 0.00022057 | 0.00701964 | SPHKAP |
| ENSG00000174370 | -0.4044277 | 0.00033087 | 0.00983421 | C11orf45 |
| ENSG00000244405 | -0.4045434 | 0.00016739 | 0.00552926 | ETV5 |
| ENSG00000140749 | -0.4048475 | 0.00033523 | 0.00991868 | IGSF6 |
| ENSG00000106089 | -0.407136 | 0.00020704 | 0.00667026 | STX1A |
| ENSG00000102468 | -0.4075094 | 0.00016336 | 0.00541009 | HTR2A |
| ENSG00000163536 | -0.4085331 | 0.00013696 | 0.00462959 | SERPINI1 |
| ENSG00000163032 | -0.4096051 | 0.00013528 | 0.0045849 | VSNL1 |
| ENSG00000124785 | -0.4117282 | 0.00023959 | 0.00751476 | NRN1 |
| ENSG00000137727 | -0.4132432 | 0.00011542 | 0.00402682 | ARHGAP20 |
| ENSG00000082556 | -0.4134251 | 0.00012535 | 0.00428143 | OPRK1 |
| ENSG00000263934 | -0.4138605 | 0.00022517 | 0.00714857 | SNORD3A |
| ENSG00000146360 | -0.415289 | 0.00012345 | 0.00423897 | GPR6 |
| ENSG00000067715 | -0.416734 | 9.58E-05 | 0.00342589 | SYT1 |
| ENSG00000164604 | -0.4172762 | 0.00010805 | 0.00381028 | GPR85 |
| ENSG00000119125 | -0.4187872 | 9.39E-05 | 0.00336698 | GDA |
| ENSG00000172995 | -0.4192629 | 8.60E-05 | 0.00314359 | ARPP21 |
| ENSG00000171617 | -0.4194089 | 8.70E-05 | 0.00315205 | ENC1 |
| ENSG00000164099 | -0.4196754 | 0.0002173 | 0.00694941 | PRSS12 |
| ENSG00000147246 | -0.4197443 | 8.69E-05 | 0.00315205 | HTR2C |
| ENSG00000152214 | -0.4268197 | 0.00011361 | 0.00398474 | RIT2 |
| ENSG00000166006 | -0.4269575 | 0.00011477 | 0.00401467 | KCNC2 |
| ENSG00000116741 | -0.4301519 | 6.18E-05 | 0.00239279 | RGS2 |
| ENSG00000104327 | -0.4302005 | 5.71E-05 | 0.00226446 | CALB1 |
| ENSG00000281106 | -0.4311071 | 5.94E-05 | 0.00234638 | LINC00282 |
| ENSG00000010319 | -0.4341285 | 9.26E-05 | 0.00333589 | SEMA3G |
| ENSG00000121769 | -0.4344133 | 7.33E-05 | 0.00277206 | FABP3 |
| ENSG00000112232 | -0.4355044 | 6.31E-05 | 0.00243576 | KHDRBS2 |
| ENSG00000163492 | -0.4387783 | 5.97E-05 | 0.00234638 | CCDC141 |
| ENSG00000171509 | -0.4388473 | 9.12E-05 | 0.00329609 | RXFP1 |
| ENSG00000145632 | -0.4393051 | 4.00E-05 | 0.0016937 | PLK2 |
| ENSG00000180616 | -0.4412665 | 4.02E-05 | 0.00169442 | SSTR2 |
| ENSG00000119508 | -0.4426101 | 5.13E-05 | 0.00207106 | NR4A3 |
| ENSG00000113100 | -0.4428144 | 4.57E-05 | 0.00188011 | CDH9 |
| ENSG00000146250 | -0.444272 | 6.00E-05 | 0.00234729 | PRSS35 |
| ENSG00000157833 | -0.4442847 | 4.15E-05 | 0.00172572 | GAREML |
| ENSG00000068976 | -0.4468215 | 3.54E-05 | 0.00151521 | PYGM |
| ENSG00000102109 | -0.4484551 | 4.03E-05 | 0.00169442 | PCSK1N |
| ENSG00000175426 | -0.4486268 | 3.31E-05 | 0.00142906 | PCSK1 |
| ENSG00000170340 | -0.4509949 | 2.67E-05 | 0.00118297 | B3GNT2 |
| ENSG00000170381 | -0.4511444 | 4.08E-05 | 0.00170696 | SEMA3E |
| ENSG00000148123 | -0.4544889 | 2.33E-05 | 0.00104567 | RP11-35N6.1 |
| ENSG00000017427 | -0.4549884 | 5.95E-05 | 0.00234638 | IGF1 |
| ENSG00000165370 | -0.4576023 | 2.90E-05 | 0.00127247 | GPR101 |
| ENSG00000006128 | -0.4600447 | 1.73E-05 | 0.00081195 | TAC1 |
| ENSG00000114646 | -0.4615859 | 1.78E-05 | 0.00082562 | CSPG5 |
| ENSG00000256500 | -0.4681172 | 3.24E-05 | 0.00140597 | RP11-73M18.2 |
| ENSG00000067646 | -0.4721742 | 1.49E-05 | 0.00070951 | ZFY |
| ENSG00000100473 | -0.4721904 | 1.00E-05 | 0.00051144 | COCH |
| ENSG00000172137 | -0.4725489 | 1.88E-05 | 0.00086502 | CALB2 |
| ENSG00000108960 | -0.4761689 | 8.71E-06 | 0.00044733 | MMD |
| ENSG00000011638 | -0.4813726 | 9.14E-06 | 0.0004676 | TMEM159 |
| ENSG00000105976 | -0.4842201 | 1.75E-05 | 0.00081478 | MET |
| ENSG00000173114 | -0.4846854 | 6.51E-06 | 0.00034543 | LRRN3 |
| ENSG00000171951 | -0.4854123 | 5.56E-06 | 0.00030373 | SCG2 |
| ENSG00000103811 | -0.486858 | 5.99E-06 | 0.00032314 | CTSH |
| ENSG00000118946 | -0.490134 | 4.75E-06 | 0.00026156 | PCDH17 |
| ENSG00000086570 | -0.4938165 | 4.29E-06 | 0.00023839 | FAT2 |
| ENSG00000140545 | -0.4983581 | 4.59E-06 | 0.00025366 | MFGE8 |
| ENSG00000198835 | -0.505255 | 7.96E-06 | 0.00041373 | GJC2 |
| ENSG00000136999 | -0.5082191 | 6.20E-06 | 0.00033265 | NOV |
| ENSG00000134917 | -0.5089684 | 3.82E-06 | 0.00021564 | ADAMTS8 |
| ENSG00000125848 | -0.5107038 | 3.23E-06 | 0.00018314 | FLRT3 |
| ENSG00000110876 | -0.5128628 | 2.88E-06 | 0.0001663 | SELPLG |
| ENSG00000117069 | -0.5151434 | 1.93E-06 | 0.00011473 | ST6GALNAC5 |
| ENSG00000175874 | -0.5235025 | 1.16E-06 | 7.29E-05 | CREG2 |
| ENSG00000139278 | -0.5243953 | 1.58E-06 | 9.73E-05 | GLIPR1 |
| ENSG00000132872 | -0.5275038 | 8.28E-07 | 5.29E-05 | SYT4 |
| ENSG00000175445 | -0.5280132 | 7.85E-07 | 5.10E-05 | LPL |
| ENSG00000128564 | -0.5312825 | 1.30E-06 | 8.01E-05 | VGF |
| ENSG00000173705 | -0.5329034 | 1.28E-06 | 7.98E-05 | SUSD5 |
| ENSG00000168329 | -0.5403796 | 5.32E-07 | 3.60E-05 | CX3CR1 |
| ENSG00000121858 | -0.5503003 | 8.14E-07 | 5.23E-05 | TNFSF10 |
| ENSG00000197629 | -0.550974 | 7.67E-07 | 5.00E-05 | MPEG1 |
| ENSG00000169860 | -0.5673086 | 1.19E-07 | 8.93E-06 | P2RY1 |
| ENSG00000111885 | -0.5750795 | 8.14E-08 | 6.14E-06 | MAN1A1 |
| ENSG00000180332 | -0.5922315 | 3.91E-08 | 3.08E-06 | KCTD4 |
| ENSG00000167588 | -0.5970583 | 4.51E-08 | 3.51E-06 | GPD1 |
| ENSG00000162630 | -0.5981437 | 3.72E-08 | 2.96E-06 | B3GALT2 |
| ENSG00000136235 | -0.6005432 | 3.91E-08 | 3.08E-06 | GPNMB |
| ENSG00000172020 | -0.602857 | 1.89E-08 | 1.62E-06 | GAP43 |
| ENSG00000165434 | -0.6102715 | 1.25E-08 | 1.10E-06 | PGM2L1 |
| ENSG00000053438 | -0.6109223 | 1.15E-08 | 1.02E-06 | NNAT |
| ENSG00000017483 | -0.6234016 | 2.70E-08 | 2.21E-06 | SLC38A5 |
| ENSG00000151577 | -0.6253102 | 2.76E-08 | 2.25E-06 | DRD3 |
| ENSG00000162595 | -0.6267141 | 8.92E-09 | 8.20E-07 | DIRAS3 |
| ENSG00000227544 | -0.6332682 | 8.20E-09 | 7.65E-07 | AC018647.3 |
| ENSG00000141485 | -0.6366683 | 2.29E-08 | 1.92E-06 | SLC13A5 |
| ENSG00000054803 | -0.6472517 | 1.01E-08 | 9.16E-07 | CBLN4 |
| ENSG00000180730 | -0.6490354 | 7.45E-09 | 7.09E-07 | SHISA2 |
| ENSG00000118777 | -0.6687545 | 5.41E-10 | 5.98E-08 | ABCG2 |
| ENSG00000181195 | -0.678385 | 2.30E-10 | 2.80E-08 | PENK |
| ENSG00000181631 | -0.6804843 | 1.07E-09 | 1.13E-07 | P2RY13 |
| ENSG00000172568 | -0.6995336 | 2.88E-10 | 3.36E-08 | FNDC9 |
| ENSG00000166428 | -0.7190412 | 2.23E-10 | 2.75E-08 | PLD4 |
| ENSG00000103316 | -0.7653766 | 9.74E-13 | 1.55E-10 | CRYM |
| ENSG00000101958 | -0.7765015 | 3.77E-12 | 5.41E-10 | GLRA2 |
| ENSG00000259024 | -0.813384 | 4.57E-13 | 7.46E-11 | TVP23C-CDRT4 |
| ENSG00000118271 | -0.8152566 | 4.77E-14 | 9.29E-12 | TTR |
| ENSG00000169313 | -0.9719509 | 4.67E-19 | 1.60E-16 | P2RY12 |
| ENSG00000101327 | -0.9741119 | 1.37E-19 | 4.83E-17 | PDYN |
