# Supplementary material for: RNA-sequencing suggests extracellular matrix and vasculature dysregulation could impair neurogenesis in schizophrenia cases with elevated inflammation
Source: Schizophrenia (Heidelb). 2024 May 4;10(1):50. doi: 10.1038/s41537-024-00466-0 (PMC11069512; doi:10.1038/s41537-024-00466-0)
Supplement: Supplementary file 3 — Appendix Table 3 [file 41537_2024_466_MOESM3_ESM.docx]

**Appendix Table 3**. Ingenuity pathway analysis (© 2000-2020 QIAGEN) comparing differentially expressed genes between schizophrenia cases with high and low inflammation, with a FDR adjusted q < 0.05. Blank under Z-score represents pathways ineligible for z-score prediction (based on inadequate information in the IPA knowledge base)

| **Ingenuity Canonical Pathways** | **-log(p-value)** | **Ratio** | **z-score** | **Molecules** |
| --- | --- | --- | --- | --- |
| Hepatic Fibrosis / Hepatic Stellate Cell Activation | 23.6 | 0.22 |  | A2M,ACTA2,CCN2,CD14,COL1A1,COL1A2,COL27A1,COL3A1,COL4A1,COL4A2,COL5A1,COL6A3,COL7A1,COL8A1,COL9A2,COL9A3,CSF1,ECE1,EDN1,EDNRB,FGF2,FLT1,FLT4,FN1,ICAM1,IGF1,IGF2,IGFBP4,IGFBP5,IL1R1,IL4R,KLF6,MET,MYH11,MYL9,TGFB2,TIMP1,TNFRSF11B,TNFRSF1A,TNFRSF1B,VEGFA |
| Acute Phase Response Signaling | 9.95 | 0.14 | 3.3 | A2M,C1R,C1S,C4A/C4B,CEBPB,CFB,CP,FN1,HMOX1,IL1R1,NFKBIA,OSMR,RASD1,SERPINA1,SERPINA3,SERPING1,SHC1,SOCS3,SOD2,STAT3,TNFRSF11B,TNFRSF1A,TNFRSF1B,TTR,VWF |
| Hepatic Fibrosis Signaling Pathway | 8.33 | 0.0924 | 4.116 | ACTA2,CCN2,CEBPB,CNR1,COL1A1,COL1A2,COL3A1,DIRAS3,EDN1,FGF2,FLT1,FLT4,ICAM1,IL1R1,IRAK3,ITGA5,KLF9,MYL9,MYLK3,NFKBIA,PDK1,PRKCH,RASD1,SOD2,SPP1,STAT3,TCF7L2,TGFB2,TIMP1,TNFRSF11B,TNFRSF1A,TNFRSF1B,VEGFA,WNT5A |
| Complement System | 8.25 | 0.297 | 0.632 | C1QA,C1QB,C1QC,C1R,C1S,C4A/C4B,C7,CFB,CFH,CFI,SERPING1 |
| IL-6 Signaling | 6.07 | 0.128 | 2.673 | A2M,CD14,CEBPB,COL1A1,HSPB1,IL1R1,MCL1,NFKBIA,RASD1,SHC1,SOCS3,STAT3,TNFRSF11B,TNFRSF1A,TNFRSF1B,VEGFA |
| Osteoarthritis Pathway | 5.9 | 0.0995 | 3.153 | ACVRL1,ALPL,ANXA2,CASP7,CEBPB,DCN,DDIT4,DDR2,FGF2,FN1,IL1R1,ITGA5,NAMPT,S1PR3,SOX9,SPP1,TCF7L2,TLR2,TNFRSF1A,TNFRSF1B,VEGFA |
| GP6 Signaling Pathway | 5.64 | 0.126 | 2.324 | COL1A1,COL1A2,COL27A1,COL3A1,COL4A1,COL4A2,COL5A1,COL6A3,COL7A1,COL8A1,COL9A2,COL9A3,LAMA2,LAMA5,PRKCH |
| Role of Macrophages, Fibroblasts and Endothelial Cells in Rheumatoid Arthritis | 5.17 | 0.0801 |  | CEBPB,CSF1,FCGR3A/FCGR3B,FGF2,FN1,ICAM1,IL1R1,IRAK3,NFKBIA,PLCD1,PLCE1,PRKCH,RASD1,SFRP1,SOCS3,STAT3,TCF7L2,TLR2,TNFRSF11B,TNFRSF1A,TNFRSF1B,TRAF1,VEGFA,WIF1,WNT5A |
| IGF-1 Signaling | 4.93 | 0.125 | 0.447 | CCN1,CCN2,CCN3,IGF1,IGFBP2,IGFBP4,IGFBP5,IGFBP7,PXN,RASD1,SHC1,SOCS3,STAT3 |
| VDR/RXR Activation | 4.76 | 0.141 | 0.707 | CALB1,CD14,CDKN1A,CEBPB,GADD45A,IGFBP5,PRKCH,RXRG,SERPINB1,SPP1,TGFB2 |
| Atherosclerosis Signaling | 4.67 | 0.111 |  | APOD,APOE,COL1A1,COL1A2,COL3A1,CSF1,F3,ICAM1,LPL,PLA2G7,PLA2R1,PLAAT4,SELPLG,SERPINA1 |
| FAT10 Cancer Signaling Pathway | 4.28 | 0.174 | 2.121 | ACKR3,NFKBIA,STAT3,TGFB2,TNFRSF11B,TNFRSF1A,TNFRSF1B,TP53 |
| LXR/RXR Activation | 4.23 | 0.107 | -0.577 | APOD,APOE,C4A/C4B,CD14,IL1R1,LDLR,LPL,RXRG,SERPINA1,TNFRSF11B,TNFRSF1A,TNFRSF1B,TTR |
| LPS/IL-1 Mediated Inhibition of RXR Function | 3.91 | 0.0804 | 1.134 | ACSL5,ALDH1L1,ALDH1L2,ALDH4A1,APOE,CD14,CHST3,FABP3,IL1R1,MAOA,MAOB,MGST1,PAPSS2,SLC27A3,SULT1C4,TNFRSF11B,TNFRSF1A,TNFRSF1B |
| STAT3 Pathway | 3.74 | 0.0963 | 1.667 | CDKN1A,FGF2,FLT1,FLT4,IGF1,IL1R1,IL4R,PIM1,RASD1,SOCS3,STAT3,TGFB2,VEGFA |
| HOTAIR Regulatory Pathway | 3.57 | 0.0881 | 2.138 | CD44,CDKN1A,COL1A1,COL1A2,ERBB2,ICAM1,MET,NFKBIA,SPP1,STAT3,TCF7L2,VIM,WIF1,WNT5A |
| Aryl Hydrocarbon Receptor Signaling | 3.49 | 0.0909 | -0.816 | ALDH1L1,ALDH1L2,ALDH4A1,CDKN1A,CYP1B1,HSPB1,MGST1,NFIC,NQO1,RXRG,TGFB2,TGM2,TP53 |
| Gαi Signaling | 3.48 | 0.096 | -0.905 | ADCY2,APLNR,CNR1,DRD3,GRM3,OPRK1,P2RY12,P2RY13,RASD1,S1PR3,SHC1,STAT3 |
| Sperm Motility | 3.46 | 0.0762 | 0.816 | AATK,CSF2RA,DDR2,EPHB2,ERBB2,FLT1,FLT4,JAK3,MET,MUSK,PLA2G7,PLA2R1,PLAAT4,PLCD1,PLCE1,PRKCH,STYK1 |
| Antioxidant Action of Vitamin C | 3.43 | 0.101 | -1 | CSF2RA,GLRX,HMOX1,NFKBIA,PLA2G7,PLA2R1,PLAAT4,PLCD1,PLCE1,PLD4,SLC2A5 |
| Endothelin-1 Signaling | 3.32 | 0.0798 | 0.832 | ADCY2,CASP7,ECE1,EDN1,EDNRB,HMOX1,PLA2G7,PLA2R1,PLAAT4,PLCD1,PLCE1,PLD4,PRKCH,RASD1,SHC1 |
| Coagulation System | 3.3 | 0.171 | 0.816 | A2M,F13A1,F3,F5,SERPINA1,VWF |
| Tumoricidal Function of Hepatic Natural Killer Cells | 3.23 | 0.208 |  | CASP7,ICAM1,LYVE1,SERPINB9,SRGN |
| Apelin Liver Signaling Pathway | 3.07 | 0.192 | 2.236 | APLNR,COL1A1,COL1A2,COL3A1,EDN1 |
| IL-15 Production | 3.04 | 0.0909 |  | AATK,CSF2RA,DDR2,EPHB2,ERBB2,FLT1,FLT4,JAK3,MET,MUSK,STYK1 |
| IL-10 Signaling | 3.03 | 0.116 |  | CD14,FCGR2A,HMOX1,IL1R1,IL4R,NFKBIA,SOCS3,STAT3 |
| Synaptogenesis Signaling Pathway | 2.99 | 0.0641 | -0.688 | ADCY2,APOE,CDH10,CDH12,CDH23,CDH5,CDH9,EFNA1,EPHB2,GRIN3A,GRM3,NLGN4Y,RASD1,SHC1,STX1A,SYN3,SYT1,SYT4,SYT6,THBS1 |
| Dendritic Cell Maturation | 2.96 | 0.0765 | 3.742 | COL1A1,COL1A2,COL3A1,DDR2,FCGR2A,FCGR3A/FCGR3B,ICAM1,NFKBIA,PLCD1,PLCE1,TLR2,TNFRSF11B,TNFRSF1A,TNFRSF1B |
| Caveolar-mediated Endocytosis Signaling | 2.87 | 0.11 |  | ACTA2,FLNA,FLNC,ITGA5,ITGA8,ITGA9,ITGB4,ITGB8 |
| Oncostatin M Signaling | 2.81 | 0.14 | 2.236 | JAK3,MT2A,OSMR,RASD1,SHC1,STAT3 |
| Neuroinflammation Signaling Pathway | 2.81 | 0.0633 | 1.291 | BACE2,CALB1,CALB2,CX3CR1,GABRA3,GABRA5,GJB1,GRIN3A,HMOX1,ICAM1,IL1R1,IRAK3,JAK3,MFGE8,SLC6A12,SOD2,TGFB2,TLR2,TNFRSF1A |
| Macropinocytosis Signaling | 2.75 | 0.105 | 0 | CD14,CSF1,ITGA5,ITGB4,ITGB8,MET,PRKCH,RASD1 |
| Clathrin-mediated Endocytosis Signaling | 2.74 | 0.0725 |  | ACTA2,APOD,APOE,EPHB2,FGF11,FGF2,IGF1,ITGA5,ITGB4,ITGB8,LDLR,MET,SERPINA1,VEGFA |
| GADD45 Signaling | 2.69 | 0.211 |  | CDKN1A,GADD45A,GADD45B,TP53 |
| Role of Osteoblasts, Osteoclasts and Chondrocytes in Rheumatoid Arthritis | 2.64 | 0.0682 |  | ALPL,COL1A1,CSF1,IGF1,IL1R1,ITGA5,NFKBIA,SFRP1,SPP1,TCF7L2,TNFRSF11B,TNFRSF1A,TNFRSF1B,WIF1,WNT5A |
| Phospholipases | 2.61 | 0.111 | 1.134 | HMOX1,PLA2G7,PLA2R1,PLAAT4,PLCD1,PLCE1,PLD4 |
| Pyridoxal 5'-phosphate Salvage Pathway | 2.53 | 0.108 | 1.633 | LIMK2,MAK,MAP3K8,PIM1,PRKCH,PRKX,SGK1 |
| G-Protein Coupled Receptor Signaling | 2.51 | 0.0625 |  | ADCY2,APLNR,CNR1,DRD3,GRM3,HTR2A,HTR2C,MAP3K8,NFKBIA,OPRK1,P2RY12,P2RY13,RASD1,RGS2,S1PR3,SHC1,STAT3 |
| Interferon Signaling | 2.41 | 0.139 | 2.236 | IFI6,IFITM1,IFITM2,IFITM3,TAP1 |
| Phagosome Formation | 2.41 | 0.08 |  | DIRAS3,FCGR2A,FCGR3A/FCGR3B,FN1,ITGA5,PLCD1,PLCE1,PRKCH,SCARA3,TLR2 |
| Role of JAK1 and JAK3 in γc Cytokine Signaling | 2.38 | 0.101 |  | BLNK,IL4R,JAK3,RASD1,SHC1,SOCS3,STAT3 |
| Apelin Cardiac Fibroblast Signaling Pathway | 2.37 | 0.174 | -1 | ANGPT2,APLNR,CCN2,TGFB2 |
| ILK Signaling | 2.37 | 0.0684 | 1.732 | ACTA2,DIRAS3,FLNA,FLNC,FN1,ITGB4,ITGB8,MYH11,MYL9,PXN,TNFRSF1A,VEGFA,VIM |
| Integrin Signaling | 2.35 | 0.0657 | 2.714 | ACTA2,DIRAS3,ITGA5,ITGA8,ITGA9,ITGB4,ITGB8,MYL9,MYLK3,NEDD9,PXN,RASD1,SHC1,ZYX |
| Glycogen Degradation II | 2.34 | 0.25 |  | PYGL,PYGM,TYMP |
| Agranulocyte Adhesion and Diapedesis | 2.33 | 0.0677 |  | ACTA2,CDH5,FN1,ICAM1,ICAM2,IL1R1,ITGA5,MYH11,MYL9,PECAM1,SELL,SELPLG,TNFRSF1A |
| Pancreatic Adenocarcinoma Signaling | 2.31 | 0.0826 | 2.121 | CDKN1A,ERBB2,HMOX1,JAK3,PLD4,STAT3,TGFB2,TP53,VEGFA |
| Melatonin Degradation II | 2.3 | 0.5 |  | MAOA,MAOB |
| Gα12/13 Signaling | 2.29 | 0.0769 | 1.414 | CDH10,CDH12,CDH23,CDH5,CDH9,LPAR3,MYL9,NFKBIA,PXN,RASD1 |
| Actin Cytoskeleton Signaling | 2.27 | 0.0642 | 2.828 | ACTA2,CD14,FGF11,FGF2,FLNA,FN1,ITGA5,LIMK2,MYH11,MYL9,MYLK3,PXN,RASD1,SHC1 |
| Death Receptor Signaling | 2.27 | 0.0879 | 0.707 | ACTA2,CASP7,HSPB1,NFKBIA,TIPARP,TNFRSF1A,TNFRSF1B,TNFSF10 |
| Cardiac Hypertrophy Signaling (Enhanced) | 2.27 | 0.0513 | 3.128 | ADCY2,EDN1,EDNRB,FGF11,FGF2,FGFRL1,HDAC7,HSPB1,IGF1,IL1R1,IL4R,ITGA5,MAP3K8,PDK1,PLCD1,PLCE1,PRKCH,RASD1,STAT3,TGFB2,TNFRSF11B,TNFRSF1A,TNFRSF1B,TNFSF10,WNT5A |
| PI3K/AKT Signaling | 2.25 | 0.069 | 2.121 | CDKN1A,GYS1,IL1R1,IL4R,ITGA5,JAK3,MAP3K8,MCL1,NFKBIA,RASD1,SHC1,TP53 |
| T Helper Cell Differentiation | 2.25 | 0.0959 |  | BCL6,IL4R,RORC,STAT3,TNFRSF11B,TNFRSF1A,TNFRSF1B |
| Adipogenesis pathway | 2.2 | 0.0746 |  | CEBPB,FGF2,FGFRL1,HDAC7,LPL,SOX9,TNFRSF1A,TP53,TXNIP,WNT5A |
| IL-8 Signaling | 2.19 | 0.065 | 1.732 | ANGPT2,DIRAS3,FLT1,FLT4,HMOX1,ICAM1,IRAK3,LIMK2,MYL9,PLD4,PRKCH,RASD1,VEGFA |
| Glycogen Degradation III | 2.14 | 0.214 |  | PYGL,PYGM,TYMP |
| Phenylalanine Degradation IV (Mammalian, via Side Chain) | 2.14 | 0.214 |  | MAOA,MAOB,SLC27A3 |
| Iron homeostasis signaling pathway | 2.13 | 0.073 |  | CD163,CP,HBA1/HBA2,HBB,HIF3A,HMOX1,JAK3,SLC39A14,STAT3,STEAP3 |
| Intrinsic Prothrombin Activation Pathway | 2.12 | 0.119 | 2.236 | COL1A1,COL1A2,COL3A1,F13A1,F5 |
| Bladder Cancer Signaling | 2.1 | 0.0825 |  | CDKN1A,ERBB2,FGF11,FGF2,RASD1,THBS1,TP53,VEGFA |
| Agrin Interactions at Neuromuscular Junction | 2.09 | 0.0897 | 2.236 | ACTA2,ERBB2,ITGA5,LAMA2,MUSK,PXN,RASD1 |
| Serotonin Receptor Signaling | 2.08 | 0.116 |  | ADCY2,HTR2A,HTR2C,MAOA,MAOB |
| Induction of Apoptosis by HIV1 | 2.05 | 0.0984 | 1.633 | NFKBIA,TNFRSF11B,TNFRSF1A,TNFRSF1B,TP53,TRAF1 |
| JAK/Stat Signaling | 2.03 | 0.0875 | 0.816 | CDKN1A,CEBPB,JAK3,RASD1,SHC1,SOCS3,STAT3 |
| Type II Diabetes Mellitus Signaling | 2.02 | 0.0704 | 0.816 | ACSL5,CEBPB,KCNJ11,NFKBIA,PRKCH,SLC27A3,SOCS3,TNFRSF11B,TNFRSF1A,TNFRSF1B |
| Phospholipase C Signaling | 2.02 | 0.0584 | 0.302 | ADCY2,BLNK,DIRAS3,FCGR2A,HDAC7,HMOX1,ITGA5,MYL9,PLCD1,PLCE1,PLD4,PRKCH,RASD1,SHC1,TGM2 |
| Production of Nitric Oxide and Reactive Oxygen Species in Macrophages | 2 | 0.0638 | 2.309 | APOD,APOE,DIRAS3,JAK3,MAP3K8,NFKBIA,PRKCH,SERPINA1,TLR2,TNFRSF11B,TNFRSF1A,TNFRSF1B |
| Extrinsic Prothrombin Activation Pathway | 1.97 | 0.188 |  | F13A1,F3,F5 |
| Dopamine Degradation | 1.95 | 0.133 | 2 | ALDH4A1,MAOA,MAOB,SULT1C4 |
| Xenobiotic Metabolism Signaling | 1.94 | 0.0557 |  | ALDH1L1,ALDH1L2,ALDH4A1,CAMK1G,CHST3,CYP1B1,HMOX1,MAOA,MAOB,MAP3K8,MGST1,NQO1,PRKCH,RASD1,SULT1C4,UGT8 |
| Regulation of the Epithelial-Mesenchymal Transition Pathway | 1.93 | 0.0625 |  | CDH12,FGF11,FGF2,FGFRL1,JAK3,LOX,MET,RASD1,STAT3,TCF7L2,TGFB2,WNT5A |
| HER-2 Signaling in Breast Cancer | 1.92 | 0.0833 |  | CDKN1A,ERBB2,ITGB4,ITGB8,PRKCH,RASD1,TP53 |
| Glycogen Biosynthesis II (from UDP-D-Glucose) | 1.92 | 0.333 |  | GYG2,GYS1 |
| Germ Cell-Sertoli Cell Junction Signaling | 1.9 | 0.0643 |  | A2M,ACTA2,DIRAS3,LIMK2,MAP3K8,PXN,RASD1,TGFB2,TNFRSF1A,TUBA1C,ZYX |
| Virus Entry via Endocytic Pathways | 1.86 | 0.0748 |  | ACTA2,FLNA,FLNC,ITGA5,ITGB4,ITGB8,PRKCH,RASD1 |
| Paxillin Signaling | 1.84 | 0.0741 |  | ACTA2,ITGA5,ITGA8,ITGA9,ITGB4,ITGB8,PXN,RASD1 |
| Gap Junction Signaling | 1.83 | 0.0606 |  | ACTA2,ADCY2,CCN3,GJB1,GJC2,HTR2A,HTR2C,PLCD1,PLCE1,PRKCH,RASD1,TUBA1C |
| SPINK1 General Cancer Pathway | 1.8 | 0.087 | -0.447 | JAK3,MT1M,MT1X,MT2A,RASD1,STAT3 |
| Glioma Signaling | 1.79 | 0.0727 | 0.447 | CAMK1G,CDKN1A,IGF1,IGF2,PRKCH,RASD1,SHC1,TP53 |
| Thyroid Cancer Signaling | 1.77 | 0.098 |  | RASD1,RXRG,SHC1,TCF7L2,TP53 |
| HGF Signaling | 1.77 | 0.0721 | 0.378 | CDKN1A,ITGA5,MAP3K8,MET,PRKCH,PXN,RASD1,STAT3 |
| Inhibition of Angiogenesis by TSP1 | 1.76 | 0.118 |  | HSPG2,THBS1,TP53,VEGFA |
| Granulocyte Adhesion and Diapedesis | 1.76 | 0.0615 |  | CDH5,ICAM1,ICAM2,IL1R1,ITGA5,PECAM1,SELL,SELPLG,TNFRSF11B,TNFRSF1A,TNFRSF1B |
| NF-κB Signaling | 1.76 | 0.0615 | 1.897 | FLT1,FLT4,IL1R1,IRAK3,MAP3K8,NFKBIA,RASD1,TLR2,TNFRSF11B,TNFRSF1A,TNFRSF1B |
| Axonal Guidance Signaling | 1.75 | 0.0474 |  | ADAMTS8,ADAMTS9,ECEL1,EFNA1,EPHB2,ERBB2,IGF1,ITGA5,LIMK2,MET,MYL9,PLCD1,PLCE1,PRKCH,PXN,RASD1,SEMA3E,SEMA3G,SEMA4B,SHC1,TUBA1C,VEGFA,WNT5A |
| RhoGDI Signaling | 1.74 | 0.0611 | -0.905 | ACTA2,CD44,CDH10,CDH12,CDH23,CDH5,CDH9,DIRAS3,ITGA5,LIMK2,MYL9 |
| Growth Hormone Signaling | 1.74 | 0.0845 | -1 | A2M,IGF1,IGF2,PRKCH,SOCS3,STAT3 |
| cAMP-mediated signaling | 1.74 | 0.057 | 0 | ADCY2,AKAP5,APLNR,CAMK1G,CNR1,DRD3,GRM3,OPRK1,P2RY12,P2RY13,RGS2,S1PR3,STAT3 |
| Hepatic Cholestasis | 1.68 | 0.0598 |  | ADCY2,CD14,IL1R1,IRAK3,NFKBIA,PRKCH,TGFB2,TNFRSF11B,TNFRSF1A,TNFRSF1B,TNFSF10 |
| Fcγ Receptor-mediated Phagocytosis in Macrophages and Monocytes | 1.68 | 0.0745 | 1.134 | ACTA2,FCGR2A,FCGR3A/FCGR3B,HMOX1,PLD4,PRKCH,PXN |
| Neuroprotective Role of THOP1 in Alzheimer's Disease | 1.67 | 0.069 | 0.447 | C1R,ECE1,NTS,PDYN,PRSS12,PRSS23,SERPINA3,TAC1 |
| Superoxide Radicals Degradation | 1.67 | 0.25 |  | NQO1,SOD2 |
| Role of Tissue Factor in Cancer | 1.65 | 0.0684 |  | CCN1,CCN2,CSF1,F3,LIMK2,RASD1,TP53,VEGFA |
| Putrescine Degradation III | 1.64 | 0.143 |  | ALDH4A1,MAOA,MAOB |
| Amyotrophic Lateral Sclerosis Signaling | 1.61 | 0.0722 | 1.633 | CASP7,GRIN3A,IGF1,NEFM,RNF19A,TP53,VEGFA |
| Salvage Pathways of Pyrimidine Ribonucleotides | 1.61 | 0.0722 | 1.633 | LIMK2,MAK,MAP3K8,PIM1,PRKCH,PRKX,SGK1 |
| Glioblastoma Multiforme Signaling | 1.61 | 0.0606 | 0.333 | CDKN1A,DIRAS3,IGF1,IGF2,PLCD1,PLCE1,RASD1,SHC1,TP53,WNT5A |
| NRF2-mediated Oxidative Stress Response | 1.61 | 0.0582 |  | ACTA2,DNAJB1,ENC1,FKBP5,HMOX1,HSPB8,MGST1,NQO1,PRKCH,RASD1,SOD2 |
| Endocannabinoid Cancer Inhibition Pathway | 1.59 | 0.0629 | -0.333 | ADCY2,ATF3,CASP7,CDKN1A,CNR1,NUPR1,TCF7L2,VEGFA,VIM |
| Apoptosis Signaling | 1.57 | 0.0707 | 1.633 | CASP7,MCL1,NFKBIA,RASD1,TNFRSF1A,TNFRSF1B,TP53 |
| VEGF Signaling | 1.57 | 0.0707 | 2.449 | ACTA2,FLT1,FLT4,PXN,RASD1,SHC1,VEGFA |
| Tight Junction Signaling | 1.56 | 0.0595 |  | ACTA2,F11R,MYH11,MYL9,RAB13,TGFB2,TNFRSF11B,TNFRSF1A,TNFRSF1B,YBX3 |
| RhoA Signaling | 1.53 | 0.065 | 1.414 | ACTA2,IGF1,LIMK2,LPAR3,MYL9,MYLK3,RHPN2,SEPTIN9 |
| Neuropathic Pain Signaling In Dorsal Horn Neurons | 1.53 | 0.0693 | -0.378 | CAMK1G,GRIN3A,GRM3,PLCD1,PLCE1,PRKCH,TAC1 |
| Leukocyte Extravasation Signaling | 1.49 | 0.0558 | 1.265 | ACTA2,CD44,CDH5,F11R,ICAM1,ITGA5,PECAM1,PRKCH,PXN,SELPLG,TIMP1 |
| Adrenomedullin signaling pathway | 1.49 | 0.0558 | 2.333 | ADCY2,ADM,CEBPB,CFH,MYLK3,PLCD1,PLCE1,RAMP3,RASD1,RXRG,SHC1 |
| Prolactin Signaling | 1.49 | 0.0741 | 0 | CEBPB,PRKCH,RASD1,SHC1,SOCS3,STAT3 |
| Wnt/β-catenin Signaling | 1.49 | 0.0578 | 1.414 | CD44,CDH12,CDH5,SFRP1,SOX9,TCF7L2,TGFB2,TP53,WIF1,WNT5A |
| PEDF Signaling | 1.47 | 0.0732 | 1 | CASP7,NFKBIA,RASD1,SOD2,TCF7L2,TP53 |
| PPAR Signaling | 1.47 | 0.0673 | -2.449 | IL1R1,NFKBIA,RASD1,SHC1,TNFRSF11B,TNFRSF1A,TNFRSF1B |
| Role of JAK family kinases in IL-6-type Cytokine Signaling | 1.44 | 0.12 |  | OSMR,SOCS3,STAT3 |
| Tryptophan Degradation X (Mammalian, via Tryptamine) | 1.44 | 0.12 |  | ALDH4A1,MAOA,MAOB |
| Epithelial Adherens Junction Signaling | 1.44 | 0.0592 |  | ACTA2,MET,MYH11,MYL9,RASD1,TCF7L2,TGFB2,TUBA1C,ZYX |
| BAG2 Signaling Pathway | 1.43 | 0.093 | 2 | ANXA2,CDKN1A,HSPA1A/HSPA1B,TP53 |
| White Adipose Tissue Browning Pathway | 1.43 | 0.062 | 1.414 | ADCY2,ANGPT2,CEBPB,FGFRL1,NDN,RXRG,VEGFA,VGF |
| VEGF Family Ligand-Receptor Interactions | 1.42 | 0.0714 | 1.342 | FLT1,FLT4,PRKCH,RASD1,SHC1,VEGFA |
| IL-23 Signaling Pathway | 1.4 | 0.0909 | 0 | NFKBIA,RORC,SOCS3,STAT3 |
| Ephrin Receptor Signaling | 1.39 | 0.0556 | 1.633 | EFNA1,EPHB2,GRIN3A,ITGA5,LIMK2,PXN,RASD1,SHC1,STAT3,VEGFA |
| Glucocorticoid Receptor Signaling | 1.38 | 0.0476 |  | A2M,ANXA1,CD163,CDKN1A,CEBPB,FKBP5,HSPA1A/HSPA1B,ICAM1,JAK3,NFKBIA,RASD1,SGK1,SHC1,STAT3,TGFB2,TSC22D3 |
| IL-12 Signaling and Production in Macrophages | 1.38 | 0.0606 |  | APOD,APOE,CEBPB,MAP3K8,PRKCH,SERPINA1,TGFB2,TLR2 |
| CD40 Signaling | 1.37 | 0.0769 | 1.342 | ICAM1,JAK3,NFKBIA,STAT3,TRAF1 |
| ErbB2-ErbB3 Signaling | 1.37 | 0.0769 | 2 | ERBB2,JAK3,RASD1,SHC1,STAT3 |
| iNOS Signaling | 1.37 | 0.0889 | 2 | CD14,IRAK3,JAK3,NFKBIA |
| Gαq Signaling | 1.36 | 0.0573 | -1.414 | DIRAS3,GYS1,HMOX1,HTR2A,HTR2C,NFKBIA,PLD4,PRKCH,RGS2 |
| PFKFB4 Signaling Pathway | 1.34 | 0.087 | 1 | FGF2,HK2,TGFB2,TP53 |
| Huntington's Disease Signaling | 1.31 | 0.0506 | -0.378 | CASP7,DNAJB1,HDAC7,HSPA1A/HSPA1B,IGF1,PENK,PRKCH,SGK1,SHC1,STX1A,TGM2,TP53 |
| Th17 Activation Pathway | 1.28 | 0.0659 | 2.449 | IL1R1,IRAK3,JAK3,RORC,SOCS3,STAT3 |
| Role of NFAT in Cardiac Hypertrophy | 1.28 | 0.0514 | 1.667 | ADCY2,AKAP5,CAMK1G,HDAC7,IGF1,PLCD1,PLCE1,PRKCH,RASD1,SHC1,TGFB2 |
| Fatty Acid Activation | 1.26 | 0.154 |  | ACSL5,SLC27A3 |
| GM-CSF Signaling | 1.26 | 0.0714 |  | CSF2RA,PIM1,RASD1,SHC1,STAT3 |
| PPARα/RXRα Activation | 1.26 | 0.0526 | -0.816 | ADCY2,GPD1,IL1R1,LPL,NFKBIA,PLCD1,PLCE1,RASD1,SHC1,TGFB2 |
| HMGB1 Signaling | 1.25 | 0.0545 | 1.134 | DIRAS3,ICAM1,IL1R1,RASD1,TGFB2,TNFRSF11B,TNFRSF1A,TNFRSF1B,TNFSF10 |
| TNFR2 Signaling | 1.24 | 0.1 |  | NFKBIA,TNFRSF1B,TRAF1 |
| 4-hydroxyproline Degradation I | 1.24 | 0.5 |  | ALDH4A1 |
| Sulfate Activation for Sulfonation | 1.24 | 0.5 |  | PAPSS2 |
| Signaling by Rho Family GTPases | 1.23 | 0.0492 | 1.155 | ACTA2,CDH10,CDH12,CDH23,CDH5,CDH9,DIRAS3,ITGA5,LIMK2,MYL9,SEPTIN9,VIM |
| p38 MAPK Signaling | 1.22 | 0.0593 | 2.449 | HSPB1,IL1R1,IRAK3,TGFB2,TNFRSF1A,TNFRSF1B,TP53 |
| α-Adrenergic Signaling | 1.21 | 0.0632 |  | ADCY2,GYS1,PRKCH,PYGL,PYGM,RASD1 |
| Opioid Signaling Pathway | 1.2 | 0.0486 | -0.905 | ADCY2,CAMK1G,GRIN3A,NFKBIA,OPRK1,PDK1,PDYN,PENK,PRKCH,RASD1,RGS1,RGS8 |
| Neuregulin Signaling | 1.19 | 0.0625 | 1 | DCN,ERBB2,ITGA5,PRKCH,RASD1,SHC1 |
| Senescence Pathway | 1.19 | 0.0473 | 1.941 | ATF3,CDKN1A,CEBPB,GADD45A,GADD45B,PDK1,PDK4,RASD1,SOD2,TGFB2,TLR2,TP53,ZFP36L1 |
| PAK Signaling | 1.17 | 0.0619 | 2.236 | ITGA5,LIMK2,MYL9,PXN,RASD1,SHC1 |
| Leptin Signaling in Obesity | 1.17 | 0.0676 |  | ADCY2,PLCD1,PLCE1,SOCS3,STAT3 |
| Hypoxia Signaling in the Cardiovascular System | 1.17 | 0.0676 | 1 | EDN1,NFKBIA,NQO1,TP53,VEGFA |
| TREM1 Signaling | 1.15 | 0.0667 | 2.236 | ICAM1,ITGA5,NLRC5,STAT3,TLR2 |
| Choline Biosynthesis III | 1.15 | 0.133 |  | HMOX1,PLD4 |
| IL-9 Signaling | 1.14 | 0.0909 |  | JAK3,SOCS3,STAT3 |
| Colorectal Cancer Metastasis Signaling | 1.14 | 0.0474 | 1.897 | ADCY2,DIRAS3,JAK3,RASD1,STAT3,TCF7L2,TGFB2,TLR2,TNFRSF1A,TP53,VEGFA,WNT5A |
| Erythropoietin Signaling | 1.13 | 0.0658 |  | NFKBIA,PRKCH,RASD1,SHC1,SOCS3 |
| Toll-like Receptor Signaling | 1.13 | 0.0658 | 2 | CD14,IRAK3,NFKBIA,TLR2,TRAF1 |
| Role of JAK2 in Hormone-like Cytokine Signaling | 1.11 | 0.0882 |  | SHC1,SOCS3,STAT3 |
| IL-7 Signaling Pathway | 1.1 | 0.0641 | 1 | BCL6,JAK3,MCL1,MET,SHC1 |
| 14-3-3-mediated Signaling | 1.09 | 0.0551 | 1.342 | PLCD1,PLCE1,PRKCH,RASD1,TNFRSF1A,TUBA1C,VIM |
| P2Y Purigenic Receptor Signaling Pathway | 1.09 | 0.0551 | 0.816 | ADCY2,P2RY1,P2RY12,PLCD1,PLCE1,PRKCH,RASD1 |
| Protein Kinase A Signaling | 1.09 | 0.0427 | 0.333 | ADCY2,AKAP5,FLNA,FLNC,GYS1,MYL9,MYLK3,NFKBIA,PLCD1,PLCE1,PRKCH,PTPRU,PXN,PYGL,PYGM,TCF7L2,TGFB2 |
| TWEAK Signaling | 1.08 | 0.0857 |  | CASP7,NFKBIA,TRAF1 |
| Noradrenaline and Adrenaline Degradation | 1.08 | 0.0857 |  | ALDH4A1,MAOA,MAOB |
| Proline Degradation | 1.07 | 0.333 |  | ALDH4A1 |
| Glycerol-3-phosphate Shuttle | 1.07 | 0.333 |  | GPD1 |
| γ-linolenate Biosynthesis II (Animals) | 1.06 | 0.118 |  | ACSL5,SLC27A3 |
| Mitochondrial L-carnitine Shuttle Pathway | 1.06 | 0.118 |  | ACSL5,SLC27A3 |
| Cancer Drug Resistance By Drug Efflux | 1.04 | 0.069 |  | ABCG2,PDK1,RASD1,TP53 |
| Aldosterone Signaling in Epithelial Cells | 1.02 | 0.0506 | 1 | DNAJB1,HSPA1A/HSPA1B,HSPB1,HSPB8,PLCD1,PLCE1,PRKCH,SGK1 |
| 1D-myo-inositol Hexakisphosphate Biosynthesis II (Mammalian) | 1.01 | 0.111 |  | ITPKB,ITPKC |
| D-myo-inositol (1,3,4)-trisphosphate Biosynthesis | 1.01 | 0.111 |  | ITPKB,ITPKC |
| Sertoli Cell-Sertoli Cell Junction Signaling | 1.01 | 0.0486 |  | A2M,ACTA2,F11R,ITGA5,MAP3K8,RASD1,TNFRSF1A,TUBA1C,YBX3 |
| eNOS Signaling | 1.01 | 0.0503 | 1.134 | ADCY2,AQP1,FLT1,FLT4,HSPA1A/HSPA1B,LPAR3,PRKCH,VEGFA |
| FGF Signaling | 0.991 | 0.0595 | 1.342 | FGF11,FGF2,FGFRL1,MET,STAT3 |
| Synaptic Long Term Depression | 0.971 | 0.0476 | 0 | GRM3,IGF1,PLA2G7,PLA2R1,PLAAT4,PLCD1,PLCE1,PRKCH,RASD1 |
| Inhibition of Matrix Metalloproteases | 0.971 | 0.0769 |  | A2M,HSPG2,TIMP1 |
| Type I Diabetes Mellitus Signaling | 0.959 | 0.0541 | 1.633 | IL1R1,NFKBIA,SOCS3,TNFRSF11B,TNFRSF1A,TNFRSF1B |
| Heme Degradation | 0.947 | 0.25 |  | HMOX1 |
| Ascorbate Recycling (Cytosolic) | 0.947 | 0.25 |  | GLRX |
| α-tocopherol Degradation | 0.947 | 0.25 |  | CYP4F3 |
| Glutathione Redox Reactions II | 0.947 | 0.25 |  | GLRX |
| Arginine Degradation I (Arginase Pathway) | 0.947 | 0.25 |  | ALDH4A1 |
| PI3K Signaling in B Lymphocytes | 0.943 | 0.0507 | 1.633 | ATF3,BLNK,IL4R,NFKBIA,PLCD1,PLCE1,RASD1 |
| Thrombopoietin Signaling | 0.939 | 0.0635 |  | PRKCH,RASD1,SHC1,STAT3 |
| Ovarian Cancer Signaling | 0.932 | 0.0504 |  | CD44,EDN1,RASD1,TCF7L2,TP53,VEGFA,WNT5A |
| Ceramide Signaling | 0.928 | 0.0568 | 2 | RASD1,S1PR3,TNFRSF11B,TNFRSF1A,TNFRSF1B |
| Role of PKR in Interferon Induction and Antiviral Response | 0.924 | 0.0732 |  | NFKBIA,TNFRSF1A,TP53 |
| Acute Myeloid Leukemia Signaling | 0.914 | 0.0562 |  | CSF2RA,PIM1,RASD1,STAT3,TCF7L2 |
| Myc Mediated Apoptosis Signaling | 0.903 | 0.0615 |  | IGF1,RASD1,SHC1,TP53 |
| Superpathway of Melatonin Degradation | 0.903 | 0.0615 | 2 | CYP1B1,MAOA,MAOB,SULT1C4 |
| MIF Regulation of Innate Immunity | 0.9 | 0.0714 |  | CD14,NFKBIA,TP53 |
| Sphingosine-1-phosphate Signaling | 0.879 | 0.0513 | 0 | ADCY2,CASP7,DIRAS3,PLCD1,PLCE1,S1PR3 |
| Serotonin Degradation | 0.87 | 0.0597 | 2 | ALDH4A1,MAOA,MAOB,SULT1C4 |
| Cell Cycle: G1/S Checkpoint Regulation | 0.87 | 0.0597 |  | CDKN1A,HDAC7,TGFB2,TP53 |
| Breast Cancer Regulation by Stathmin1 | 0.86 | 0.045 |  | ADCY2,CAMK1G,CDKN1A,LIMK2,PRKCH,RASD1,SHC1,TP53,TUBA1C |
| Tetrahydrofolate Salvage from 5,10-methenyltetrahydrofolate | 0.857 | 0.2 |  | MTHFD2 |
| Role of NANOG in Mammalian Embryonic Stem Cell Pluripotency | 0.854 | 0.0504 | 1 | JAK3,RASD1,SHC1,STAT3,TP53,WNT5A |
| Remodeling of Epithelial Adherens Junctions | 0.854 | 0.0588 |  | ACTA2,MET,TUBA1C,ZYX |
| Superpathway of D-myo-inositol (1,4,5)-trisphosphate Metabolism | 0.836 | 0.087 |  | ITPKB,ITPKC |
| FAK Signaling | 0.827 | 0.0526 |  | ACTA2,ITGA5,PXN,RASD1,TNS1 |
| GABA Receptor Signaling | 0.827 | 0.0526 |  | ADCY2,GABRA3,GABRA5,SLC32A1,SLC6A12 |
| Inhibition of ARE-Mediated mRNA Degradation Pathway | 0.818 | 0.0492 | 0 | TNFRSF11B,TNFRSF1A,TNFRSF1B,TNFSF10,ZFP36,ZFP36L1 |
| IL-22 Signaling | 0.804 | 0.0833 |  | SOCS3,STAT3 |
| IL-15 Signaling | 0.804 | 0.0563 |  | JAK3,RASD1,SHC1,STAT3 |
| Small Cell Lung Cancer Signaling | 0.804 | 0.0563 |  | NFKBIA,RXRG,TP53,TRAF1 |
| ATM Signaling | 0.801 | 0.0515 |  | CDKN1A,GADD45A,GADD45B,NFKBIA,TP53 |
| nNOS Signaling in Neurons | 0.796 | 0.0638 |  | GRIN3A,PRKCH,RASD1 |
| Melatonin Signaling | 0.79 | 0.0556 | 1 | PLCD1,PLCE1,PRKCH,RORC |
| Thrombin Signaling | 0.79 | 0.0433 | 0.378 | ADCY2,CAMK1G,DIRAS3,MYL9,PLCD1,PLCE1,PRKCH,RASD1,SHC1 |
| p53 Signaling | 0.788 | 0.051 |  | CDKN1A,GADD45A,GADD45B,THBS1,TP53 |
| UVA-Induced MAPK Signaling | 0.788 | 0.051 |  | PLCD1,PLCE1,RASD1,TIPARP,TP53 |
| Ceramide Biosynthesis | 0.785 | 0.167 |  | SPTSSA |
| Glycerol Degradation I | 0.785 | 0.167 |  | GPD1 |
| NAD Biosynthesis III | 0.785 | 0.167 |  | NAMPT |
| D-myo-inositol (1,4,5)-Trisphosphate Biosynthesis | 0.777 | 0.08 |  | PLCD1,PLCE1 |
| FXR/RXR Activation | 0.775 | 0.0476 |  | APOD,APOE,C4A/C4B,LPL,SERPINA1,TTR |
| Non-Small Cell Lung Cancer Signaling | 0.775 | 0.0548 |  | ERBB2,RASD1,RXRG,TP53 |
| PTEN Signaling | 0.775 | 0.0476 | -1.342 | CDKN1A,FLT1,FLT4,ITGA5,RASD1,SHC1 |
| Apelin Cardiomyocyte Signaling Pathway | 0.775 | 0.0505 | 1.342 | APLNR,MYL9,PLCD1,PLCE1,PRKCH |
| Role of Pattern Recognition Receptors in Recognition of Bacteria and Viruses | 0.77 | 0.0455 | 1.342 | C1QA,C1QB,C1QC,PRKCH,TGFB2,TLR2,TNFSF10 |
| Cardiac Hypertrophy Signaling | 0.764 | 0.0417 | 1.667 | ADCY2,DIRAS3,HSPB1,IGF1,MAP3K8,MYL9,PLCD1,PLCE1,RASD1,TGFB2 |
| Glioma Invasiveness Signaling | 0.759 | 0.0541 |  | CD44,DIRAS3,RASD1,TIMP1 |
| Cell Cycle: G2/M DNA Damage Checkpoint Regulation | 0.757 | 0.0612 |  | CDKN1A,GADD45A,TP53 |
| Antiproliferative Role of TOB in T Cell Signaling | 0.75 | 0.0769 |  | TGFB2,TOB1 |
| Synaptic Long Term Potentiation | 0.742 | 0.0465 | -0.447 | GRIN3A,GRM3,PLCD1,PLCE1,PRKCH,RASD1 |
| Reelin Signaling in Neurons | 0.742 | 0.0465 | 0.447 | APOE,CNR1,GRIN3A,ITGA5,PDK1,PDK4 |
| p70S6K Signaling | 0.742 | 0.0465 | 1.342 | IL4R,PLCD1,PLCE1,PRKCH,RASD1,SHC1 |
| Melanoma Signaling | 0.74 | 0.06 |  | CDKN1A,RASD1,TP53 |
| Primary Immunodeficiency Signaling | 0.74 | 0.06 |  | BLNK,JAK3,TAP1 |
| TNFR1 Signaling | 0.74 | 0.06 |  | CASP7,NFKBIA,TNFRSF1A |
| Chronic Myeloid Leukemia Signaling | 0.726 | 0.0485 |  | CDKN1A,HDAC7,RASD1,TGFB2,TP53 |
| Mouse Embryonic Stem Cell Pluripotency | 0.726 | 0.0485 | 1 | JAK3,RASD1,STAT3,TCF7L2,TP53 |
| Cellular Effects of Sildenafil (Viagra) | 0.721 | 0.0458 |  | ACTA2,ADCY2,MYH11,MYL9,PLCD1,PLCE1 |
| UVC-Induced MAPK Signaling | 0.721 | 0.0588 |  | PRKCH,RASD1,TP53 |
| Systemic Lupus Erythematosus In B Cell Signaling Pathway | 0.721 | 0.04 | 0.905 | BLNK,FCGR2A,MCL1,PLAAT4,PRKCH,RASD1,SHC1,STAT3,TGFB2,TNFSF10,TRAF1 |
| Dopamine Receptor Signaling | 0.717 | 0.0519 |  | ADCY2,DRD3,MAOA,MAOB |
| PD-1, PD-L1 cancer immunotherapy pathway | 0.693 | 0.0472 | 1 | JAK3,TGFB2,TNFRSF11B,TNFRSF1A,TNFRSF1B |
| IL-3 Signaling | 0.69 | 0.0506 |  | PRKCH,RASD1,SHC1,STAT3 |
| Dopamine-DARPP32 Feedback in cAMP Signaling | 0.686 | 0.0429 | 0.447 | ADCY2,DRD3,GRIN3A,KCNJ11,PLCD1,PLCE1,PRKCH |
| Human Embryonic Stem Cell Pluripotency | 0.682 | 0.0444 |  | FGF2,FGFRL1,S1PR3,TCF7L2,TGFB2,WNT5A |
| Telomerase Signaling | 0.682 | 0.0467 |  | CDKN1A,HDAC7,RASD1,SHC1,TP53 |
| Role of MAPK Signaling in the Pathogenesis of Influenza | 0.678 | 0.05 |  | PLA2G7,PLA2R1,PLAAT4,RASD1 |
| Tec Kinase Signaling | 0.678 | 0.0427 | 0.816 | ACTA2,DIRAS3,ITGA5,JAK3,PRKCH,STAT3,TNFSF10 |
| Histidine Degradation III | 0.672 | 0.125 |  | MTHFD2 |
| Salvage Pathways of Pyrimidine Deoxyribonucleotides | 0.672 | 0.125 |  | TYMP |
| Sphingomyelin Metabolism | 0.672 | 0.125 |  | SGMS2 |
| Cyclins and Cell Cycle Regulation | 0.666 | 0.0494 |  | CDKN1A,HDAC7,TGFB2,TP53 |
| LPS-stimulated MAPK Signaling | 0.654 | 0.0488 |  | CD14,NFKBIA,PRKCH,RASD1 |
| NF-κB Activation by Viruses | 0.654 | 0.0488 |  | ITGA5,NFKBIA,PRKCH,RASD1 |
| Insulin Receptor Signaling | 0.644 | 0.0432 | 1 | GYS1,RASD1,SGK1,SHC1,SOCS3,TRIP10 |
| Unfolded protein response | 0.642 | 0.0536 |  | CEBPB,HSPA1A/HSPA1B,PPP1R15A |
| Hereditary Breast Cancer Signaling | 0.635 | 0.0429 |  | CDKN1A,GADD45A,GADD45B,HDAC7,RASD1,TP53 |
| TR/RXR Activation | 0.629 | 0.0476 |  | COL6A3,KLF9,LDLR,RXRG |
| Folate Transformations I | 0.627 | 0.111 |  | MTHFD2 |
| Glutamate Receptor Signaling | 0.627 | 0.0526 |  | GRIN3A,GRM3,HOMER1 |
| HIF1α Signaling | 0.62 | 0.0442 |  | EDN1,RASD1,SLC2A5,TP53,VEGFA |
| IL-4 Signaling | 0.618 | 0.0471 |  | IL4R,JAK3,RASD1,SHC1 |
| 4-1BB Signaling in T Lymphocytes | 0.614 | 0.0625 |  | NFKBIA,TRAF1 |
| Fatty Acid β-oxidation I | 0.614 | 0.0625 |  | ACSL5,SLC27A3 |
| MSP-RON Signaling Pathway | 0.613 | 0.0517 |  | ACTA2,CSF1,TLR2 |
| PDGF Signaling | 0.606 | 0.0465 |  | JAK3,RASD1,SHC1,STAT3 |
| Apelin Endothelial Signaling Pathway | 0.6 | 0.0435 | 0 | ADCY2,APLNR,ICAM1,PRKCH,RASD1 |
| Semaphorin Signaling in Neurons | 0.585 | 0.05 |  | DIRAS3,LIMK2,MET |
| Endometrial Cancer Signaling | 0.585 | 0.05 |  | ERBB2,RASD1,TP53 |
| Retinoic acid Mediated Apoptosis Signaling | 0.585 | 0.05 |  | RXRG,TIPARP,TNFSF10 |
| Calcium Signaling | 0.58 | 0.0388 |  | ACTA2,AKAP5,CAMK1G,GRIN3A,HDAC7,MYH11,MYL9,TRPC4 |
| MIF-mediated Glucocorticoid Regulation | 0.577 | 0.0588 |  | CD14,NFKBIA |
| Regulation of IL-2 Expression in Activated and Anergic T Lymphocytes | 0.573 | 0.0449 |  | NFKBIA,RASD1,TGFB2,TOB1 |
| Renin-Angiotensin Signaling | 0.572 | 0.0424 |  | ADCY2,PRKCH,RASD1,SHC1,STAT3 |
| IL-2 Signaling | 0.572 | 0.0492 |  | JAK3,RASD1,SHC1 |
| Natural Killer Cell Signaling | 0.562 | 0.042 |  | FCGR2A,FCGR3A/FCGR3B,PRKCH,RASD1,SHC1 |
| Cholecystokinin/Gastrin-mediated Signaling | 0.562 | 0.042 | 0 | DIRAS3,PRKCH,PXN,RASD1,SHC1 |
| IL-17A Signaling in Fibroblasts | 0.559 | 0.0571 |  | CEBPB,NFKBIA |
| Wnt/Ca+ pathway | 0.559 | 0.0484 |  | PLCD1,PLCE1,WNT5A |
| Prostate Cancer Signaling | 0.553 | 0.044 |  | CDKN1A,NFKBIA,RASD1,TP53 |
| IL-1 Signaling | 0.553 | 0.044 |  | ADCY2,IL1R1,IRAK3,NFKBIA |
| γ-glutamyl Cycle | 0.553 | 0.0909 |  | GGT5 |
| Th1 Pathway | 0.545 | 0.0413 | 1 | ICAM1,JAK3,NFIL3,SOCS3,STAT3 |
| G Beta Gamma Signaling | 0.536 | 0.041 | 1 | ADCY2,CAV2,PRKCH,RASD1,SHC1 |
| IL-17A Signaling in Airway Cells | 0.535 | 0.0469 |  | JAK3,NFKBIA,STAT3 |
| Regulation of Actin-based Motility by Rho | 0.523 | 0.0426 | 1 | ACTA2,DIRAS3,ITGA5,MYL9 |
| ErbB Signaling | 0.523 | 0.0426 |  | ERBB2,PRKCH,RASD1,SHC1 |
| Regulation of Cellular Mechanics by Calpain Protease | 0.523 | 0.0462 |  | ITGA5,PXN,RASD1 |
| Hematopoiesis from Multipotent Stem Cells | 0.52 | 0.0833 |  | CSF1 |
| Guanosine Nucleotides Degradation III | 0.52 | 0.0833 |  | GDA |
| B Cell Receptor Signaling | 0.517 | 0.0378 | 1.342 | BCL6,BLNK,FCGR2A,MAP3K8,NFKBIA,RASD1,SHC1 |
| Eicosanoid Signaling | 0.511 | 0.0455 |  | PLA2G7,PLA2R1,PLAAT4 |
| ErbB4 Signaling | 0.5 | 0.0448 |  | PRKCH,RASD1,SHC1 |
| April Mediated Signaling | 0.495 | 0.0513 |  | NFKBIA,TRAF1 |
| Antigen Presentation Pathway | 0.495 | 0.0513 |  | NLRC5,TAP1 |
| Leukotriene Biosynthesis | 0.492 | 0.0769 |  | GGT5 |
| Pregnenolone Biosynthesis | 0.492 | 0.0769 |  | CYP4F11 |
| Endocannabinoid Neuronal Synapse Pathway | 0.488 | 0.0391 | -0.447 | ADCY2,CNR1,GRIN3A,PLCD1,PLCE1 |
| Nitric Oxide Signaling in the Cardiovascular System | 0.476 | 0.0404 | 1 | FLT1,FLT4,PRKCH,VEGFA |
| B Cell Activating Factor Signaling | 0.467 | 0.0488 |  | NFKBIA,TRAF1 |
| Mechanisms of Viral Exit from Host Cells | 0.467 | 0.0488 |  | ACTA2,PRKCH |
| RAR Activation | 0.466 | 0.0363 |  | ADCY2,DHRS3,PRKCH,RXRG,TGFB2,VEGFA,ZBTB16 |
| Retinol Biosynthesis | 0.453 | 0.0476 |  | DHRS3,LPL |
| SAPK/JNK Signaling | 0.451 | 0.0392 |  | GADD45A,RASD1,SHC1,TP53 |
| Sirtuin Signaling Pathway | 0.45 | 0.0344 | 1.897 | GADD45A,GADD45B,NAMPT,NQO1,PDK1,SOD2,STAT3,TP53,TUBA1C,TUBA4B |
| Basal Cell Carcinoma Signaling | 0.449 | 0.0417 |  | TCF7L2,TP53,WNT5A |
| ERK5 Signaling | 0.449 | 0.0417 |  | MAP3K8,RASD1,SGK1 |
| Actin Nucleation by ARP-WASP Complex | 0.449 | 0.0417 |  | DIRAS3,ITGA5,RASD1 |
| Superpathway of Inositol Phosphate Compounds | 0.442 | 0.0355 | 1.89 | ALPL,ITPKB,ITPKC,NUDT11,PLCD1,PLCE1,SOCS3 |
| Histidine Degradation VI | 0.442 | 0.0667 |  | CYP4F11 |
| GPCR-Mediated Integration of Enteroendocrine Signaling Exemplified by an L Cell | 0.438 | 0.0411 |  | ADCY2,PLCD1,PLCE1 |
| Molecular Mechanisms of Cancer | 0.434 | 0.0332 |  | ADCY2,CASP7,CDKN1A,DIRAS3,ITGA5,JAK3,NFKBIA,PRKCH,RASD1,SHC1,TGFB2,TP53,WNT5A |
| CXCR4 Signaling | 0.433 | 0.0359 | -0.447 | ADCY2,DIRAS3,MYL9,PRKCH,PXN,RASD1 |
| Vitamin-C Transport | 0.42 | 0.0625 |  | GLRX |
| FcγRIIB Signaling in B Lymphocytes | 0.419 | 0.04 |  | BLNK,RASD1,SHC1 |
| Angiopoietin Signaling | 0.419 | 0.04 |  | ANGPT2,NFKBIA,RASD1 |
| Th1 and Th2 Activation Pathway | 0.408 | 0.0351 |  | ICAM1,IL4R,JAK3,NFIL3,SOCS3,STAT3 |
| Systemic Lupus Erythematosus In T Cell Signaling Pathway | 0.407 | 0.033 | 0.905 | BCL6,CASP7,CD44,DIRAS3,GADD45A,PDK1,RASD1,RORC,S1PR3,SELPLG,STAT3 |
| Role of Oct4 in Mammalian Embryonic Stem Cell Pluripotency | 0.405 | 0.0435 |  | SPP1,TP53 |
| Dermatan Sulfate Biosynthesis (Late Stages) | 0.405 | 0.0435 |  | CHST3,SULT1C4 |
| Stearate Biosynthesis I (Animals) | 0.405 | 0.0435 |  | ACSL5,SLC27A3 |
| Antiproliferative Role of Somatostatin Receptor 2 | 0.401 | 0.039 |  | CDKN1A,RASD1,SSTR2 |
| Ubiquinol-10 Biosynthesis (Eukaryotic) | 0.399 | 0.0588 |  | CYP4F11 |
| Dermatan Sulfate Degradation (Metazoa) | 0.399 | 0.0588 |  | FGFRL1 |
| Histamine Degradation | 0.399 | 0.0588 |  | ALDH4A1 |
| CREB Signaling in Neurons | 0.387 | 0.0338 | 0.816 | ADCY2,GRM3,PLCD1,PLCE1,PRKCH,RASD1,SHC1 |
| T Cell Exhaustion Signaling Pathway | 0.385 | 0.0343 | 0.447 | BCL6,JAK3,PDK1,RASD1,STAT3,VEGFA |
| Chondroitin Sulfate Biosynthesis (Late Stages) | 0.382 | 0.0417 |  | CHST3,SULT1C4 |
| Purine Nucleotides Degradation II (Aerobic) | 0.381 | 0.0556 |  | GDA |
| mTOR Signaling | 0.378 | 0.0335 | -1 | DDIT4,DIRAS3,HMOX1,PLD4,PRKCH,RASD1,VEGFA |
| Role of BRCA1 in DNA Damage Response | 0.377 | 0.0375 |  | CDKN1A,GADD45A,TP53 |
| IL-17 Signaling | 0.377 | 0.0375 |  | CEBPB,RASD1,TIMP1 |
| FLT3 Signaling in Hematopoietic Progenitor Cells | 0.377 | 0.0375 |  | RASD1,SHC1,STAT3 |
| Renal Cell Carcinoma Signaling | 0.377 | 0.0375 |  | MET,RASD1,VEGFA |
| Chemokine Signaling | 0.377 | 0.0375 |  | CAMK1G,LIMK2,RASD1 |
| Rac Signaling | 0.376 | 0.0357 |  | CD44,ITGA5,LIMK2,RASD1 |
| GPCR-Mediated Nutrient Sensing in Enteroendocrine Cells | 0.376 | 0.0357 | 1 | ADCY2,PLCD1,PLCE1,PRKCH |
| Granzyme A Signaling | 0.364 | 0.0526 |  | HMGB2 |
| Oxidative Ethanol Degradation III | 0.364 | 0.0526 |  | ALDH4A1 |
| DNA damage-induced 14-3-3σ Signaling | 0.364 | 0.0526 |  | TP53 |
| Apelin Muscle Signaling Pathway | 0.364 | 0.0526 |  | APLNR |
| NGF Signaling | 0.363 | 0.0351 |  | MAP3K8,RASD1,SHC1,TP53 |
| Apelin Adipocyte Signaling Pathway | 0.361 | 0.0366 |  | ADCY2,APLNR,MGST1 |
| Endocannabinoid Developing Neuron Pathway | 0.357 | 0.0348 |  | ADCY2,CNR1,RASD1,STAT3 |
| Role of NFAT in Regulation of the Immune Response | 0.354 | 0.0331 | 1 | AKAP5,BLNK,FCGR2A,FCGR3A/FCGR3B,NFKBIA,RASD1 |
| The Visual Cycle | 0.347 | 0.05 |  | DHRS3 |
| Fatty Acid α-oxidation | 0.347 | 0.05 |  | ALDH4A1 |
| UVB-Induced MAPK Signaling | 0.341 | 0.0385 |  | PRKCH,TP53 |
| CD27 Signaling in Lymphocytes | 0.333 | 0.0377 |  | MAP3K8,NFKBIA |
| Lymphotoxin β Receptor Signaling | 0.333 | 0.0377 |  | NFKBIA,TRAF1 |
| Endoplasmic Reticulum Stress Pathway | 0.332 | 0.0476 |  | CASP7 |
| Transcriptional Regulatory Network in Embryonic Stem Cells | 0.323 | 0.037 |  | RFX4,STAT3 |
| D-myo-inositol-5-phosphate Metabolism | 0.317 | 0.0323 | 1.342 | ALPL,NUDT11,PLCD1,PLCE1,SOCS3 |
| Methionine Degradation I (to Homocysteine) | 0.317 | 0.0455 |  | AHCYL1 |
| EGF Signaling | 0.314 | 0.0364 |  | SHC1,STAT3 |
| EIF2 Signaling | 0.313 | 0.0314 | 0 | ACTA2,ATF3,NKX6-2,PPP1R15A,RASD1,SHC1,VEGFA |
| Crosstalk between Dendritic Cells and Natural Killer Cells | 0.31 | 0.0337 |  | ACTA2,TNFRSF1B,TNFSF10 |
| Chondroitin Sulfate Biosynthesis | 0.306 | 0.0357 |  | CHST3,SULT1C4 |
| Altered T Cell and B Cell Signaling in Rheumatoid Arthritis | 0.304 | 0.0333 |  | CSF1,SPP1,TLR2 |
| Ethanol Degradation IV | 0.304 | 0.0435 |  | ALDH4A1 |
| CNTF Signaling | 0.298 | 0.0351 |  | RASD1,STAT3 |
| Role of CHK Proteins in Cell Cycle Checkpoint Control | 0.298 | 0.0351 |  | CDKN1A,TP53 |
| ERK/MAPK Signaling | 0.297 | 0.0311 | 2.236 | HSPB1,ITGA5,PXN,RASD1,SHC1,STAT3 |
| Role of JAK1, JAK2 and TYK2 in Interferon Signaling | 0.291 | 0.0417 |  | STAT3 |
| Glutathione Redox Reactions I | 0.291 | 0.0417 |  | MGST1 |
| Cysteine Biosynthesis III (mammalia) | 0.291 | 0.0417 |  | AHCYL1 |
| Factors Promoting Cardiogenesis in Vertebrates | 0.285 | 0.0323 |  | PRKCH,TCF7L2,TGFB2 |
| Dermatan Sulfate Biosynthesis | 0.282 | 0.0339 |  | CHST3,SULT1C4 |
| Melanocyte Development and Pigmentation Signaling | 0.279 | 0.0319 |  | ADCY2,RASD1,SHC1 |
| Bupropion Degradation | 0.278 | 0.04 |  | CYP1B1 |
| Melatonin Degradation I | 0.275 | 0.0333 |  | CYP1B1,SULT1C4 |
| PCP pathway | 0.275 | 0.0333 |  | CELSR1,WNT5A |
| Estrogen-mediated S-phase Entry | 0.267 | 0.0385 |  | CDKN1A |
| Role of p14/p19ARF in Tumor Suppression | 0.237 | 0.0345 |  | TP53 |
| Mitotic Roles of Polo-Like Kinase | 0.234 | 0.0303 |  | PLK2,PLK3 |
| Acetone Degradation I (to Methylglyoxal) | 0.228 | 0.0333 |  | CYP1B1 |
| Glutathione-mediated Detoxification | 0.21 | 0.0312 |  | MGST1 |
| Ethanol Degradation II | 0.21 | 0.0312 |  | ALDH4A1 |
| Circadian Rhythm Signaling | 0.203 | 0.0303 |  | GRIN3A |
| Retinoate Biosynthesis I | 0.203 | 0.0303 |  | DHRS3 |
| Fc Epsilon RI Signaling | 0 | 0.0171 |  | PRKCH,RASD1 |
| Mitochondrial Dysfunction | 0 | 0.0234 |  | BACE2,MAOA,MAOB,SOD2 |
| PXR/RXR Activation | 0 | 0.0154 |  | PAPSS2 |
| Activation of IRF by Cytosolic Pattern Recognition Receptors | 0 | 0.0159 |  | NFKBIA |
| Role of RIG1-like Receptors in Antiviral Innate Immunity | 0 | 0.0227 |  | NFKBIA |
| CCR5 Signaling in Macrophages | 0 | 0.0106 |  | PRKCH |
| Calcium-induced T Lymphocyte Apoptosis | 0 | 0.0152 |  | PRKCH |
| Cytotoxic T Lymphocyte-mediated Apoptosis of Target Cells | 0 | 0.0294 |  | CASP7 |
| fMLP Signaling in Neutrophils | 0 | 0.0259 |  | NFKBIA,PRKCH,RASD1 |
| CCR3 Signaling in Eosinophils | 0 | 0.0242 |  | LIMK2,PRKCH,RASD1 |
| CD28 Signaling in T Helper Cells | 0 | 0.00833 |  | NFKBIA |
| Relaxin Signaling | 0 | 0.0267 | 0 | ADCY2,NFKBIA,RXFP1,VEGFA |
| CDK5 Signaling | 0 | 0.0278 |  | ADCY2,LAMA5,RASD1 |
| iCOS-iCOSL Signaling in T Helper Cells | 0 | 0.018 |  | NFKBIA,SHC1 |
| Corticotropin Releasing Hormone Signaling | 0 | 0.0276 |  | ADCY2,CNR1,PRKCH,VEGFA |
| GNRH Signaling | 0 | 0.0289 | 1 | ADCY2,MAP3K8,PRKCH,PXN,RASD1 |
| Androgen Signaling | 0 | 0.0221 |  | DNAJB1,PRKCH,SHC1 |
| Communication between Innate and Adaptive Immune Cells | 0 | 0.0104 |  | TLR2 |
| Systemic Lupus Erythematosus Signaling | 0 | 0.0262 |  | C7,FCGR2A,FCGR3A/FCGR3B,RASD1,RNU12,RNU4-1 |
| Cdc42 Signaling | 0 | 0.018 |  | ITGA5,LIMK2,MYL9 |
| AMPK Signaling | 0 | 0.00943 |  | CDKN1A,GYS1 |
| Regulation of eIF4 and p70S6K Signaling | 0 | 0.0192 |  | ITGA5,RASD1,SHC1 |
| Estrogen-Dependent Breast Cancer Signaling | 0 | 0.027 |  | IGF1,RASD1 |
| RANK Signaling in Osteoclasts | 0 | 0.0227 |  | MAP3K8,NFKBIA |
| Role of Wnt/GSK-3β Signaling in the Pathogenesis of Influenza | 0 | 0.0256 |  | TCF7L2,WNT5A |
| PKCθ Signaling in T Lymphocytes | 0 | 0.0194 |  | MAP3K8,NFKBIA,RASD1 |
| Role of PI3K/AKT Signaling in the Pathogenesis of Influenza | 0 | 0.0156 |  | NFKBIA |
| OX40 Signaling Pathway | 0 | 0.0111 |  | NFKBIA |
| Role of IL-17A in Arthritis | 0 | 0.0185 |  | NFKBIA |
| Role of IL-17F in Allergic Inflammatory Airway Diseases | 0 | 0.0238 |  | IGF1 |
| Hematopoiesis from Pluripotent Stem Cells | 0 | 0.0204 |  | CSF1 |
| Ephrin A Signaling | 0 | 0.0213 |  | EFNA1 |
| Ephrin B Signaling | 0 | 0.0278 |  | EPHB2,PXN |
| GDNF Family Ligand-Receptor Interactions | 0 | 0.0263 |  | RASD1,SHC1 |
| Heparan Sulfate Biosynthesis | 0 | 0.0256 |  | CHST3,SULT1C4 |
| Thyroid Hormone Metabolism II (via Conjugation and/or Degradation) | 0 | 0.0263 |  | SULT1C4 |
| Nicotine Degradation III | 0 | 0.0179 |  | CYP1B1 |
| Heparan Sulfate Biosynthesis (Late Stages) | 0 | 0.0282 |  | CHST3,SULT1C4 |
| Pyrimidine Ribonucleotides De Novo Biosynthesis | 0 | 0.0233 |  | ENTPD2 |
| Triacylglycerol Degradation | 0 | 0.0213 |  | LPL |
| Estrogen Biosynthesis | 0 | 0.0244 |  | CYP1B1 |
| D-myo-inositol (1,4,5,6)-Tetrakisphosphate Biosynthesis | 0 | 0.0214 |  | ALPL,NUDT11,SOCS3 |
| Nicotine Degradation II | 0 | 0.0154 |  | CYP1B1 |
| D-myo-inositol (3,4,5,6)-tetrakisphosphate Biosynthesis | 0 | 0.0214 |  | ALPL,NUDT11,SOCS3 |
| 3-phosphoinositide Degradation | 0 | 0.0195 |  | ALPL,NUDT11,SOCS3 |
| 3-phosphoinositide Biosynthesis | 0 | 0.0183 |  | ALPL,NUDT11,SOCS3 |
| Triacylglycerol Biosynthesis | 0 | 0.0244 |  | LPIN3 |
| Pyrimidine Ribonucleotides Interconversion | 0 | 0.0244 |  | ENTPD2 |
| Superpathway of Methionine Degradation | 0 | 0.027 |  | AHCYL1 |
| Gαs Signaling | 0 | 0.028 |  | ADCY2,CNR1,RGS2 |
| HIPPO signaling | 0 | 0.0235 |  | CD44,WWTR1 |
| Estrogen Receptor Signaling | 0 | 0.0146 |  | RASD1,SHC1 |
| Cardiac β-adrenergic Signaling | 0 | 0.0142 |  | ADCY2,AKAP5 |
| Protein Ubiquitination Pathway | 0 | 0.0183 |  | DNAJB1,HSPA1A/HSPA1B,HSPB1,HSPB8,TAP1 |
| Amyloid Processing | 0 | 0.02 |  | BACE2 |
| Neurotrophin/TRK Signaling | 0 | 0.0263 |  | RASD1,SHC1 |
| TGF-β Signaling | 0 | 0.0208 |  | RASD1,TGFB2 |
| T Cell Receptor Signaling | 0 | 0.019 |  | NFKBIA,RASD1 |
| BMP signaling pathway | 0 | 0.0118 |  | RASD1 |
| Gustation Pathway | 0 | 0.026 |  | ADCY2,P2RY1,P2RY12,P2RY13 |
| Phagosome Maturation | 0 | 0.0265 |  | CTSH,TAP1,TUBA1C,TUBA4B |
| Autophagy | 0 | 0.0164 |  | CTSH |
| Sumoylation Pathway | 0 | 0.0291 |  | DIRAS3,NFKBIA,TP53 |
| Th2 Pathway | 0 | 0.0294 |  | ICAM1,IL4R,JAK3,SOCS3 |
| SPINK1 Pancreatic Cancer Pathway | 0 | 0.0167 |  | CPM |
| Apelin Pancreas Signaling Pathway | 0 | 0.0227 |  | APLNR |
