# Supplementary material for: RNA-sequencing suggests extracellular matrix and vasculature dysregulation could impair neurogenesis in schizophrenia cases with elevated inflammation
Source: Schizophrenia (Heidelb). 2024 May 4;10(1):50. doi: 10.1038/s41537-024-00466-0 (PMC11069512; doi:10.1038/s41537-024-00466-0)
Supplement: Supplementary file 4 — Appendix Table 4 [file 41537_2024_466_MOESM4_ESM.docx]

**Appendix Table 4**. Spearman's correlation analyses between DE genes in the significant extracellular matrix pathway and markers for neurogenesis.

| Positive  Correlation | Negative  Correlation | Neural Stem Cell Markers | | | | | | | Neuroblast Markers | | | | | | | Immature Neuron Makers | | | |
| --- | --- | --- | --- | --- | --- | --- | --- | --- | --- | --- | --- | --- | --- | --- | --- | --- | --- | --- | --- |
| Extracellular Matrix Genes | | SOX2 | PAX6 | GLI3 | MKI67 | HES5 | LFNG | ASCL1 | CCND2 | CDC42 | TMSB10 | NNAT | CDK10 | FGFR3 | CEND1 | DLX6-AS1 | DCX | DLX1 | PDGFD |
| COL1A1 | Spearman's rho | 0.204 | 0.128 | **.514^**^** | 0.298 | -0.226 | 0.322 | -0.136 | 0.261 | -0.231 | -0.203 | -0.310 | 0.286 | 0.110 | 0.114 | -0.154 | 0.194 | 0.202 | -0.139 |
|  | p vaue | 0.307 | 0.524 | 0.006 | 0.131 | 0.256 | 0.101 | 0.498 | 0.188 | 0.247 | 0.310 | 0.116 | 0.148 | 0.586 | 0.573 | 0.444 | 0.333 | 0.313 | 0.489 |
| COL1A2 | Spearman's rho | **.626^**^** | **.626^**^** | 0.290 | **.564^**^** | **-.604^**^** | -0.256 | .430^*^ | 0.059 | **.521^**^** | -0.198 | -0.259 | -0.053 | **-.567^**^** | **-.537^**^** | -0.250 | 0.034 | 0.198 | -0.006 |
|  | p vaue | 0.000 | 0.000 | 0.142 | 0.002 | 0.001 | 0.197 | 0.025 | 0.772 | 0.005 | 0.321 | 0.192 | 0.792 | 0.002 | 0.004 | 0.208 | 0.866 | 0.323 | 0.977 |
| COL27A1 | Spearman's rho | **.533^**^** | .424^*^ | **.601^**^** | 0.191 | -0.318 | 0.134 | 0.214 | 0.166 | 0.078 | -0.353 | -0.232 | 0.256 | -0.011 | -0.139 | -0.323 | 0.123 | 0.324 | -0.130 |
|  | p vaue | 0.004 | 0.028 | 0.001 | 0.339 | 0.106 | 0.506 | 0.283 | 0.408 | 0.701 | 0.071 | 0.245 | 0.197 | 0.955 | 0.489 | 0.100 | 0.540 | 0.099 | 0.518 |
| COL3A1 | Spearman's rho | **.734^**^** | **.646^**^** | 0.151 | .420^*^ | **-.549^**^** | -0.124 | .421^*^ | -0.156 | **.526^**^** | -0.325 | -0.253 | -0.147 | -.432^*^ | -.471^*^ | -.397^*^ | -0.143 | 0.009 | -0.194 |
|  | p vaue | 0.000 | 0.000 | 0.453 | 0.029 | 0.003 | 0.539 | 0.029 | 0.436 | 0.005 | 0.098 | 0.202 | 0.464 | 0.025 | 0.013 | 0.040 | 0.475 | 0.965 | 0.332 |
| COL4A1 | Spearman's rho | 0.315 | 0.198 | 0.115 | 0.214 | **-.820^**^** | -0.039 | -0.123 | 0.153 | 0.076 | 0.044 | **-.667^**^** | 0.064 | **-.556^**^** | -0.258 | -.434^*^ | -0.075 | 0.031 | -0.051 |
|  | p vaue | 0.109 | 0.321 | 0.569 | 0.283 | 0.000 | 0.847 | 0.542 | 0.446 | 0.706 | 0.826 | 0.000 | 0.752 | 0.003 | 0.194 | 0.024 | 0.711 | 0.880 | 0.801 |
| COL4A2 | Spearman's rho | **.518^**^** | .435^*^ | 0.162 | 0.279 | **-.669^**^** | 0.105 | 0.127 | 0.108 | 0.306 | -0.082 | **-.629^**^** | 0.114 | -.426^*^ | -0.209 | -.491^**^ | -0.165 | 0.132 | -0.054 |
|  | p vaue | 0.006 | 0.024 | 0.420 | 0.159 | 0.000 | 0.601 | 0.529 | 0.590 | 0.121 | 0.685 | 0.000 | 0.572 | 0.027 | 0.296 | 0.009 | 0.412 | 0.511 | 0.789 |
| COL5A1 | Spearman's rho | **.560^**^** | 0.380 | **.525^**^** | 0.063 | -0.357 | 0.142 | 0.159 | 0.117 | 0.083 | -.441^*^ | -0.230 | 0.073 | -0.027 | -0.216 | -0.347 | 0.062 | 0.061 | -0.260 |
|  | p vaue | 0.002 | 0.050 | 0.005 | 0.755 | 0.068 | 0.481 | 0.429 | 0.562 | 0.681 | 0.021 | 0.249 | 0.719 | 0.894 | 0.280 | 0.076 | 0.760 | 0.762 | 0.191 |
| COL6A3 | Spearman's rho | **.614^**^** | **.549^**^** | .460^*^ | .466^*^ | **-.617^**^** | -0.080 | 0.205 | 0.120 | 0.321 | -0.161 | -.441^*^ | 0.105 | -0.369 | -0.314 | -0.330 | 0.039 | 0.308 | 0.056 |
|  | p vaue | 0.001 | 0.003 | 0.016 | 0.014 | 0.001 | 0.690 | 0.306 | 0.551 | 0.103 | 0.422 | 0.021 | 0.603 | 0.058 | 0.111 | 0.092 | 0.845 | 0.118 | 0.782 |
| COL7A1 | Spearman's rho | 0.274 | 0.300 | **.582^**^** | 0.257 | -0.264 | 0.184 | -0.015 | 0.249 | -0.107 | -0.128 | **-.518^**^** | 0.283 | 0.057 | 0.081 | -0.180 | 0.139 | 0.347 | 0.067 |
|  | p vaue | 0.166 | 0.128 | 0.001 | 0.195 | 0.183 | 0.359 | 0.942 | 0.210 | 0.594 | 0.524 | 0.006 | 0.153 | 0.776 | 0.687 | 0.369 | 0.489 | 0.076 | 0.739 |
| COL8A1 | Spearman's rho | **.649^**^** | .464^*^ | 0.338 | 0.222 | **-.630^**^** | -0.087 | 0.288 | -0.144 | 0.358 | -.465^*^ | -0.326 | -0.145 | -.414^*^ | -.498^**^ | **-.521^**^** | -0.167 | 0.023 | -0.196 |
|  | p vaue | 0.000 | 0.015 | 0.085 | 0.266 | 0.000 | 0.667 | 0.145 | 0.474 | 0.066 | 0.015 | 0.097 | 0.470 | 0.032 | 0.008 | 0.005 | 0.404 | 0.910 | 0.326 |
| FN1 | Spearman's rho | 0.172 | 0.305 | 0.302 | .383^*^ | **-.562^**^** | -.396^*^ | 0.087 | .405^*^ | 0.319 | 0.004 | -0.104 | 0.328 | -.453^*^ | **-.508^**^** | -0.041 | 0.173 | -0.078 | -0.055 |
|  | p vaue | 0.391 | 0.122 | 0.126 | 0.049 | 0.002 | 0.041 | 0.665 | 0.036 | 0.105 | 0.986 | 0.604 | 0.094 | 0.018 | 0.007 | 0.839 | 0.389 | 0.698 | 0.787 |
| CCN2 | Spearman's rho | **.596^**^** | .395^*^ | 0.253 | 0.268 | **-.600^**^** | 0.047 | 0.280 | -0.209 | 0.372 | -.438^*^ | -0.321 | -0.252 | -.476^*^ | -.496^**^ | -.487^*^ | -0.152 | -0.072 | -0.239 |
|  | p vaue | 0.001 | 0.041 | 0.203 | 0.176 | 0.001 | 0.815 | 0.158 | 0.295 | 0.056 | 0.022 | 0.102 | 0.206 | 0.012 | 0.008 | 0.010 | 0.449 | 0.722 | 0.230 |
| TIMP1 | Spearman's rho | .429^*^ | 0.325 | 0.126 | 0.287 | **-.767^**^** | -0.005 | 0.025 | 0.047 | 0.143 | -0.247 | **-.739^**^** | -0.035 | -.458^*^ | -0.289 | **-.565^**^** | -0.237 | -0.028 | -0.142 |
|  | p vaue | 0.026 | 0.098 | 0.532 | 0.147 | 0.000 | 0.982 | 0.900 | 0.816 | 0.477 | 0.215 | 0.000 | 0.863 | 0.016 | 0.143 | 0.002 | 0.233 | 0.891 | 0.481 |
| TGFB2 | Spearman's rho | **.811^**^** | **.753^**^** | 0.340 | 0.250 | **-.557^**^** | -0.191 | **.640^**^** | -0.001 | **.616^**^** | -.481^*^ | -0.274 | -0.049 | **-.556^**^** | **-.688^**^** | -.458^*^ | -0.131 | 0.088 | -0.129 |
|  | p vaue | 0.000 | 0.000 | 0.083 | 0.208 | 0.003 | 0.341 | 0.000 | 0.998 | 0.001 | 0.011 | 0.166 | 0.809 | 0.003 | 0.000 | 0.016 | 0.516 | 0.662 | 0.522 |
