# Supplementary material for: RNA-sequencing suggests extracellular matrix and vasculature dysregulation could impair neurogenesis in schizophrenia cases with elevated inflammation
Source: Schizophrenia (Heidelb). 2024 May 4;10(1):50. doi: 10.1038/s41537-024-00466-0 (PMC11069512; doi:10.1038/s41537-024-00466-0)
Supplement: Supplementary file 5 — Appendix Table 5 [file 41537_2024_466_MOESM5_ESM.docx]

**Appendix Table 5**. Spearman's correlation analyses between significant angiogenesis related DE genes and markers for neurogenesis.

| Positive  Correlation | Negative  Correlation | Neural Stem Cell Markers | | | | | | | Neuroblast Markers | | | | | | | Immature Neuron Makers | | | |
| --- | --- | --- | --- | --- | --- | --- | --- | --- | --- | --- | --- | --- | --- | --- | --- | --- | --- | --- | --- |
| Angiogenesis Genes | | SOX2 | PAX6 | GLI3 | MKI67 | HES5 | LFNG | ASCL1 | CCND2 | CDC42 | TMSB10 | NNAT | CDK10 | FGFR3 | CEND1 | DLX6-AS1 | DCX | DLX1 | PDGFD |
| VEGFA | Correlation Coefficient | 0.338 | 0.302 | .411^*^ | 0.247 | -.436^*^ | -0.063 | 0.033 | 0.364 | 0.084 | 0.211 | -0.282 | 0.258 | -0.252 | -0.062 | -0.063 | 0.361 | .408^*^ | 0.042 |
|  | Sig. (2-tailed) | 0.085 | 0.126 | 0.033 | 0.213 | 0.023 | 0.754 | 0.872 | 0.062 | 0.678 | 0.290 | 0.154 | 0.195 | 0.206 | 0.760 | 0.755 | 0.064 | 0.035 | 0.833 |
| FLT1 | Correlation Coefficient | 0.201 | 0.229 | 0.247 | 0.327 | **-.576^**^** | -0.272 | -0.102 | .407^*^ | 0.145 | 0.323 | -0.301 | 0.249 | -.470^*^ | -0.206 | -0.009 | 0.291 | 0.156 | 0.091 |
|  | Sig. (2-tailed) | 0.315 | 0.251 | 0.215 | 0.096 | 0.002 | 0.170 | 0.613 | 0.035 | 0.470 | 0.100 | 0.127 | 0.210 | 0.013 | 0.303 | 0.966 | 0.141 | 0.438 | 0.653 |
| FLT4 | Correlation Coefficient | -0.013 | -0.073 | .418^*^ | 0.210 | 0.099 | 0.227 | -0.239 | 0.137 | -.409^*^ | 0.179 | -0.325 | 0.121 | 0.166 | 0.379 | 0.019 | 0.226 | 0.269 | 0.196 |
|  | Sig. (2-tailed) | 0.947 | 0.716 | 0.030 | 0.293 | 0.624 | 0.255 | 0.230 | 0.494 | 0.034 | 0.372 | 0.098 | 0.548 | 0.408 | 0.051 | 0.925 | 0.257 | 0.175 | 0.326 |
| EDN1 | Correlation Coefficient | 0.059 | 0.012 | -0.169 | 0.053 | **-.674^**^** | -0.076 | -0.216 | 0.121 | 0.233 | 0.218 | **-.520^**^** | -0.093 | **-.601^**^** | -0.203 | -0.224 | -0.098 | -0.220 | 0.030 |
|  | Sig. (2-tailed) | 0.770 | 0.953 | 0.399 | 0.792 | 0.000 | 0.707 | 0.280 | 0.549 | 0.242 | 0.275 | 0.005 | 0.645 | 0.001 | 0.309 | 0.261 | 0.626 | 0.269 | 0.882 |
| ECE1 | Correlation Coefficient | **.542^**^** | **.546^**^** | **.557^**^** | .441^*^ | **-.555^**^** | 0.102 | 0.209 | 0.258 | .392^*^ | -0.066 | -.473^*^ | 0.303 | -.427^*^ | -0.333 | -0.313 | 0.071 | 0.238 | -0.032 |
|  | Sig. (2-tailed) | 0.003 | 0.003 | 0.003 | 0.021 | 0.003 | 0.614 | 0.297 | 0.195 | 0.043 | 0.744 | 0.013 | 0.124 | 0.026 | 0.089 | 0.112 | 0.726 | 0.232 | 0.875 |
| MYH11 | Correlation Coefficient | **.509^**^** | .454^*^ | **.656^**^** | **.548^**^** | -0.278 | 0.056 | 0.168 | 0.220 | 0.159 | -0.047 | -0.310 | 0.158 | -0.094 | -0.084 | -0.140 | 0.280 | .451^*^ | 0.088 |
|  | Sig. (2-tailed) | 0.007 | 0.017 | 0.000 | 0.003 | 0.161 | 0.781 | 0.402 | 0.271 | 0.428 | 0.816 | 0.115 | 0.433 | 0.641 | 0.678 | 0.485 | 0.157 | 0.018 | 0.661 |
| MYL9 | Correlation Coefficient | 0.341 | 0.212 | .426^*^ | **.522^**^** | -0.285 | 0.071 | -0.055 | 0.021 | -0.071 | -0.034 | **-.526^**^** | -0.075 | -0.084 | 0.134 | -0.327 | 0.038 | 0.286 | -0.010 |
|  | Sig. (2-tailed) | 0.081 | 0.289 | 0.027 | 0.005 | 0.150 | 0.726 | 0.784 | 0.916 | 0.726 | 0.866 | 0.005 | 0.710 | 0.676 | 0.504 | 0.096 | 0.849 | 0.148 | 0.961 |
| ACTA2 | Correlation Coefficient | **.507^**^** | .473^*^ | 0.240 | **.554^**^** | **-.560^**^** | -0.114 | 0.165 | 0.098 | 0.294 | -0.054 | -.491^**^ | -0.024 | **-.513^**^** | -0.273 | -0.300 | -0.051 | 0.089 | -0.074 |
|  | Sig. (2-tailed) | 0.007 | 0.013 | 0.228 | 0.003 | 0.002 | 0.570 | 0.410 | 0.628 | 0.136 | 0.790 | 0.009 | 0.906 | 0.006 | 0.168 | 0.128 | 0.799 | 0.661 | 0.714 |
